# Water Effect on the Photochemistry of Arylazo Sulfonates

**DOI:** 10.1021/acs.joc.5c00314

**Published:** 2025-04-30

**Authors:** Luca Nicchio, Hawraz Ibrahim M. Amin, Stefano Genualdo, Stefano Protti, Maurizio Fagnoni

**Affiliations:** † PhotoGreen Lab, Department of Chemistry, 19001 University of Pavia, Viale Taramelli 12, 27100 Pavia, Italy; ‡ Institut de Chimie des Substances Naturelles (ICSN), CNRS UPR 2301, Université Paris-Saclay, 1 Avenue de La Terrasse, 91198 Gif-sur-Yvette Cedex, France; § Department of Chemistry, College of Science, Salahaddin University-Erbil, 44001 Erbil, Iraq

## Abstract

The effect of water on visible-light-driven generation of aryl radicals or aryl cations from colored shelf-stable arylazo sulfonates has been investigated. Photoinduced ionic and radical decomposition of these salts compete, depending on the media used. In organic solvents, light-induced homolysis of the N–S bond occurs, and the resulting aryl radical may be used to some extent for arylation reactions. On the contrary, in neat water, radical chemistry is prevented by an efficient photoheterolysis, and a reactive aryl cation is otherwise generated.

## Graphical abstract

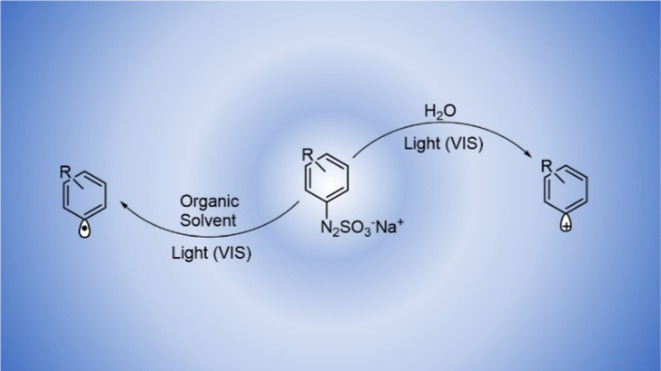

## Introduction

Water is considered the elective green medium since it is inexpensive, nonflammable, and benign.^1^ As for organic synthesis, depending on the solubility of the reagents, “in water” (where water is the exclusive solvent, and the solution is homogeneous) or “on water”^2^ (the substrates are virtually insoluble under these conditions) reactions may take place. However, there are many cases in between, where reactions are carried out “in the presence of water”, in “aqueous mixtures”, or “in emulsions”.^3^ Nevertheless, there is a debate on the real greenness and utility of water as (a component of) the reaction medium. Indeed, most organic compounds are poorly soluble in water, and only a narrow scope of the transformations is so far developed. Moreover, product isolation by extraction forcedly required a huge amount of an organic solvent (thus affecting the environmental impact of the procedure).^3,4^ Furthermore, sometimes the “on water” effect on the reaction rate is negligible.^3,5^

The influence of water on the generation and the reactivity of high-energy intermediates (e.g., radicals and radical ions) is another point of discussion, especially since photochemistry and/or photocatalysis opened new ways for the selective and mild generation of such species. The widespread use of visible light has widened the application of these radical processes to nonstrict adherence photochemistry practitioners even adopting water as the reaction medium.^6^ Accordingly, to achieve the desired target, a chemist may induce the radical generation with a colored photocatalyst^7^ or having recourse to the photoreactivity of a colored chromophore present in the starting compound.^8^

A rather recent strategy involves the incorporation of a photolabile colored moiety (the so-called dyedauxiliary group) able to release radicals upon visible-light absorption.^8,9a^ A prototypical case is that of arylazo sulfones **I** (Scheme 1a),^9^ obtained from anilines via the intermediacy of an aryl diazonium salt. Such yellow-orange crystalline shelf stable compounds are sensitive to the exposure to blue LEDs (λ = 427 nm) that induces a nitrogen loss with the concomitant liberation of an aryl and a sulfonyl radical. Arylazo sulfones have been thus used for both arylations^9,10^ and sulfonylations^11^ along with applications in material chemistry^12^ or as photoacid generators (PAGs).^13^ All of the reported processes were carried out in organic media (or at least in water/organic solvent mixtures) imposed by the solubility of the sulfones.

**Scheme 1:**
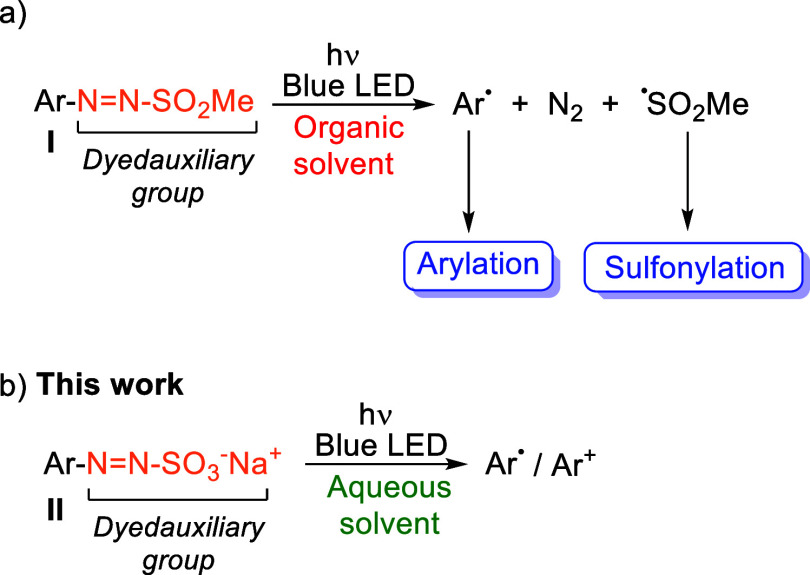
Azo Sulfone (a) or Azo Sulfonate Group (b) in Dyedauxiliary Group Strategy

In view of these premises, we thus wonder if radical chemistry derived by a dyedauxiliary strategy^9a^ may be shifted to water or aqueous/organic mixtures by designing a suitable functional group. A literature survey indicates that incorporating a sulfonate group in arylazo sulfonate **II** may be the ideal choice (Scheme 1b). Compounds **II** are known since 1869 ^14^ but only sparsely used as photoresponsive surfactants^15^ or in polymer chemistry.^16^

The complete (or partial) substitution of organic solvents with water may have several advantages. Despite extraordinary solvent effects that may be expected for water, the noncharged nature of radicals pointed to a negligible sensitivity of these species to aqueous medium. Thus, water is an intriguing medium for radical synthesis and in most cases a beneficial effect was observed on the reaction course.^17,18^ There are sparse examples of radical arylations in water, and in most cases the solvent did not hamper the overall efficiency^19^ with some exceptions.^20^ The main advantage of the use of water is that the competitive hydrogen abstraction of the aryl radical from the solvent is less efficient when compared to common organic media,^21^ although calculations demonstrated that hydrogen abstraction from water by a phenyl radical is still slightly exothermic (4.2 kcal mol^–1^).^22^ Contrary to arylazo sulfones **I**, the cleavage of the N–S bond is not accompanied by the generation of sulfur-based radicals, thus avoiding any competitive sulfonylations.^23^ Another intriguing issue is the possible competitive photogeneration of aryl cations rather than radicals due to the strong stabilization of the former intermediates exerted by water (Scheme 1b).

## Results

We thus prepared arylazo sulfonates **1a**–**1o** (Chart 1) with the aim to have insights on their (photo)­reactivity in view of their possible applications in synthesis, material science, or biology. These compounds have been isolated as yellow-orange solids (Figure S1) in up to quantitative yields by treatment of the corresponding aryl diazonium salts with Na_2_SO_3_ under controlled pH (ca. 7–8, see Experimental section).

**Chart 1:**
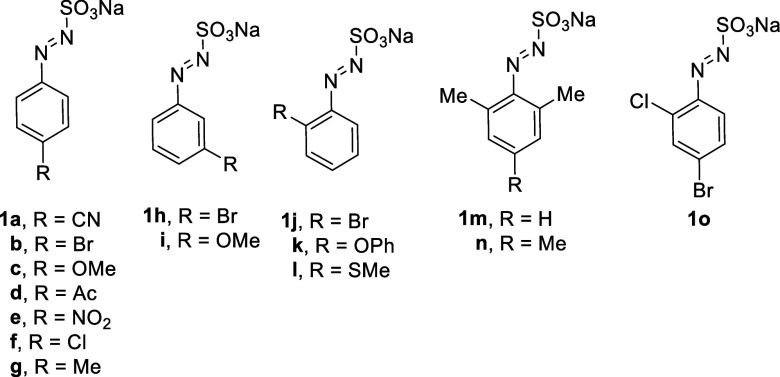
Arylazo Sulfonates Tested in This Work

Solubility tests on sulfonate **1c** revealed that this compound is fully soluble in water, methanol, or in alcohol/water mixtures along with Me_2_CO/H_2_O, MeCN/H_2_O, or 1,4-dioxane/H_2_O mixtures.

The UV–visible spectrum of **1c** in MeOH is shown in Figure 1 (the UV–vis spectra of other selected sulfonates may be found in Figure S2a–c). All of the sulfonates tested exhibit two absorption maxima, one located in the UV region (ca. 290–320 nm, ε = 4000–30,000 M^–1^ cm^–1^) and the other in the visible zone (ca. 390–430 nm, ε = 100–600 M^–1^ cm^–1^). The maximum absorption wavelengths and molar extinction coefficients of the tested sulfonates are reported in Table S1. The absorption in the UV region corresponds to the ππ* transition, whereas that in the visible region is due to the nπ* transition in analogy to the corresponding arylazo sulfones.^10^ The arylazo sulfonates examined do not exhibit any fluorescence in water (see Supporting Information for further details).

**Figure 1:**
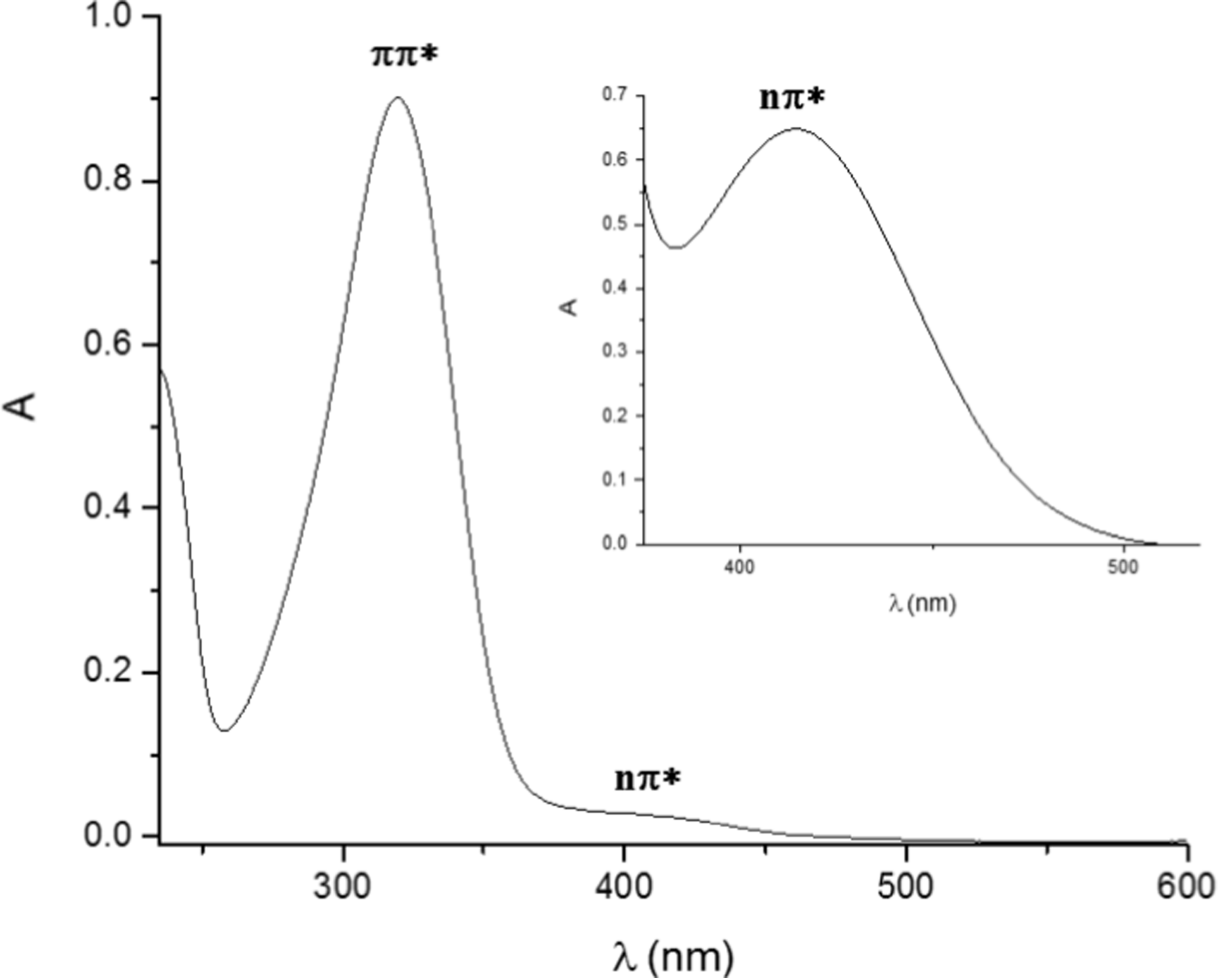
UV absorption spectrum of a 5 × 10^–5^ M solution of sodium 4-methoxyphenylazo sulfonate (**1c**) in methanol. Inset: absorption in the visible region (3 × 10^–3^ M).

We measured the quantum yield of decomposition of selected arylazo sulfonates **1a**–**c** in different media as shown in Table 1. We initially tested an organic/aqueous mixture (MeCN/water 4:1) as the medium. As apparent from Table 1, the photoreactivity of the substrates is significant (Φ_–1_ > 0.4) regardless of the substituents present on the aromatic ring. Further experiments on **1c** revealed that increasing the amount of water in the medium (MeCN/water 3:1) caused a slight decrease in Φ_–1_. Nevertheless, the photochemical consumption in other aqueous mixtures containing acetone or EtOH is still very efficient (Φ_–1_ = 0.65 in EtOH/H_2_O 2:1). Interestingly, the lower value determined is that in neat water (Φ_–1_ = 0.32) in accordance with what was previously observed.^24^ As a matter of fact, the photoreactivity of arylazo sulfonates is 1 order of magnitude higher with respect to the corresponding arylazo sulfones (Φ_–1_ mostly <0.05).^13a^

**Table 1:** Quantum Yields of Decomposition (Φ_–1_) of Arylazo Sulfonates **1a–c** in Different Media^a^

substrate	media	Φ_–1_
**1a**	MeCN/H_2_O (4:1)	0.46
**1b**	MeCN/H_2_O (4:1)	0.44
**1c**	MeCN/H_2_O (4:1)	0.51
**1c**	MeCN/H_2_O (3:1)	0.40
**1c**	Me_2_CO/H_2_O (9:1)	0.51
**1c**	EtOH/H_2_O (2:1)	0.65
**1c**	H_2_O	0.32

a Determined at 427 nm.

To have more insights into the speed of consumption, kinetic measurements were carried out on sulfonates **1a**, **1b**, and **1g** in a MeCN/H_2_O 4:1 mixture (Figures 2 and S10). The consumption of the sulfonates was rapid in the first stage of the reaction but several hours of irradiation were required for total disappearance of the substrate.

**Figure 2:**
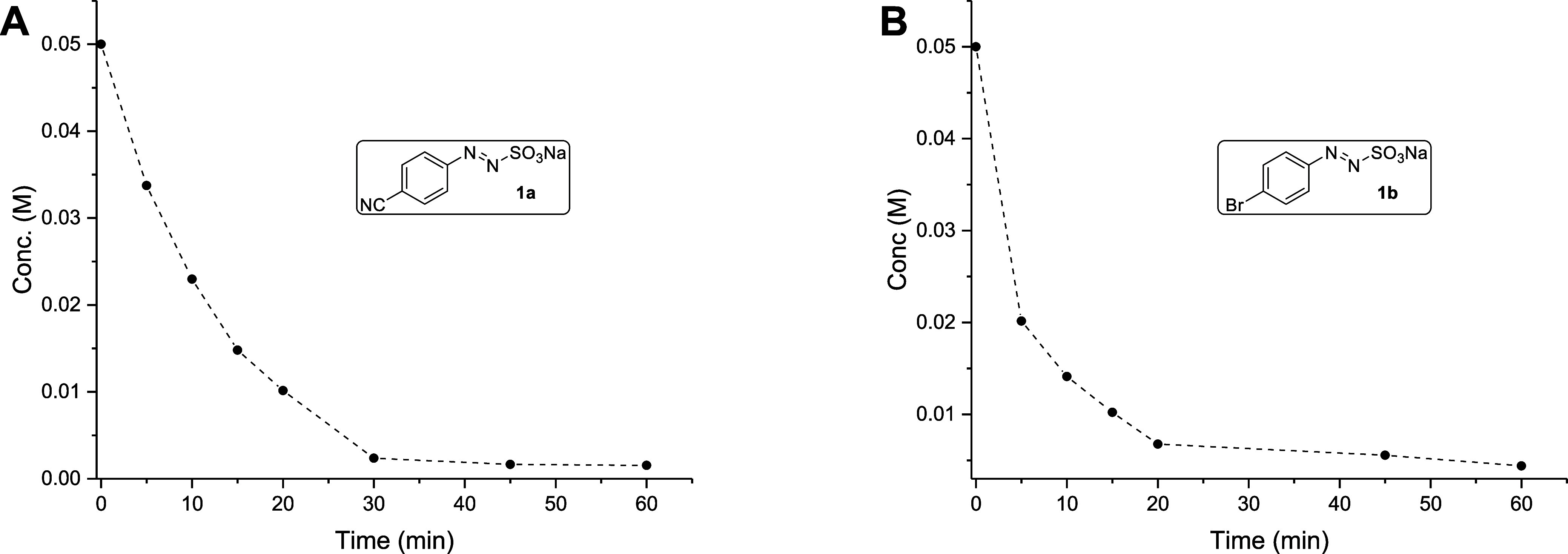
Kinetics of consumption of (A) sodium 4-cyanophenylazo sulfonate (**1a**, 0.05 M) and (B) sodium 4-bromophenylazo sulfonate (**1b**, 0.05 M) in a MeCN/H_2_O 4:1 mixture. Irradiation carried out with a 427 nm LED lamp.

We focused our attention on the product distribution obtained by photolysis in different reaction media (Table 2). The investigations were carried out on substrates **1a**–**1c** as models, prolonging the irradiation up to 24 h. As for **1a**, the consumption is not complete after the irradiation period, and the presence of EtOH or acetone will speed up the process (consumption always >90%). The reaction led consistently to benzonitrile **2a** albeit 4-cyanophenol **3a** was formed in some cases and quite exclusively in water. However, in MeCN/H_2_O (3:1) when the solution was deaerated with a freeze–pump–thaw method most of the phenol disappeared and **2a** became by far the main product (Table 2).

**Table 2:**
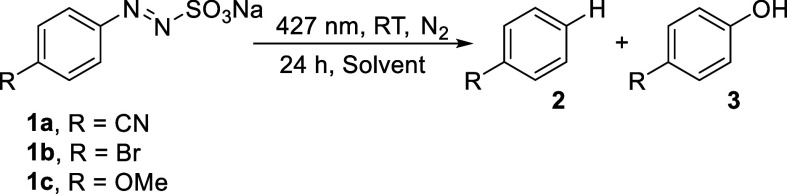
Products Distribution upon Visible-Light Irradiation of Arylazo Sulfonates **1a–1c** in Different Media

solvent	**1a** ^a^		**1b** ^a^		**1c** ^a^	
	conv. (%)^b^	products yield (%)^c^	conv. (%)^b^	products yield (%)^c^	conv. (%)^b^	products yield (%)^c^
MeCN/H_2_O (4:1)	73	**2a**, >99	98	**2b**, 78	96	**2c**, 86
MeCN/H_2_O (3:1)	98 (96)^d^	**2a**, 57 (87)^d^; **3a**, 22 (<5)^d^	97	**2b**, 82	91	**2c**, 82
EtOH/H_2_O (4:1)	95	**2a**, 51	98	**2b**, 68	99	**2c**, 79
EtOH/H_2_O (3:1)	93	**2a**, 42	99	**2b**, 62	99	**2c**, 99
Me_2_CO/H_2_O (4:1)	96	**2a**, 68	99	**2b**, 70	87	**2c**, 41
Me_2_CO/H_2_O (3:1)	94	**2a**, 92	86	**2b**, 81	84	**2c**, 80
H_2_O	77 (61)^d^	**3a**, 63 (50)^d^	54	**3b**, 32, **2b**, 11	73	**3c**, 61, **2c**, 8

a 0.05 M solution of **1a**–**c** irradiated with a 427 nm LED lamp after nitrogen bubbling.

b The consumption of **1** was quantified by means of HPLC analyses.

c Yields are based on the arylazo sulfonate consumed. GC yields for compounds **2a**–**c** and HPLC yields for **3a**–**c**.

d Solution deaerated with a freeze–pump–thaw method.

A similar behavior has been observed for arylazo sulfonates **1b** and **1c** where photoreduction is the exclusive pathway, again except in water where variable amounts of phenols **3** were detected along with small amounts of **2**. In each case, **1a**–**1c** are not consumed when dissolved in water or organic/water mixtures and kept in the dark at room temperature for (at least) one night.

We then repeated selected photolysis experiments on **1a**–**1c** again in neat solvents under UV irradiation (ca. 310 nm) to ascertain the occurrence of a wavelength selective behavior of arylazo sulfonates (in analogy with arylazo sulfones).^10^ The data are collected in Table 3.

**Table 3:**
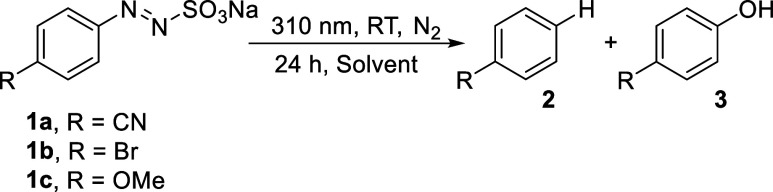
Products Distribution upon 310 nm Irradiation of Arylazo Sulfonates **1a–1c** in Different Media

solvent	**1a** ^a^		**1b** ^a^		**1c** ^a^	
	conv. (%)^b^	products yield (%)^c^	conv. (%)^b^	products yield (%)^c^	conv. (%)^b^	products yield (%)^c^
MeCN/H_2_O (4:1)	96	**2a**, 52, **3a**, <5	99	**2b**, 47, **3b**, <5	98	**2c**, 92, **3c**, <5
EtOH/H_2_O (4:1)	99	**2a**, 67, **3a**, 8	99	**2b**, 56, **3b**, 7	99	**2c**, 99, **3c**, <2
H_2_O	79	**2a**, 17, **3a**, 77	82	**2b**, 6, **3b**, 84	62	**2c**, 11, **3c**, 43

a Nitrogen equilibrated 0.05 M solution of **1a**–**c** irradiated by means of a multilamp reactor equipped with 10 × 15 W Hg phosphor-coated lamps (310 nm).

b Consumption of arylazo sulfone **1** quantified by HPLC analysis. GC yields for compounds **2a**–**c** and HPLC yields for **3a**–**c**.

c Yields are based on the arylazo sulfonate consumed.

Photolysis in the UV region induced a good conversion of **1a**–**1c** and neat water is again the medium where these compounds showed lower photoreactivity. As for the product distribution, this did not appreciably differ from that observed under visible-light irradiation except to the presence of variable amounts (<10%) of phenols **3a**–**3c** throughout the experiments carried out in mixed organic solvent/water mixtures.

Moreover, when a covered (with an aluminum foil) water solution of **1c** was heated at reflux for 48 h, partial consumption of the sulfonate (88%) was detected mainly leading to phenol **3c** (89%, based on **1c** conversion).

We next tested the ability of arylazo sulfonates in the role of arylating agents. Sulfonates **1a**–**1c** were then irradiated in the presence of furan or allylphenyl sulfone (CH_2_=CH–CH_2_SO_2_Ph, APS) in the role of nucleophiles (Table 4).

**Table 4:**
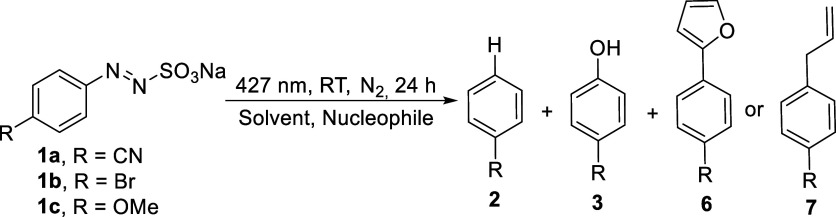
Photoreaction of **1a–1c** in the Presence of a Nucleophile

entry	1, (M)	nucleophile, (equiv)	solvent	1 (conv. %)^a^	2/3 (%)^b^	6/7 (%)^c^
1	**1a**, 0.1	**Furan**, 10	MeCN/H_2_O (4:1)	100	**2a**, 11	**6a**, 49
2	**1a**, 0.05	**Furan**, 20	H_2_O	100	**2a**, <5; **3a**, 71	**6a**, 17
3	**1b**, 0.05	**Furan**, 10	MeCN/H_2_O (4:1)	89	**2b**, 65	**6b**, 19
4	**1b**, 0.05	**Furan**, 20	MeCN/H_2_O (4:1)	94	**2b**, 8	**6b**, 67, 46^d^
5	**1b**, 0.05	**Furan**, 20	H_2_O	100	**2b**, 9; **3b**, 38	**6b**, 29
6	**1c**, 0.05	**Furan**, 10	MeCN/H_2_O (4:1)	100	**2c**, 52	**6c**, 18
7	**1c**, 0.05	**Furan**, 20	MeCN/H_2_O (4:1)	93	**2c**, 50	**6c**, 41, 34^d^
8	**1c**, 0.05	**Furan**, 20	EtOH/H_2_O (4:1)	98	**2c**, 62	**6c**, <5
9	**1c**, 0.05	**Furan**, 20	H_2_O	100	**2c**, 8; **3c**, 43	**6c**, 16
10	**1c**, 0.05	**APS**, 20	MeCN/H_2_O (4:1)	100	**2c**, 7	**7c**, 65

a Consumption of arylazo sulfone **1** quantified by HPLC analysis.

b GC-FID yield based on the consumption of **1**.

c GC-FID yield determined by using a pure sample of **6** or **7**.

d Isolated yield.

A **1a** solution irradiated in an organic/water mixture or in neat water led to biaryl **6a** in a modest yield (10% in water) even in the presence of a 20-fold excess of furan (see entries 1, 2). Again, in pure water, the main product is phenol **3a**. Slightly better arylation yields resulted in the photolysis of **1b** where in one experiment the GC yield reached 67% (along with 8% bromobenzene) and where the isolated yield of **6b** reached 46% (entry 4). Biaryl **6c** was isolated in only a 34% yield in MeCN/H_2_O 4:1 from **1c** (entry 7). The use of neat water was detrimental for the outcome of the reaction (entries 2, 5, 9) with phenols **3a**–**c** formed at the expenses of **6a**–**c**. A one shot using APS as the radical trap led to 4-allylanisole **7c** in a discrete yield (entry 10). In the latter case, neat water was not tested due to solubility problems.

The unsatisfying results obtained in the forging of C–C bonds led us to consider the use of the arylazo sulfones **1a**–**1o** as precursors for the corresponding hydrodediazosulfonyl derivatives **2a**–**2o**. To this aim, we employed a mixture of water and a good hydrogen donor (*i*PrOH or THF)^25^ as the reaction media (Table 5). As apparent from Table 5, there is not a clear advantage of the use of *i*PrOH/H_2_O 9:1 or THF/H_2_O 9:1 as the reducing media. Arenes may be formed, however, in a >90% yield depending on the sulfonate used. Thus, arenes **2g**, **2l** were formed in 92% and 99% yields in *i*PrOH/H_2_O 9:1 but compounds **2c**, **2i**, and **2o** were obtained in a >98% yield in THF/H_2_O 9:1 from the corresponding sulfonates. These data are roughly in agreement with those obtained starting from arylazo sulfones having the same substituents present on the aromatic ring of arylazo sulfonates.^25^

**Table 5:**
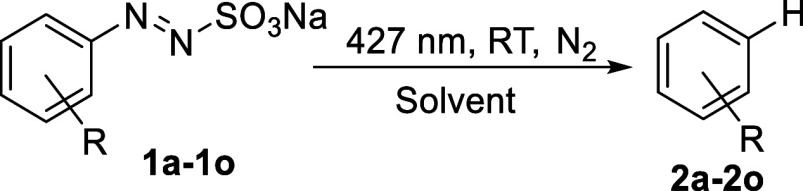
Photochemical Conversion of Sulfonates **1a–1o** into Arenes **2a–2o** ^a^

Ar–N_2_SO_3_Na	*i*PrOH/H_2_O 9:1 product (yield %)^b^	THF/H_2_O 9:1 product (yield %)^b^
**1a**, R = 4-CN	**2a**, 85	**2a**, 77
**1b**, R = 4-Br	**2b**, 62	**2b**, 73
**1c**, R = 4-MeO	**2c**, 75	**2c**, 99
**1d**, R = 4-Ac	**2d**, 79	**2d**, 64
**1e**, R = 4-NO_2_	**2e**, 58	**2e**, 51
**1f**, R = 4-Cl	**2f**, 84	**2f**, 72
**1g**, R = 4-Me	**2g**, 92	**2g**, 67
**1h**, R = 3-Br	**2h**, 47	**2h**, 76
**1i**, R = 2-Br	**2i**, 48	**2i**, 57
**1j**, R = 2-MeO	**2j**, 75	**2j**, 99
**1k**, Ar = 2-OPh	**2k**, 41	**2k**, 35
**1l**, R = 2-SMe	**2l**, 99	**2l**, 99
**1m**, R = 2,6-diMe	**2m**, 59	**2m**, 86
**1n**, R = 2,4,6-triMe	**2n**, 90	**2n**, 98
**1o**, R = 2-Cl, 4-Br	**2o**, 48	**2o**, 46

a Conditions: A 0.025 M solution of **1a**–**1o** in the chosen solvent mixture was irradiated for 2 h at 427 nm (45 W Kessil lamp) under a N_2_ atmosphere at room temperature.

b GC-FID yield determined by using a pure sample of **2a**–**2o**.

We then tested the effect of deuterated media on the photochemistry of sulfonates **1c** and **1e** (Table 6). Photolysis of the sulfonates in MeOD for 2 h at 427 nm led to a mixture of H-incorporating products **2c**,**e** (by far the main products) along with a small amount of deuterated **2c,e–d** as detected by GC/MS analysis (see Figures S3–S8). The presence of D_2_O (10% v/v) did not significantly affect the **2**/**2-d** ratio yield. On the contrary, when CD_3_OD/H_2_O (9:1) was used as the reaction media, the ratio shifted in favor of the deuterated derivatives.

**Table 6:**
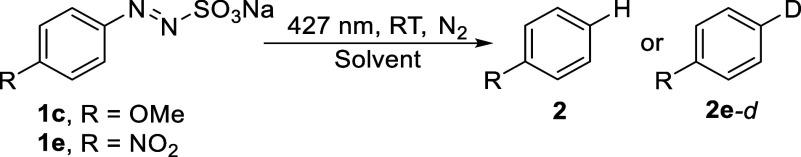
Deuteration Experiments on Sulfonates **1c** and **1e** ^a^

deuterated solvent	conv. (%)	products yield (%)^b^		conv. (%)	products yield (%)^b^	
MeOD	78	**2c**, 73	**2c–d**, 5	99	**2e**, 88	**2e–d**, 11
MeOD/D_2_O (9:1)	99	**2c**, 91	**2c–d**, 8	99	**2e**, 81	**2e–d**, 18
CD_3_OD/H_2_O (9:1)	58	**2c**, 30	**2c–d**, 28	99	**2e**, 30	**2e–d**, 69

a A 0.025 M solution of arylazo sulfonate **1c, 1e** in the chosen deuterated solvent was irradiated for 2 h at 427 nm (45 W Kessil lamp) under a N_2_ atmosphere at room temperature.

b Deuteration ratio has been quantified by GC–MS analysis (see Figures S3–S8).

Some experiments to spot the reaction intermediates involved in the process have been carried out (Scheme 2). First, irradiation of **1a** for 24 h in MeCN/H_2_O 4:1 in the presence of TEMPO (10 equiv) under deaerated conditions led to adduct **8a** in a 56% yield along with a small amount of benzonitrile **2a**, confirming that an aryl radical is released in the reaction.

**Scheme 2:**
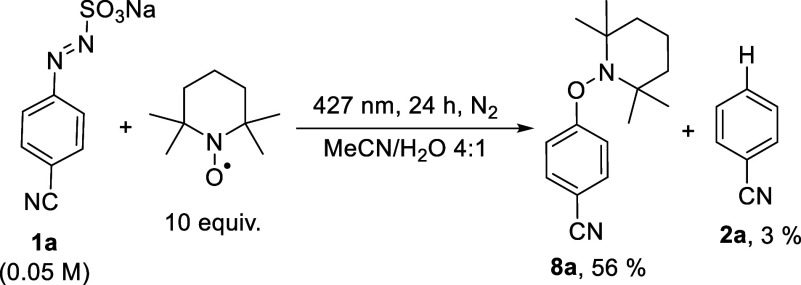
Trapping Experiments with TEMPO

To assess if a diazonium salt is released during irradiation, we performed some experiments in the presence of an excellent nucleophile such as indole. First, no EDA complexes were detected by mixing increasing amounts of indole to a solution of **1c** (Figure S11). Moreover, indole (20 equiv) was added to a solution of **1c** (0.05 M) in water and kept in the dark for one night at room temperature. No consumption of the sulfonate resulted. On the contrary the same solution irradiated for 24 h afforded adducts **9c** and **10c** in 46% and 29% yields, respectively (Scheme 3).

**Scheme 3:**
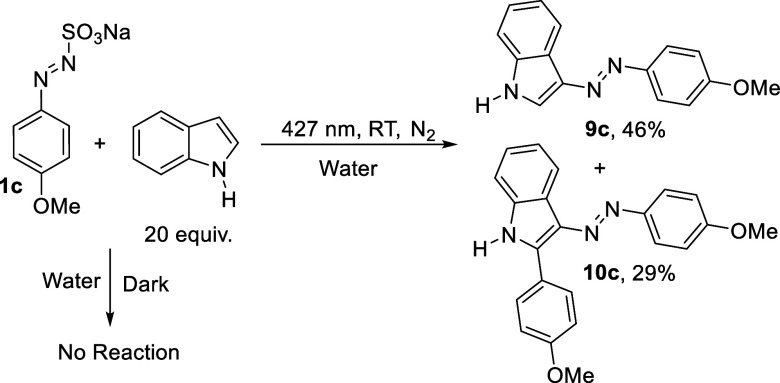
Reactivity of **1c** in the Presence of Indole

Finally, due to the solubility of compounds **1** in water we tested compound **1g** for the polymerization of a water-soluble monomer namely acrylamide. Thus, a solution of acrylamide (5 g) in water (5 mL) in the presence of **1g** (0.02 mol %) was placed in a Petri dish and irradiated at 427 nm (40 W Kessil Lamp) for 15 min, showing a marked jellification of the reaction mixture (Figure S12a,b). No polymerization was observed under dark conditions or in the absence of **1g** (Figure S12c,d).

## Discussion

Arylazo sulfonates were sparsely investigated for their possible use in synthesis.^26^ The mechanism of photodissociation of these sulfonates has been largely debated since about 60–70 years ago.^24,27–34^ Our investigation pointed out the general mechanistic scenario depicted in Scheme 4. First, arylazo sulfonates are shelf-stable compounds that did not decompose appreciably in solution (even in pure water) at room temperature when kept in the dark, and no equilibrium with a diazonium form (path *a*) occurred as demonstrated by the insensitivity to the presence of indole (Scheme 3). Arylazo sulfonates showed a good photoreactivity in solution as witnessed by the high quantum yield of decomposition (Φ_–1_ > 0.3) with water being the solvent where these compounds showed better photostability (Table 1).

**Scheme 4:**
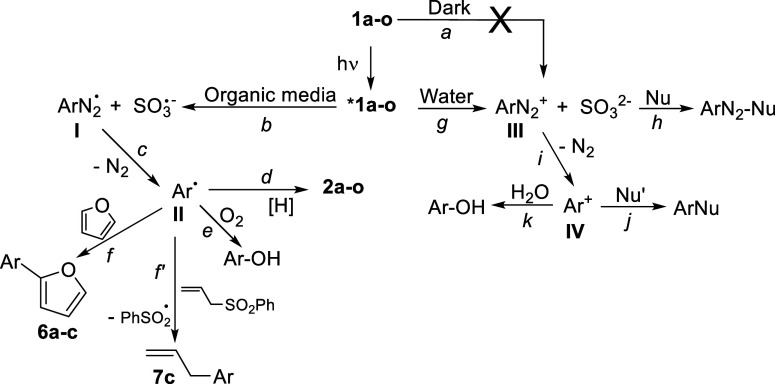
Proposed Mechanism for the Medium-Dependent Photoreactivity of Arylazo Sulfonates **1a–o**

We thought that the reaction observed may arise from the singlet excited state, in analogy with arylazo sulfones. The photochemical behavior of arylazo sulfonates depends on the media used whether organic/aqueous media or neat water. In the first case, the reactivity observed pointed to a homolytic cleavage of the N–S bond to release aryldiazenyl radical **I** and then an aryl radical **II** upon nitrogen loss (Scheme 4, paths *b*,*c*).^33^ This is confirmed by adduct **8a** formed in the presence of TEMPO (Scheme 2). In solution, the fate of the radical is hydrogen abstraction from the solvent to give the “reduced” derivative **2** (Table 2 and Scheme 4, path *d*). When the reaction was not carried out by degassing with a freeze–pump–thaw system, a small amount of phenol may be formed by the addition of the aryl radical to adventitious oxygen (path *e*).^35^

The experiments in deuterated solvents pointed out that the aryl radical may abstract a hydrogen atom either from a C–H bond (e.g., in MeOH) and, although with a lower efficiency, also from the O–H bond of water or the alcohol. The latter pathway is favored when a C–D bond is present in place of a C–H bond (e.g., in CD_3_OH). In fact, the bond dissociation energy (BDE) of the H–CH_2_OH bond is only 96 kcal mol^–1^ ^36^ and the H abstraction from a phenyl radical is largely favored especially if the aromatic ring bears an electron-withdrawing group (the BDE values of the Ar–H bond in benzene and 4-nitrobenzene are 113 and 117 kcal mol^–1^, respectively).^36^ The latter values made feasible (although not favorable) the H abstraction of the aryl radical from water (BDE of the H–OH bond = 118.8 kcal mol^–1^)^36^ but favorable from the O–H bond in MeOH (BDE = 105 kcal mol^–1^).^36^

In the presence of a π nucleophile, some arylation resulted (products **6** and **7**, Table 4) especially in non-H donating solvents such as MeCN. This can be safely attributed to the addition of the aryl radical onto furan (Scheme 4, path *f*) or allylphenyl sulfone (path *f*′). In these cases, no azo adducts were detected in the end mixture.

The scenario dramatically changed in neat water. Photolysis of **1a**–**c** in water did not lead to appreciable amounts of compound **2** but the corresponding phenols **3a**–**c** were formed instead even by deaerating the solution by the freeze–pump–thaw technique (Table 2). Accordingly, water diverts the photocleavage of compounds **1a**–**o** and prevents any aryl radical formation. Thus, a heterolytic cleavage may be alternatively envisaged to give a diazonium salt **III** (path *g*). This salt may be trapped by very nucleophilic partners such as indole to give the azoadducts **9c** and **10c** (path *h*).^37^ In the presence of less nucleophilic derivatives (e.g., furan)^38^ the azocoupling is not so favored^39^ and loss of nitrogen led to an aryl cation Ar^+^ (**IV**, path *i*). At this stage, addition to water (path *k*) or to a nucleophile (Scheme 4, path *j*) became competitive paths to give phenols and arylated products, respectively.^10^ The direct photolysis of the thus-formed diazonium salts **III** ^40^ may be safely excluded since the latter compounds did not absorb the visible photons used in the reaction. However, degradation to diazonium salt (albeit inefficiently) may be likewise induced by prolonged heating in water at 100 °C.

At any rate, contrary to arylazo sulfones, a wavelength behavior of arylazo sulfonates did not operate in this case since the product distribution observed under visible (427 nm) or UV (ca. 310 nm) light irradiation was roughly the same.

## Conclusions

In conclusion, we explored the medium-dependent photoreactivity of arylazo sulfonates, shelf-stable compounds having a −N_2_SO_3_Na group as a dyedauxiliary moiety. This is one of the rare examples where an aryl radical is formed in a (mixed) aqueous mixture with no need of biphasic systems or surfactants.^41^ Photoinduced ionic and radical decomposition competes depending on the media used. When the media was made mainly of an organic solvent (e.g., MeCN, an alcohol, etc.), photohomolysis of the N–S bond occurred, thus releasing an aryl radical prone to be used in arylation reactions. As for the latter point, no advantage on the substitution of −N_2_SO_2_R for a −N_2_SO_3_Na group resulted.^10^

Interestingly, the peculiar structure of these sulfonates allows their solubility in neat water. Here the radical chemistry is prevented by an efficient photoheterolysis that releases the corresponding diazonium salt prone to being added to a strong nucleophile (maintaining the nitrogen atoms) or decomposed into an aryl cation. In the latter case, competition of water with the nucleophile strongly affected the arylation yield, and phenol is preferentially formed.

Finally, the great solubility of compounds **1** in water may make them ideal photoinitiators for free-radical polymerizations.

## Experimental Section

### General Information

^1^H and ^13^C NMR spectra were recorded on 300 and 75 MHz spectrometers, respectively. The attributions were made on the basis of ^1^H and ^13^C NMR experiments; chemical shifts are reported in parts per million downfield from TMS. GC analyses were performed using an HP SERIES 5890 II equipped with a fire ion detector (FID, temperature 350 °C). Analytes were separated using a Restek Rtx-5MS (30 m × 0.25 mm × 0.25 μm) capillary column with nitrogen as a carrier gas at 1 mL min^–1^. The injector temperature was 250 °C. The GC oven temperature was held at 80 °C for 2 min, increased to 250 °C by a temperature ramp of 10 °C min^–1^, and held at this temperature for 10 min. HPLC analyses have been performed by means of a JASCO instrument equipped with two PU980 pumps and a UV975 detector (Thermo Fisher ODS-Hypersyl column). Anilines **S1a-o**, furan, allyltrimethylsilane, allyl phenyl sulfone, sodium sulfite, and solvents (HPLC grade) were commercially available and used as received.

### General Procedure for the Synthesis of Arylazo Sulfonates **1a–o**

Compounds **1a**–**o** were obtained by a procedure previously reported.^42^ The chosen aniline (**S1a-o**, 10.0 mmol) was dissolved in HCl 1.5 M (20 mL) in a reactor flask covered with an aluminum sheet. After cooling to 0 °C, a solution of sodium nitrite (10.0 mmol, 1.00 equiv) in water (5 mL) was added over a period of approximately 10 min under stirring. The resulting mixture was then stirred at 0 °C for 20 min. A solution of sodium carbonate (6.0 mmol, 0.60 equiv) dissolved in water (5 mL) was thus added to the so formed suspension of arenediazonium chlorides to reach a pH = ca. 7.5. A cold solution of sodium sulfite (10.0 mmol, 1.00 equiv) in water (5 mL) was rapidly added, and the resulting mixture was stirred for additional 15 min at 0 °C. The suspension was then heated to 30 °C by a heating mantle for 3 h, then filtered, and the liquid phase concentrated in vacuo until incipient crystallization. The solution was kept in a refrigerator for 16 h. The obtained colored solids (**1a**–**o**) were filtered under vacuum, washed with methyl-*tert*-butyl ether (MTBE 50 mL), and dried in vacuo.

Caution! Anilines may be carcinogenic chemicals, constitute significant safety hazards, and must be handled with extreme care.

Caution! Sodium nitrite is a very toxic compound and must be handled with extreme care.

#### Sodium 2-(4-Cyanophenyl)­diazene-1-sulfonate (**1a**)

Compound **1a** was obtained from 4-aminobenzonitrile (**S1a**, 1.181 g, 10 mmol) in a 63% yield (orange solid, 1.45 g). Spectroscopic data of **1a** are in accordance with the literature.^26b^ **1a**. ^1^H NMR (300 MHz, DMSO-*d*_6_): δ 8.12–8.01 (m, 2H), 7.92–7.82 (m, 2H). ^13^C­{^1^H}­NMR (75 MHz, DMSO-*d*_6_): δ 152.7, 133.9, 123.1, 118.2, 113.8. mp (dec) = 211 °C. HRMS (ESI) *m*/*z*: product unstable during the analysis.

#### Sodium 2-(4-Bromophenyl)­diazene-1-sulfonate (**1b**)

Compound **1b** was obtained from 4-bromoaniline (**S1b**, 1.72 g, 9.99 mmol) in a 63% yield (yellow solid, 1.8 g). Spectroscopic data of **1b** are in accordance with the literature.^42^ **1b**. ^1^H NMR (300 MHz, DMSO-*d*_6_): δ 7.83–7.74 (m, 2H), 7.72–7.64 (m, 2H). ^13^C­{^1^H}­NMR (75 MHz, DMSO-*d*_6_): δ 149.3, 132.6, 125.3, 124.4. mp (dec) = 213 °C. HRMS (ESI): product unstable during the analysis.

#### Sodium 2-(4-Methoxyphenyl)­diazene-1-sulfonate (**1c**)

Compound **1c** was obtained from 4-anisidine (**S1c**, 1.23 g, 10.0 mmol) in an 86% yield (yellow solid, 2.06 g). Spectroscopic data of **1c** are in accordance with the literature.^26b^ **1c**. ^1^H NMR (300 MHz, DMSO-*d*_6_): δ 7.82–7.69 (m, 2H), 7.17–7.05 (m, 2H), 3.85 (s, 3H). ^13^C­{^1^H}­NMR (75 MHz, DMSO-*d*_6_): δ 162.3, 144.1, 124.6, 114.6, 55.6. mp (dec) = 201 °C. HRMS (ESI): product unstable during the analysis. ^1^H NMR spectroscopy was used to determine the purity of the compound (>99%, 1,3,5-trimethoxybenzene as the internal standard).

#### Sodium 2-(4-Acetylphenyl)­diazene-1-sulfonate (**1d**)

Compound **1d** was obtained from 4-aminoacetophenone (**S1d**, 1.35 g, 9.97 mmol) in a 61% yield (orange solid, 1.53 g). Spectroscopic data of **1d** are in accordance with the literature.^42^ **1d**. ^1^H NMR (300 MHz, DMSO-*d*_6_): δ 8.18–8.11 (m, 2H), 7.89–7.79 (m, 2H), 2.64 (s, 3H). ^13^C­{^1^H}­NMR (75 MHz, DMSO-*d*_6_): δ 197.4, 152.9, 138.8, 129.6, 122.5, 27.0. mp (dec) = 190 °C. HRMS (ESI): product unstable during the analysis.

#### Sodium 2-(4-Nitrophenyl)­diazene-1-sulfonate (**1e**)

Compound **1e** was obtained from 4-nitroaniline (**S1e**, 1.38 g, 9.99 mmol) in a 78% yield (yellow solid, 1.97 g). Spectroscopic data of **1e** are in accordance with the literature.^42^ **1e**. ^1^H NMR (300 MHz, DMSO-*d*_6_): δ 8.49–8.32 (m, 2H), 8.02–7.87 (m, 2H). ^13^C­{^1^H}­NMR (75 MHz, DMSO-*d*_6_): δ 153.9, 148.9, 125.1, 123.4. mp (dec) = 186 °C. HRMS (ESI): product unstable during the analysis.

#### Sodium 2-(4-Chlorophenyl)­diazene-1-sulfonate (**1f**)

Compound **1f** was obtained from 4-cloroaniline (**S1f**, 1.274 g, 9.99 mmol) in a 69% yield (yellow solid, 1.53 g). Spectroscopic data of **1f** are in accordance with the literature.^26b^ **1f**. ^1^H NMR (300 MHz, DMSO-*d*_6_): δ 7.82–7.73 (m, 2H), 7.70–7.60 (m, 2H). ^13^C­{^1^H}­NMR (75 MHz, DMSO-*d*_6_): δ 149.0, 136.5, 129.6, 124.3. mp (dec) = 206 °C. HRMS (ESI): product unstable during the analysis.

#### Sodium 2-(4-Methylphenyl)­diazene-1-sulfonate (**1g**)

Compound **1g** was obtained from 4-toluidine (**S1g**, 1.05 g, 9.80 mmol) in a 91% yield (yellow solid, 1.96 g). **1g**. ^1^H NMR (300 MHz, DMSO-*d*_6_): δ 7.73–7.57 (m, 2H), 7.41–7.34 (m, 2H), 2.39 (s, 3H). ^13^C­{^1^H}­NMR (75 MHz, DMSO-*d*_6_): δ 148.3, 142.1, 130.0, 122.5, 21.0. mp (dec) = 198 °C. HRMS (ESI): product unstable during the analysis.

#### Sodium 2-(3-Bromophenyl)­diazene-1-sulfonate (**1h**)

Compound **1h** was obtained from 3-bromoaniline (**S1h**, 1.71 g, 9.92 mmol) in a 57% yield (orange solid, 1.97 g). **1h**. ^1^H NMR (300 MHz, DMSO-*d*_6_): δ 7.82 (t, *J* = 2.0 Hz, 1H), 7.77 (dt, *J* = 8.0, 2.0 Hz, 2H), 7.54 (t, *J* = 8.0 Hz, 1H). ^13^C­{^1^H}­NMR (75 MHz, DMSO-*d*_6_): δ 151.5, 134.4, 131.7, 124.0, 122.7, 122.5. mp (dec) = 175 °C. HRMS (ESI): product unstable during the analysis.

#### Sodium 2-(3-Methoxyphenyl)­diazene-1-sulfonate (**1i**)

Compound **1i** was obtained from 3-anisidine (**S1i**, 1.23 g, 10.0 mmol) in a 90% yield (yellow solid, 1.56 g). **1i**. ^1^H NMR (300 MHz, DMSO-*d*_6_): δ 7.53–7.46 (m, 1H), 7.41–7.36 (m, 1H), 7.27–7.24 (m, 1H), 7.17–7.12 (m, 1H), 3.83 (s, 3H). ^13^C­{^1^H}­NMR (75 MHz, DMSO-*d*_6_): δ 160.0, 151.6, 130.3, 118.3, 116.3, 105.8, 55.4. mp (dec) = 164 °C. HRMS (ESI): product unstable during the analysis.

#### Sodium 2-(2-Bromophenyl)­diazene-1-sulfonate (**1j**)

Compound **1j** was obtained from 2-bromoaniline (**S1j**, 1.672 g, 9.72 mmol) in a 77% yield (orange solid, 2.14 g). **1j**. ^1^H NMR (300 MHz, DMSO-*d*_6_): δ 7.89–7.79 (m, 1H), 7.53–7.46 (m, 2H), 7.43–7.35 (m, 1H). ^13^C­{^1^H}­NMR (75 MHz, DMSO-*d*_6_): δ 148.0, 133.8, 133.0, 128.8, 123.7, 118.0. mp (dec) = 217 °C. HRMS (ESI): product unstable during the analysis.

#### Sodium 2-(2-Phenoxyphenyl)­diazene-1-sulfonate (**1k**)

Compound **1k** was obtained from 2-phenoxyaniline (**S1k**, 1.85 g, 10.00 mmol) in a 94% yield (yellow solid, 2.8 g). **1k**. ^1^H NMR (300 MHz, DMSO-*d*_6_): δ 7.57–7.45 (m, 2H), 7.46–7.37 (m, 2H), 7.29–7.13 (m, 2H), 7.13–6.96 (m, 3H). ^13^C­{^1^H}­NMR (75 MHz, DMSO-*d*_6_): δ 156.5, 154.5, 141.5, 133.4, 130.2, 123.9, 123.8, 119.7, 119.1, 117.3. mp (dec) = 86 °C. HRMS (ESI): product unstable during the analysis.

#### Sodium 2-(2-Methylthiophenyl)­diazene-1-sulfonate (**1l**)

Compound **1l** was obtained from 2-methylthioaniline (**S1l**, 1.23 g, 10.00 mmol) in a 99% yield (orange solid, 3.00 g). **1l**.^1^H NMR (300 MHz, DMSO-*d*_6_): δ 7.55–7.47 (m, 1H), 7.46–7.37 (m, 2H), 7.28–7.19 (m, 1H), 2.46 (s, 3H). ^13^C­{^1^H}­NMR (75 MHz, DMSO-*d*_6_): δ 146.6, 140.1, 132.2, 125.3, 124.6, 117.3, 14.1. mp (dec) = 59 °C. HRMS (ESI): product unstable during the analysis.

#### Sodium 2-(2,6-Dimethylphenyl)­diazene-1-sulfonate (**1m**)

Compound **1m** was obtained from 2,6-dimethylaniline (**S1m**, 1.23 g, 10.00 mmol) in a 78% yield (yellow solid, 1.83 g). **1m**. ^1^H NMR (300 MHz, DMSO-*d*_6_): δ 7.20–7.09 (m, 3H), 2.14 (s, 6H). ^13^C­{^1^H}­NMR (75 MHz, DMSO-*d*_6_): δ 149.6, 129.0, 128.8, 128.0, 17.6. mp (dec) = 189 °C. HRMS (ESI): product unstable during the analysis.

#### Sodium 2-(2,4,6-Trimethylphenyl)­diazene-1-sulfonate (**1n**)

Compound **1n** was obtained from 2,4,6-trimethylaniline (**S1n**, 1.4 mL, 10.00 mmol) in a 57% yield (yellow solid, 1.43 g). **1n**. ^1^H NMR (300 MHz, DMSO-*d*_6_): δ 6.94 (s, 2H), 2.26 (s, 3H), 2.16 (s, 6H). ^13^C­{^1^H}­NMR (75 MHz, DMSO-*d*_6_): δ 146.8, 137.8, 129.9, 129.6, 20.6, 18.0. mp (dec) = 208 °C. HRMS (ESI): product unstable during the analysis.

#### Sodium 2-(2-Chloro-4-bromophenyl)­diazene-1-sulfonate (**1o**)

Compound **1o** was obtained from 2-chloro-4-bromoaniline (**S1o**, 2.06 g, 10.00 mmol) in a 94% yield (yellow solid, 2.71 g). **1o**. ^1^H NMR (300 MHz, DMSO-*d*_6_): δ 8.03–7.98 (m, 1H), 7.71–7.65 (m, 1H), 7.40–7.35 (m, 1H). ^13^C­{^1^H}­NMR (75 MHz, DMSO-*d*_6_): δ 145.9, 134.8, 133.0, 131.4, 125.4, 119.3. mp (dec) = 153 °C. HRMS (ESI): product unstable during the analysis.

### General Procedure for the Irradiation of **1** in Neat Solvents

In a dried Pyrex (or quartz) vessel, arylazo sulfonate **1** (0.05 mmol) was dissolved in the chosen solvent mixture (1 mL). An inert atmosphere was settled up by purging nitrogen into the solution for 3 min and then the vial was capped. The solution was then irradiated with a 427 nm Kessil Lamp (Figure S9a) (or 310 nm phosphor-coated lamps) at room temperature in a photochemical reactor for 24 h.

The consumption of **1** was quantified by HPLC analysis. Conversion to arenes **2** has been evaluated by GC-FID analysis and quantified via calibration curves with internal standard (dodecane 0.5 μL/mL); phenols **3** in the photolyzed solution were quantified by HPLC calibration curve analysis.

Caution! Ultraviolet light is damaging to biological tissues. Caution is required when working with the lamp and protective eyewear must be used. In some cases, the solution was deaerated with a freeze–pump–thaw method. Caution! Extreme care should be taken both in the handling of the cryogen liquid nitrogen and its use in the Schlenk line trap to avoid condensation of oxygen from air.

### General Procedure for Photochemical Hydrodeamination and Deuteration of **1**

In a dried Pyrex vessel, arylazo sulfonate **1** (0.025 mmol) was dissolved in the chosen solvent mixture (1 mL). An inert atmosphere was settled up by purging nitrogen into the solution for 3 min, and then the vial was capped. The solution was then irradiated with a 427 nm Kessil Lamp at room temperature in a photochemical reactor for 2 h (Figure S9a). Conversion to **2** has been evaluated by GC-FID analysis and quantified via calibration curves with an internal standard (dodecane, 0.5 μL/mL). Deuteration ratio has been quantified by GC–MS analysis by comparison of the intensity of the appropriate mass picks and then normalized (see Figures S3–S8).

### Irradiation of Arylazo Sulfonates in the Presence of Nucleophiles

In a dried glass vessel, arylazo sulfonate **1** (0.01–0.1 mmol) was dissolved in the chosen solvent mixture (1 mL). An inert atmosphere was settled up by purging nitrogen into the solution for 3 min, then the nucleophile (10–20 equiv) was added, and the vessel was capped. The solution was then irradiated with a 427 nm Kessil Lamp at room temperature in a photochemical reactor for 24 h (Figure S9b).

Consumption of **1** was quantified by HPLC analysis comparing chromatographic areas before and after irradiation. Conversion to derivates **2**, **6**, and **7** has been evaluated by GC-FID analysis and quantified via calibration curves with internal standard (dodecane, 0.5 μL/mL).

In selected cases, reactions have been performed on a 0.05–0.5 mmol scale to isolate and characterize the products. After irradiation, solvent was removed and products **6, 9**, or **10** purified by silica gel flash column chromatography (eluent mixture CyHex/EA).

#### 2-(4-Bromophenyl)-furan (**6b**)

From 71.9 mg (0.25 mmol, 0.05 M) of **1b** and 365 μL (5.0 mmol, 20 equiv) of furan in 5 mL of mixture MeCN/H_2_O 4:1. Purification by silica gel flash column chromatography (eluant mixture, CyHex/EA from 99:1 to 9:1) afforded product **6b** in a 46% yield (23.4 mg, pale yellow, viscous oil). Spectroscopic data are in accordance with literature.^43^ **6b**. ^1^H NMR (300 MHz, chloroform-*d*): δ 7.66–7.42 (m, 5H), 6.71–6.44 (m, 2H). ^13^C­{^1^H}­NMR (75 MHz, chloroform-*d*): δ 152.9, 142.3, 131.7, 129.7, 125.2, 120.9, 111.7, 105.4.

#### 2-(4-Methoxyphenyl)-furan (**6c**)

From 59.5 mg (0.25 mmol, 0.05 M) of **1c** and 365 μL (5.0 mmol, 20 equiv) of furan in 5 mL of mixture MeCN/H_2_O 4:1. Purification by silica gel flash column chromatography (eluent mixture, CyHex/EA from 99:1 to 8:2) afforded product **6c** in a 34% yield (14.8 mg, colorless viscous oil). Spectroscopic data are in accordance with literature.^10^ **6c**. ^1^H NMR (300 MHz, chloroform-*d*): δ 7.66–7.60 (m, 2H), 7.45 (dd, *J* = 1.8, 0.8 Hz, 1H), 6.98–6.92 (m, 2H), 6.54 (dd, *J* = 3.3, 0.8 Hz, 1H), 6.47 (dd, *J* = 3.3, 1.8 Hz, 1H), 3.86 (s, 3H). ^13^C­{^1^H}­NMR (75 MHz, chloroform-*d*): δ 159.0, 154.0, 141.4, 125.2, 124.0, 114.1, 111.5, 103.4, 55.3.

#### 3-((4-Methoxyphenyl)­diazenyl)-1*H*-indole (**9c**)

From 59.5 mg (0.25 mmol, 0.05 M) of **1c** and 586 mg (5.0 mmol, 20 equiv) of indole in 5 mL of H_2_O. Purification by silica gel flash column chromatography (eluant mixture, CyHex/EA from 99:1 to 80:10) afforded product **9c** in a 46% yield (29.1 mg, yellow oil). **9c**. ^1^H NMR (300 MHz, chloroform-*d*): δ 8.66–8.51 (m, 2H), 7.97 (d, *J* = 2.9 Hz, 1H), 7.91–7.84 (m, 2H), 7.42–7.37 (m, 1H), 7.33–7.28 (m, 2H), 7.03–6.98 (m, 2H), 3.89 (s, 3H). ^13^C­{^1^H}­NMR (75 MHz, chloroform-*d*): δ 160.8, 148.0, 136.9, 136.4, 129.4, 124.3, 123.5, 123.2, 122.9, 119.3, 114.3, 111.4, 55.7. HRMS (ESI) *m*/*z*: calcd for C_15_H_15_N_3_O [M + H]^+^: 252.1131; found, 252.1126.

#### 2-(4-Methoxyphenyl)-3-((4-methoxyphenyl)­diazenyl)-1H-indole (**10c**)

From 59.5 mg (0.25 mmol, 0.05 M) of **1c** and 586 mg (5.0 mmol, 20 equiv) of indole in 5 mL of H_2_O. Purification by silica gel flash column chromatography (eluant mixture, CyHex/EA from 99:1 to 85:15) afforded product **10c** in a 29% yield (26 mg, yellow oil). **10c**. ^1^H NMR (300 MHz, chloroform-*d*): δ 8.71–8.59 (m, 1H), 8.48 (s, 1H), 7.98 (d, *J* = 8.8 Hz, 2H), 7.87 (d, *J* = 8.9 Hz, 2H), 7.42–7.36 (m, 1H), 7.32–7.27 (m, 2H), 7.07 (d, *J* = 8.8 Hz, 2H), 7.01 (d, *J* = 8.9 Hz, 2H), 3.93–3.86 (m, 6H). ^13^C­{^1^H}­NMR (75 MHz, chloroform-*d*): δ 160.6, 160.5, 148.8, 135.5, 132.0, 130.6, 124.3, 123.8, 123.7, 123.1, 120.5, 114.5, 114.3, 110.8, 55.7, 55.6. HRMS (ESI) *m*/*z*: calcd for C_22_H_20_N_3_O_2_ [M + H]^+^: 358.1550; found, 358.1543.

### General Procedure for the Photopolymerization Tests

A solution of acrylamide (5 g) in water (5 mL) in the presence of **1g** (0.02 mol %) was placed in a Petri dish (Figure S12a) and irradiated at 427 nm (40 W Kessil Lamp) for 15 min.

Photolysis induced a marked jellification of the reaction mixture (Figure S12b).

Caution! Acrylamide may be a carcinogenic chemical and may constitute significant safety hazards and must be handled with extreme care.

## Supplementary Material



## Data Availability

The data underlying this study are available in the published article and its Supporting Information.

## Notes

^1^H and ^13^C NMR spectra for all new compounds and additional details on experiments including photographs of the experimental setup (PDF)

## References

[1] b Hartonen, K. ; Riekkola, M.-L. . In The Application of Green Solvents in Separation Processes; Pena-Pereira, F. ; Tobiszewski, M. , Eds.; Elsevier, 2017; pp 19–55.

[2] Narayan S., Muldoon J., Finn M. G., Fokin V. V., Kolb H. C., Sharpless K. B. (2005). “On Water”: Unique Reactivity of Organic
Compounds in Aqueous Suspension. Angew. Chem.,
Int. Ed..

[3] c Lindstrom, U. M. Organic Reactions in Water; Blackwell: Oxford, 2007.

[4] Blackmond D. G., Armstrong A., Coombe V., Wells A. (2007). Water in Organocatalytic
Processes: Debunking the Myths. Angew. Chem.,
Int. Ed..

[5] Zuo Y.-J., Qu J. (2014). How Does Aqueous Solubility
of Organic Reactant Affect a Water-Promoted
Reaction?. J. Org. Chem..

[6] Russo C., Brunelli F., Tron G. C., Giustiniano M. (2023). Visible-Light
Photoredox Catalysis in Water. J. Org. Chem..

[7] a Modern Photocatalytic Strategies in Natural Product Synthesis; Kinghorn, A. D. ; Falk, H. ; Gibbons, S. ; Asakawa, Y. ; Liu, J.-K. ; Dirsch, V. M. , Eds. ; Springer Nature: Switzerland AG, 2023; Vol. 120.

[8] Di
Terlizzi L., Nicchio L., Protti S., Fagnoni M. (2024). Visible Photon
as the Ideal Reagent for the Activation of Coloured Organic Compounds. Chem. Soc. Rev..

[9] Qiu D., Lian C., Mao J., Fagnoni M., Protti S. (2020). Dyedauxiliary
groups, an emerging approach in organic
chemistry. The case of arylazo sulfones. J.
Org. Chem..

[10] Crespi S., Protti S., Fagnoni M. (2016). Wavelength
Selective Generation of
Aryl Radicals and Aryl Cations for Metal-free Photoarylations. J. Org. Chem..

[11] Liu Q., Liu F., Yue H., Zhao X., Li J., Wei W. (2019). Photocatalyst free
visible light-induced synthesis of β-oxo sulfones via oxysulfonylation
of alkenes with arylazo sulfones and dioxygen in air. Adv. Synth. Catal..

[12] Nitti A., Martinelli A., Batteux F., Protti S., Fagnoni M., Pasini D. (2021). Blue Light Driven Free-Radical Polymerization
using
Arylazo Sulfones as Initiators. Polym. Chem..

[13] Di Terlizzi L., Martinelli A., Merli D., Protti S., Fagnoni M. (2023). Arylazo Sulfones as
Non-Ionic Visible-Light Photoacid Generators. J. Org. Chem..

[14] Schmitt R., Glutz L. (1869). Ueber Diazophenole. Ber. Dtsch. Chem. Ges..

[15] Dunkin I. R., Gittinger A., Sherrington D. C., Whittaker P. (1994). A Photodestructible Surfactant. J. Chem. Soc., Chem. Commun..

[16] Nuyken O., Knepper T., Voit B. (1989). Sulfur-containing
azoinitiators and their properties. Makromol.
Chem..

[17] b Perchyonok, V. T. Radical Reactions in Aqueous Media. RSC Green Chemistry Series No. 6; The Royal Society of Chemistry: Cambridge, 2010.

[18] Yorimitsu H., Nakamura T., Shinokubo H., Oshima K., Omoto K., Fujimoto H. (2000). Powerful Solvent Effect of Water in Radical Reaction:
Triethylborane-Induced Atom-Transfer Radical Cyclization in Water. J. Am. Chem. Soc..

[19] Wetzel A., Pratsch G., Kolb R., Heinrich M. R. (2010). Radical Arylation of Phenols, Phenyl Ethers, and Furans. Chem.—Eur. J..

[20] Liu W., Yang X., Gao Y., Li C.-J. (2017). Simple and Efficient
Generation of Aryl Radicals from Aryl Triflates: Synthesis of Aryl
Boronates and Aryl Iodides at Room Temperature. J. Am. Chem. Soc..

[21] Garden S. J., Avila D. V., Beckwith A. L. J., Bowry V. W., Ingold K. U., Lusztyk J. (1996). Absolute Rate Constant
for the Reaction of Aryl Radicals with Tri-*n*-butyltin
Hydride. J. Org. Chem..

[22] Mardyukov A., Sanchez-Garcia E., Crespo-Otero R., Sander W. (2009). Interaction and Reaction
of the Phenyl Radical with Water: A Source of OH Radicals. Angew. Chem., Int. Ed..

[23] Di
Terlizzi L., Nicchio L., Callegari C., Scaringi S., Neuville L., Fagnoni M., Protti S., Masson G. (2023). Visible-light-mediated Divergent and Regioselective
Vicinal Difunctionalization of Styrenes with Arylazo Sulfones. Org. Lett..

[24] de
Jonge J., Dijkstra R. (1956). Some photochemical properties of
alkali salts of aryldiazosulphonic acids. Recl.
Trav. Chim. Pays-Bas.

[25] Amin H. I. M., Raviola C., Amin A. A., Mannucci B., Protti S., Fagnoni M. (2019). Hydro/deutero Deamination
of Arylazo Sulfones Under
Metal- and (Photo)­Catalyst-Free Conditions. Molecules.

[26] Rapta P., Staško A., Bustin D., Nuyken O., Voit B. (1992). Electrochemical reduction
of azo sulfonates and sulfones. A cyclic voltammetry and EPR study. J. Chem. Soc., Perkin Trans..

[27] Freeman H. C., Le Fèvre R. J.
W. (1951). Hantzsch’s isomeric
diazosulphonates. J. Chem. Soc..

[28] Lewis E. S., Suhr H. (1959). Untersuchungen über die Reaktion von Diazoniumsalzen mit Sulfit. Chem. Ber..

[29] Van
Der Veen J., Helfferich J., Van Beek L. K. H. (1966). Photo isomerization
of methoxybenzene diazosulfonates. Recl. Trav.
Chim. Pays-Bas.

[30] van
Beek L. K. H., Hellferich J., Jonker H., Thijssens T. P. G. W. (1967). Properties
of diazosulfonates. Part I: The dissociation of methoxybenzenediazosulfonates. Recl. Trav. Chim. Pays-Bas.

[31] Jonker H., Thijssens T. P. G. W., Van Beek L. K. H. (1968). Properties of
diazosulfonates. Part
VI. Quantum yields for the photolysis of 2-methoxybenzenediazonium
and for the photo-isomerization of 2-methoxybenzene-trans-diazosulfonate. Recl. Trav. Chim. Pays-Bas.

[32] Franzke D., Voit B., Nuyken O., Wokaun A. (1992). Wavelength-dependent
photolysis of 3-vinyl-phenyl-azosulfonate. J.
Photochem. Photobiol., A.

[33] Franzke D., Voit B., Nuyken O., Wokaun A. (1992). Pulsed ultraviolet
laser photolysis of substituted phenyl azosulfonates: Wavelength dependent
effects. Mol. Phys..

[34] Staško A., Szaboova K., Cholvad V., Nuyken O., Dauth J. (1993). The photochemical
decomposition of azo compounds (a spin trap study). J. Photochem. Photobiol., A.

[35] Chawla R., Jaiswal S., Dutta P. K., Yadav L. D. S. (2021). A photocatalyst-free
visible-light-mediated solvent-switchable route to stilbenes/vinyl
sulfones from β-nitrostyrenes and arylazo sulfones. Org. Biomol. Chem..

[36] Luo, Y.-R. Handbook of Bond Dissociation Energies in Organic Compounds; CRC Press: Boca Raton, FL, 2003.

[37] Daly S., Hayden K., Malik I., Porch N., Tang H., Rogelj S., Frolova L. V., Lepthien K., Kornienko A., Magedov I. V. (2011). Bioorg. Unprecedented
C-2 arylation of indole with diazonium salts: Syntheses of 2,3-disubstituted
indoles and their antimicrobial activity. Med.
Chem. Lett..

[38] Mayr H., Kempf B., Ofial A. R. (2003). π-Nucleophilicity
in Carbon-Carbon
Bond-Forming Reactions. Acc. Chem. Res..

[39] Shaaban S., Jolit A., Petkova D., Maulide N. (2015). A family of low molecular-weight,
organic catalysts for reductive C–C bond formation. Chem. Commun..

[40] Milanesi S., Fagnoni M., Albini A. (2003). Cationic arylation
through photo­(sensitised)
decomposition of diazonium salts. Chemoselectivity of triplet phenyl
cations. Chem. Commun..

[41] Das A., Justin Thomas K. R. (2024). Generation
and Application of Aryl Radicals Under Photoinduced Conditions. Chem.—Eur. J..

[42] Nicchio L., Médard J., Decorse P., Gam-Derouich S., Chevillot-Biraud A., Luo Y., Mangeney C., Berisha A., Averseng F., Fagnoni M., Protti S., Pinson J. (2023). Selective
N_sp2_- and C_sp2_- photografting of Au-Surface
by aryldiazonium salts and arylazo sulfonates. Chem.—Eur. J..

[43] Ding R., Liu Q., Zheng L. (2023). Piezoelectric Metal-Organic Frameworks Mediated Mechanoredox
Borylation and Arylation Reactions by Ball Milling. Chem.—Eur. J..

