# Supplementary material for: Water Effect on the Photochemistry of Arylazo Sulfonates
Source: J Org Chem. 2025 Apr 30;90(20):6726–36. doi: 10.1021/acs.joc.5c00314 (PMC12117561; doi:10.1021/acs.joc.5c00314)

Water Effect on the Photochemistry of Arylazo Sulfonates

Luca Nicchio,[a,b] Hawraz Ibrahim M. Amin,[a,c] Stefano Genualdo,[a]
Stefano Protti,[a] Maurizio Fagnoni[a]*

[a] *PhotoGreen Lab, Department of Chemistry, University of Pavia, Viale Taramelli 12, 27100 Pavia, Italy*

[b] *Institut de Chimie des Substances Naturelles (ICSN), CNRS UPR 2301, Université Paris-Saclay, 1 avenue de la Terrasse, 91198Gif-sur-Yvette Cedex, France*

[c] *Department of Chemistry, College of Science, Salahaddin University-Erbil, 44001 Erbil, Iraq*

Email: maurizio.fagnoni@unipv.it

TABLE OF CONTENTS

1. Experimental details **S3**

1.1 General Information **S3**

1.2 Photophysical data for arylazo sulfonates 1a-o **S5**

1.3 Photochemical experiments **S9**

1.4 Mechanistic experiments **S17**

1.5 Thermal behaviour of 1c **S20**

1.6 Free Radical Polymerization (FRP) experiment **S20**

2. References **S20**

3. Copy of the NMR spectra **S21**

1 Experimental Details.

1.1 General Information.

Anilines **S1a-o**, furan, allyltrimethylsilane, allyl phenyl sulfone, sodium sulfite and solvents (HPLC grade) were commercially available and used as received. ***Caution!*** *Anilines may be carcinogenic chemicals and may constitute significant safety hazards and must be handled with extreme care.* Compounds **6** have been synthesized as references following reported procedures.[S1,S2] Analytical thin layer chromatography (TLC) plates (silica gel 60 F254) were monitored with a UV lamp (254 nm). Column chromatography was carried out on silica gel (average particle size 60 μm) by using automatic LC system. UV–Vis spectra were recorded on a V-550 Jasco spectrophotometer. Fluorescence spectra were performed using a LS-55 Perkin Elmer spectrofluorometer.

^{1}H NMR spectra were recorded on a Bruker Avance 300 and proton-decoupled carbon ^{13}C{1H} NMR spectra were recorded at 75 MHz. The attributions were made on the basis of NMR experiments; chemical shifts are reported in parts per million (ppm) downfield from TMS. The following abbreviations are used for the multiplicities: s = singlet, d = doublet, t = triplet, q = quadruplet, m = multiplet. Coupling constants (*J*) are reported in Hertz (Hz). High resolution mass spectra were determined on an X500B QTOF System (SCIEX, Framingham, MA 01701 USA) available at the Centro Grandi Strumenti (CGS) at the University of Pavia, equipped with the Twin Sprayer ESI probe and coupled to an ExionLC™ system (SCIEX). GC/MS analyses were carried out on a Thermo Scientific DSQII single quadrupole GC/MS system (TraceDSQII mass spectrometer, Trace GC Ultra gas chromatograph, TriPlus autosampler - ThermoFisher Scientific, Waltham, MA, USA), available at the Centro Grandi Strumenti (CGS) at the University of Pavia. Chromatography was performed on a Rxi-5ms capillary column (30 m length×0.25 mm ID×0.25 μm film thickness, Restek, Milan, Italy) with Helium (>99.99 %) as carrier gas at a constant flow-rate of 1.0 mL/min. An injection volume of 1 μL was employed. The injector temperature was set at 250 °C and it was operated in split mode, with a split flow of 10 mL/min. The oven temperature was programmed from 80 °C (isothermal for 5 min) to 120 °C at the rate of 3 °C/min, then from 120 °C to 250 °C (isothermal for 5 min) at the rate of 10 °C/min. Mass transfer line temperature was set at 270 °C. Total GC running time was 36 min. All mass spectra were acquired with an electron ionization system (EI, Electron Impact mode) with ionization energy of 70 eV and source temperature of 250 °C, with spectral acquisition in Full Scan mode, positive polarity, over a mass range of 40–600 Da with a scan rate of 750 amu/s.

The chromatogram acquisition, detection of mass spectral peaks and their waveform processing were performed using Xcalibur MS Software Version 2.1 (Thermo Scientific Inc.). Assignment of chemical structures to chromatographic peaks was based on the comparison with the databases for

GC-MS NIST Mass Spectral Library (NIST 08) and Wiley Registry of Mass Spectral Data (8th Edition).

GC-FID analyses were performed using a HP SERIES 5890 II equipped with a fire ion detector (FID, temperature 350 °C). Analytes were separated using a Restek Rtx-5MS (30 m×0.25 mm×0.25 μm) capillary column with nitrogen as a carrier gas at 1 mL min^{-1}. The injector temperature was 250 °C. The GC oven temperature was held at 80 °C for 2 min, increased to 250 °C by a temperature ramp of 10 °C min^{-1}, and held for 10 min. HPLC analyses have been performed by means of a JASCO system (Jasco LC-NET II/ ADC, Japan instrument, JASCO-PU980 pumps and JASCO-UV975 detector) using Thermo Fisher ODS-Hypersyl column C20 HPLC Column (25 cm × 4.6 mm, 5 μm particle size). Solvents used for the elution process were H_2O (with 0.1% HCO_2H) and MeCN, and a flow rate of 0.7 mL min^{-1} was adopted (ratio 98:2 for compounds **1** and 3:2 for compounds **3**). For the injection has been used a 20 μL coil autosampler. The UV detector was set to operate at 270 nm. Photochemical reactions in the visible region have been carried out with a 427 nm lamp (Kessil PR-160L, 45 W, for emission spectrum: https://kessil.com/products/science_PR160L.php) as the light source and a box equipped with a fan as the cooling system. Photochemical reactions in the UV region have been performed by using a multilamp apparatus fitted with 10×15 W phosphor-coated Hg lamps (emission centered at 310 nm). ***Caution!*** *Ultraviolet light is damaging to biological tissues. Caution is required when working with the lamp and protective eyewear must be used.*

For *in-vial* test a photobox has been employed while for 5 mL scale solutions the appropriate vessel was directly irradiated by the 427 nm Kessil PR-160L lamp (see Figures S9a, S9b).

1.2 Photophysical data for arylazo sulfonates 1a-o.

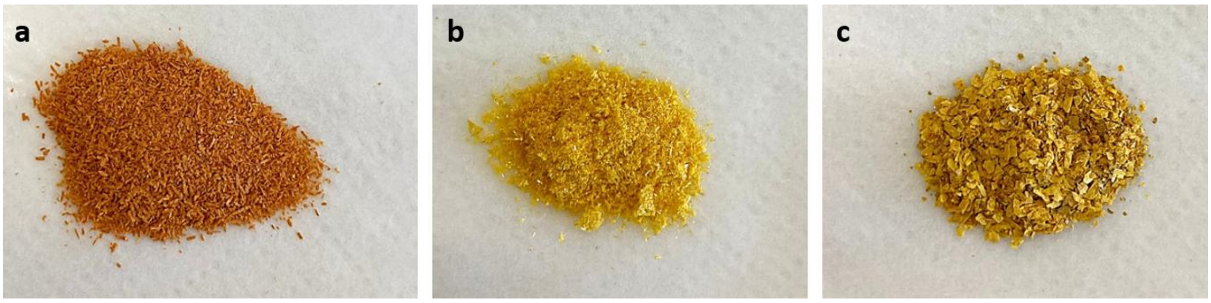

Figure S1. Representative images of sodium arylazo sulfonates salts used in this work: a) Sodium 2-(4-cyanophenyl)diazene-1-sulfonate (**1a**). b) Sodium 2-(4-chlorophenyl)diazene-1-sulfonate (**1f**). c) Sodium 2-(4-nitrophenyl)diazene-1-sulfonate (**1e**).

Table S1. Photophysical data of compounds **1a-o**.[a]

Compound	λ_{abs1}, nm (ε, M^{-1} cm^{-1})	λ_{abs2}, nm (ε, M^{-1} cm^{-1})
**1a**[a]	302 (13200)	421 (190)
**1b**[b]	304 (11700)	418 (210)
**1c**[a]	331 (15400)	411 (650)
**1d**[a]	288 (23200)	427 (240)
**1e**[a]	280 (30100)	428 (260)
**1f**[b]	300 (8400)	417 (130)
**1g**[a]	296 (16700)	412 (220)
**1h**[b]	276 (14200)	421 (130)
**1i**[b]	295 (4900)	432 (332)
**1j**[b]	292 (4800)	427 (150)
**1k**[b]	290 (8800)	406 (50)
**1l**[b]	300 (4300)	392 (146)
**1m**[b]	297 (5600)	428 (290)
**1n**[b]	268 (17200)	428 (470)
**1o**[b]	300 (4400)	424 (130)

[a] The ε values related to the low intensity band in the visible region were determined by using 10^{-3} M solutions of **1a-o** in neat H_2O, whereas 10^{-5} M solutions were used to determine the ε value in the UV region. [b] Analyses performed on 10^{-2} M and 10^{-4} M solutions, respectively.

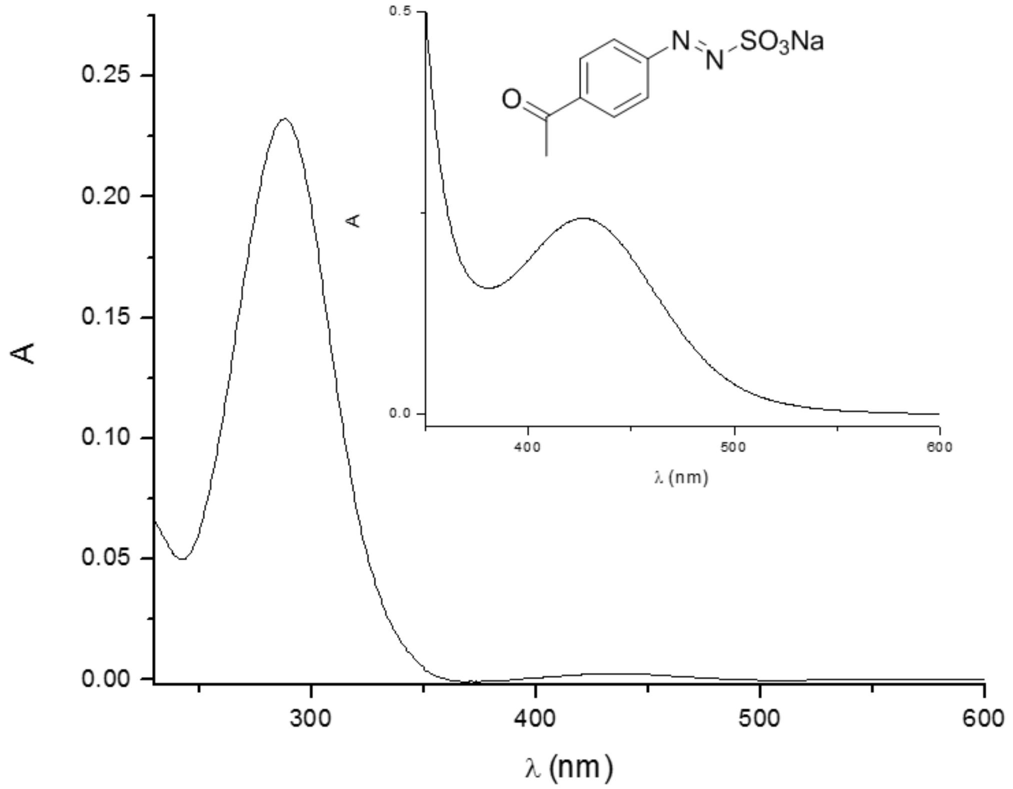

Figure S2a. UV-Vis spectrum of arylazo sulfonate **1d** (10^{-5} M) in H_2O. Inset: UV-Vis spectrum of **1d** in the visible region (10^{-3} M).

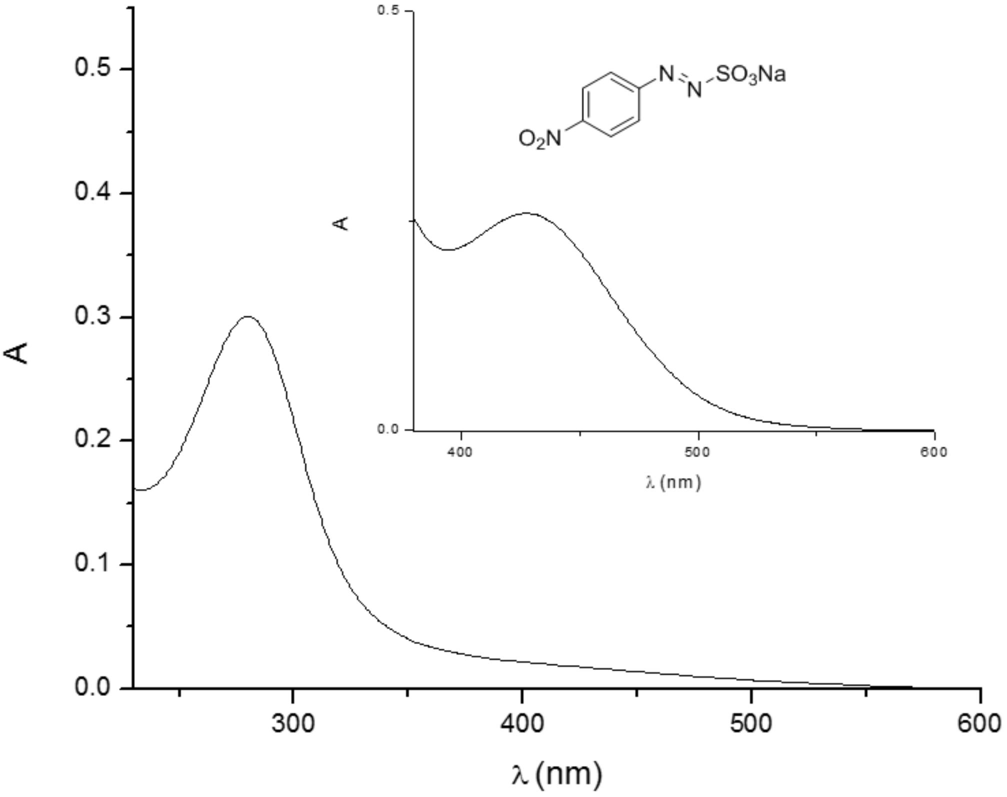

Figure S2b. UV-Vis spectrum of arylazo sulfonate **1e** (10^{-5} M) in H_2O. Inset: UV-Vis spectrum of **1e** in the visible region (10^{-3} M).

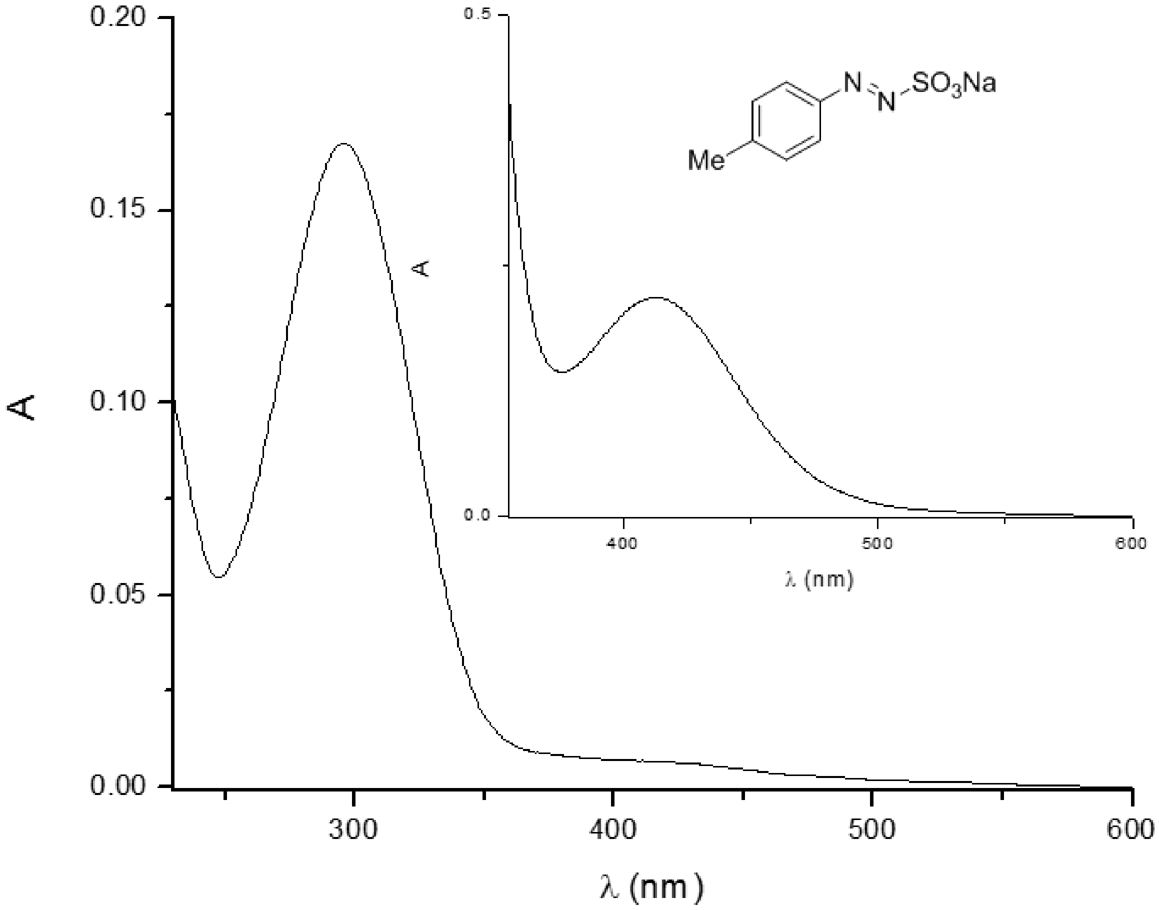

Figure S2c. UV-Vis spectrum of arylazo sulfonate **1g** (10^{-5} M) in H_2O. Inset: : UV-Vis spectrum of **1g** in the visible region (10^{-3} M).

Fluorescence measurements: Spectrofluorimetric analyses carried out on 10^{-4} M solutions of arylazo sulfonates **1a**, **1c** and **1f** in neat water showed that the compounds are not emitting neither under visible (425 nm) nor UV (290 nm) light irradiation.

1.3 Photochemical experiments.

1.3.1 General procedure for the determination of the quantum yield of decomposition (Φ_{-1}).

Quantum yields of decomposition (Φ_{-1}) were evaluated on the consumption of the chosen arylazo sulfonate **1**, in accordance with the reported literature.[S3] A stock solution was prepared and stored in the dark as much as possible, away from ambient light.

Stock solution: in a volumetric flask an arylazo sulfonate **1** (0.5 mmol) was dissolved in the chosen solvent (10 mL). The solution was kept away from the light wrapping the flask with an aluminium foil.

Test set-up: For each test, 3 mL of stock solution were placed in a quartz vials (10 mm optical path) and purged with nitrogen for 5 min. Once set the right reaction environment, the solution was irradiated for 30 sec using a 427 nm Kessil lamp at 5 cm distance in an optical bench equipped with a photon-counter behind the vial housing. The consumption of arylazo sulfonate **1** was determined by HPLC technique. HPLC analyses were performed by diluting solutions to 5×10^{-3} M with MeOH. This procedure has been repeated three times from the same stock solution to obtain a consistent set of data and the reported quantum yield resulted in the average value.

*Calculations (examples for **1c** in MeCN/H_2O (4:1) mixture as solvent)*

Quantum yield of decomposition Φ_{-1} was defined as follow:

$$\Phi_{-1} = \frac{n^\circ\ consumed\ molecules}{n^\circ\ absorbed\ photons}$$

It was so calculated in accordance with the following equation (*equation 2*):

$$\Phi_{-1} = \frac{V \times C_0 \times consumption}{Einstein_{Abs} \times t \times f}$$

where V is volume of solution in the quartz vial in L, C_0 is the initial concentration in mol×L^{-1} , t is the irradiation time in second.

The average consumption of the sample was calculated by means of HPLC analyses.

$$\overline{Consumption} = 1 - \frac{A_I}{A_0} = 0.15$$

$Einstein_{Abs}$ value was calculated as follows:

$$Einstein_{Abs} = \frac{I_{Incident}}{NA} = \frac{6.39 \times 10^{16}\ s^{-1}}{6.022 \times 10^{23}\ mol^{-1}} = 1.06 \times 10^{-7} mol \cdot s^{-1}$$

where $I_{incident}$ *was calculated as:*

$$I_{incident} = \frac{vial\ area \times irradiance\ without\ vial}{Energy\ at\ 427\ nm}$$

$$I_{incident} = \frac{3.2 \times 10^{-4}\ m^2 \times 93.0\ W \cdot m^{-2}}{4.7 \times 10^{-19}\ W \cdot s} = 6.39 \times 10^{16} s^{-1}$$

where *f* is the fraction of light absorbed, it was obtained by measuring the irradiance with or without the photolyzing solution on the optic bench and then applying the following equation:

$$f = 1 - \frac{irradiance\ whitout\ vial}{irradiance\ with\ vial} = 0.85$$

Filling back the *equations 2* we obtain:

$$\Phi_{-1} = \frac{0.003\ L \times 0.05\ mol \cdot L^{-1} \times 0.15}{1.06 \times 10^{-7} mol \cdot s^{-1} \times 30\ s \times 0.85} = 0.51$$

With the same method the values reported in Table S2 have been calculated.

Table S2. Summary of quantum yields of decomposition (Φ_{-1}) for the selected sulfonates **1** in the chosen solvents (0.05 M) irradiated for 30 sec at 427 nm.

**Substrate**	**Solvent**	**Φ_{-1}**
**1a**	MeCN/H_2O (4:1)	0.46
**1b**	MeCN/H_2O (4:1)	0.44
**1c**	MeCN/H_2O (4:1)	0.51
**1c**	MeCN/H_2O (3:1)	0.4
**1c**	Me_2CO/H_2O (9:1)	0.51
**1c**	EtOH/H_2O (2:1)	0.65
**1c**	H_2O	0.32

1.3.2 General procedure for the irradiation of 1 in neat solvents.

In a dried Pyrex (or quartz) vessel, arylazo sulfonate **1** (0.05 mmol) was dissolved in the chosen solvent mixture (1 mL). An inert atmosphere was settled up by purging nitrogen into the solution for 3 min, then the vial was capped. The solution was then irradiated with a 427 nm Kessil Lamp (Figure S9a) (or 310 nm phosphor-coated lamps) at room temperature in a photochemical reactor for 24 h. The consumption of **1** has been quantified by HPLC analysis. Conversion to arenes **2** has been evaluated by GC-FID analysis and quantified via calibration curves with internal standard (dodecane 0.5 µL/mL); phenols **3** in the photolyzed solution has been quantified by HPLC via calibration curves.

1.3.3 General procedure for photochemical hydrodeamination and deuteration of 1

In a dried Pyrex vessel, arylazo sulfonate **1** (0.025 mmol) was dissolved in the chosen solvent mixture (1 mL). An inert atmosphere was settled up by purging nitrogen into the solution for 3 min, then the vial was capped. The solution was then irradiated with a 427 nm Kessil Lamp at room temperature in a photochemical reactor for 2 h (Figure S9a). Conversion to **2** has been evaluated by GC-FID analysis and quantified via calibration curves with internal standard (dodecane 0.5 µL/mL). Deuteration ratio has been quantified by GC-MS analysis by comparison of the intensity of the appropriate mass picks and then normalized (see Figures S3-S8).

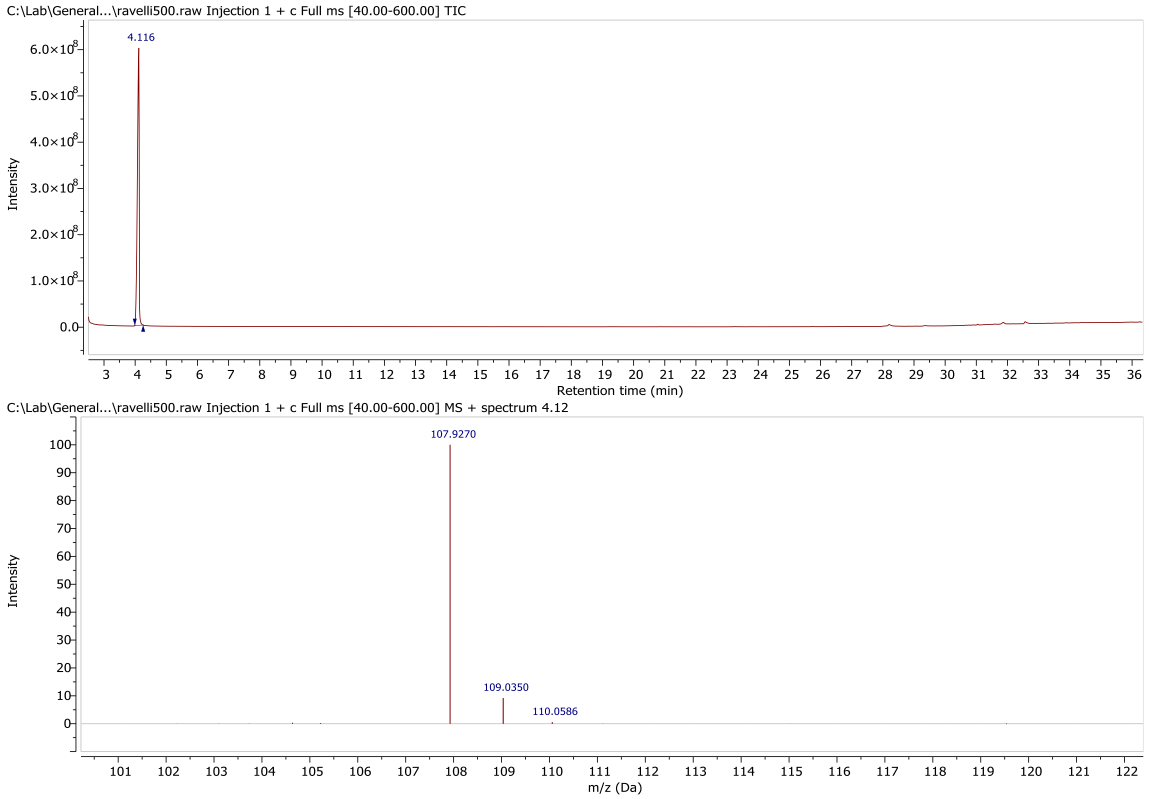

Figure S3. GC/MS analysis results for the photolysis of **1c** in MeOD: chromatogram (top),

mass spectrum for peak at 4.116 min (bottom).

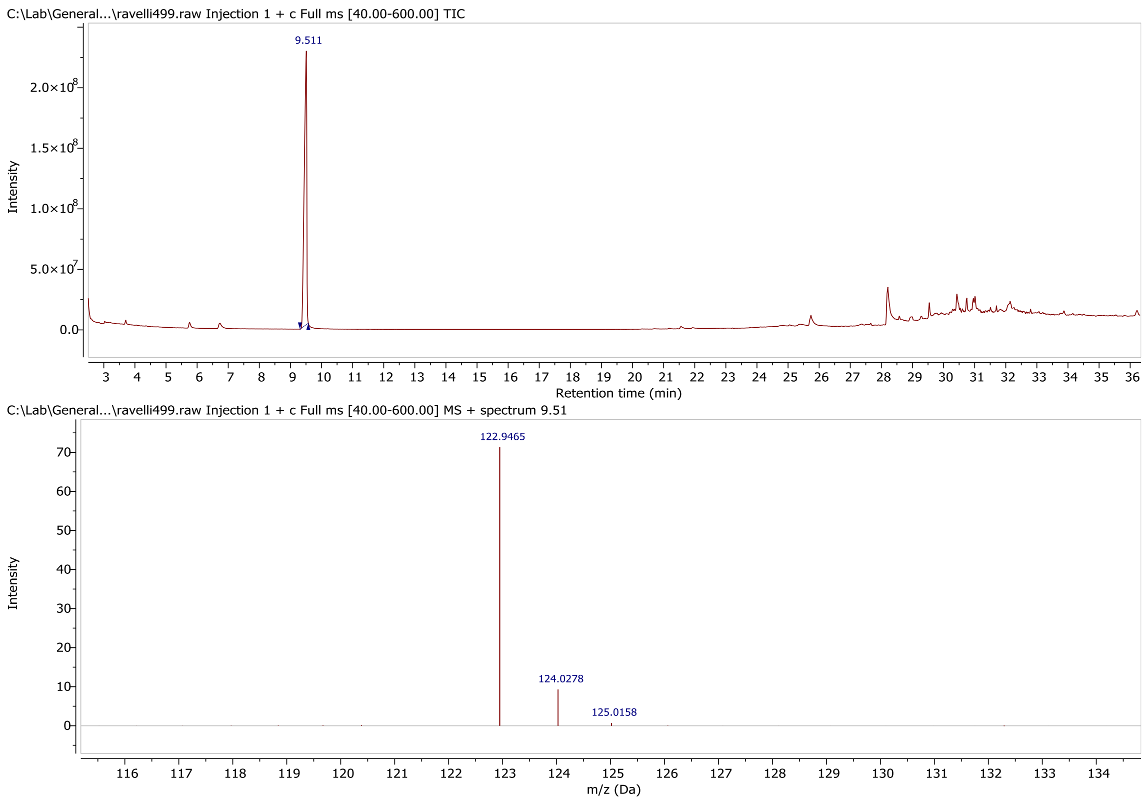

Figure S4. GC/MS analysis results for the photolysis of **1e** in MeOD: chromatogram (top), mass spectrum for peak at 9.511 min (bottom).

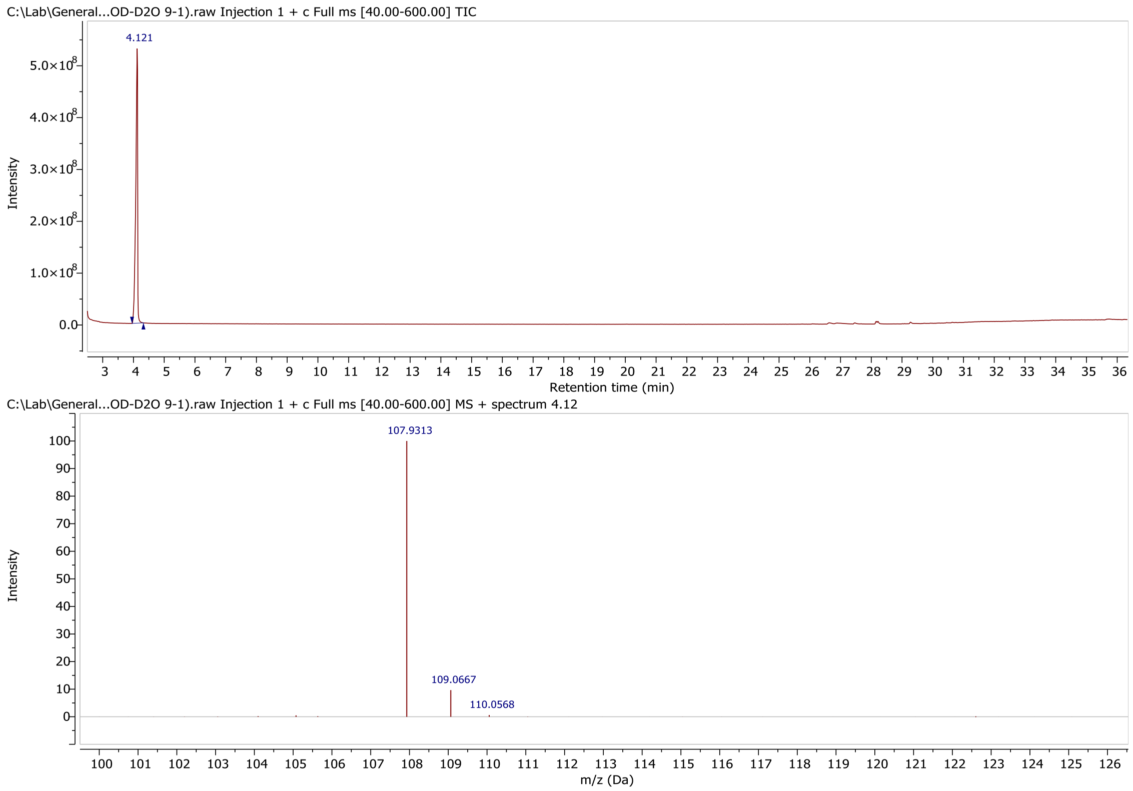

Figure S5. GC/MS analysis results for the photolysis of **1c** in MeOD/D_2O (9:1): chromatogram (top), mass spectrum for peak at 4.121 min (bottom).

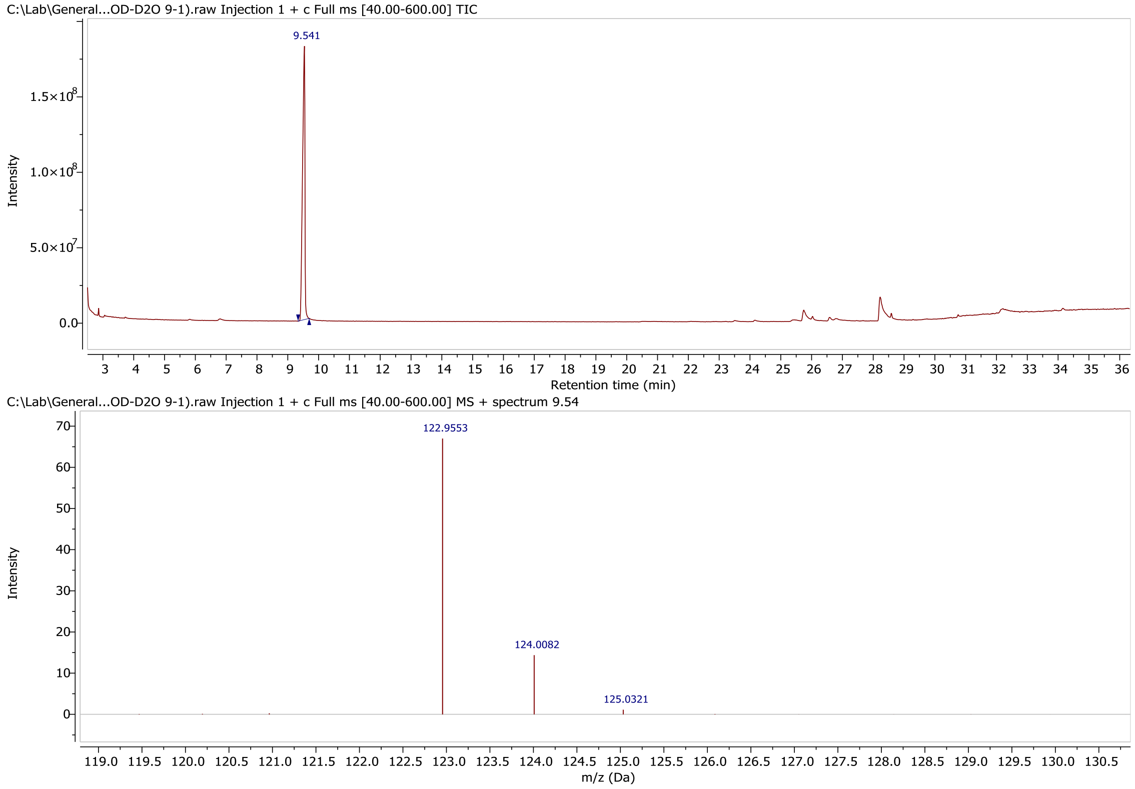

Figure S6. GC/MS analysis results for the photolysis of **1e** in MeOD/D_2O (9:1): chromatogram (top), mass spectrum for peak at 9.541 min (bottom).

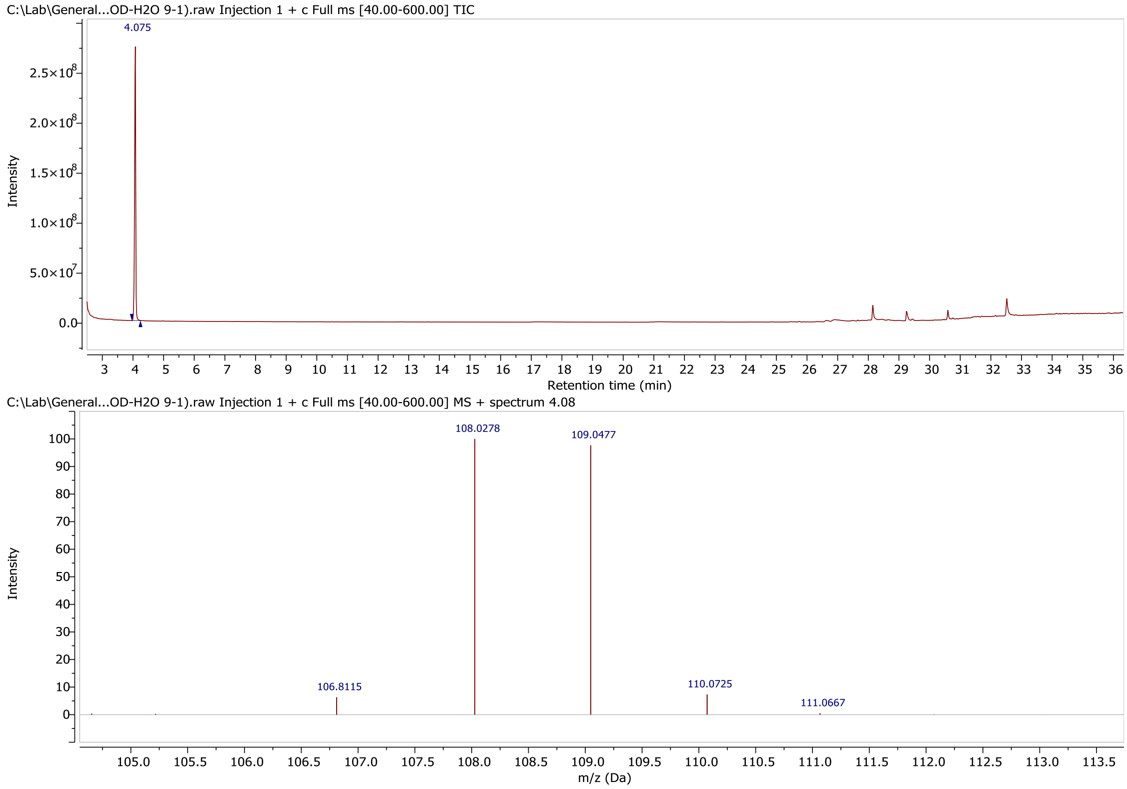

Figure S7. GC/MS analysis results for the photolysis of **1c** in CD_3OD/H_2O (9:1): chromatogram (top), mass spectrum for peak at 4.075 min (bottom).

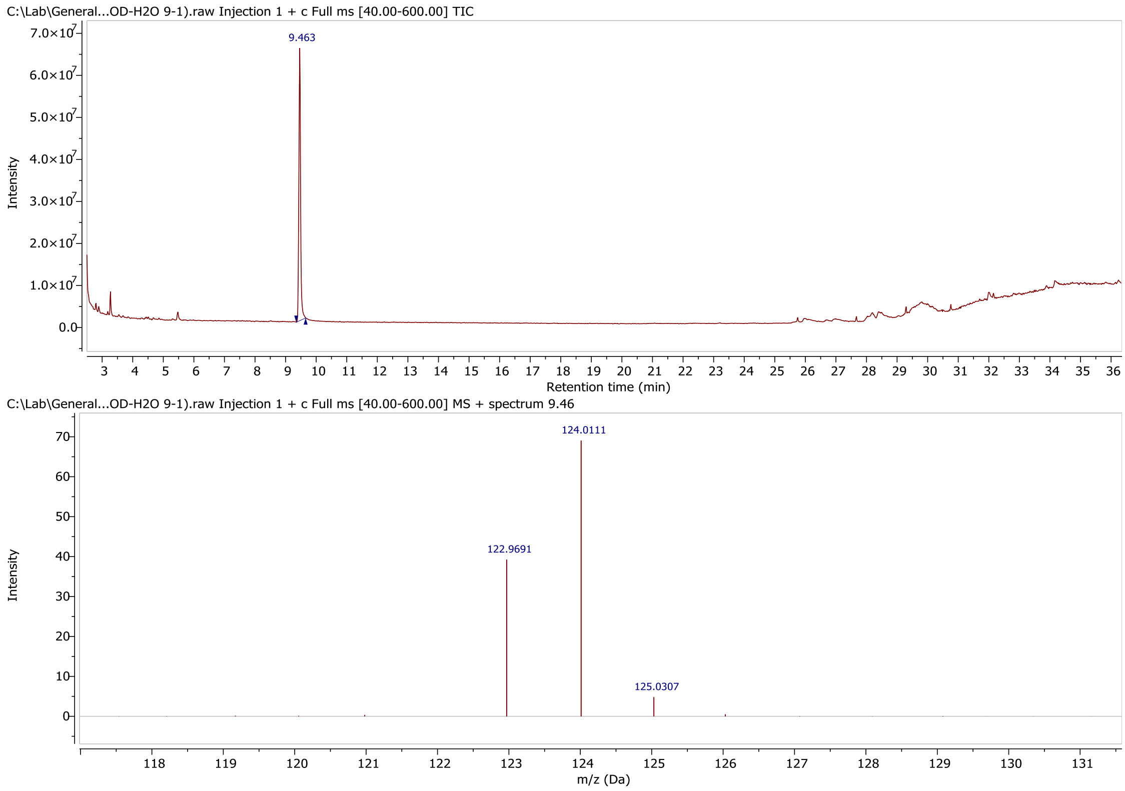

Figure S8. GC/MS analysis results for the photolysis of **1e** in CD_3OD/H_2O (9:1): chromatogram (top), mass spectrum for peak at 9.463 min (bottom).

Analyses of the obtained GC-MS spectra revealed:

- From Figure S3: a mixture of **2c** (107.93) and **2c**-*d* (109.04) in 12:1 ratio
- From Figure S4: a mixture of **2e** (122.95) and **2e**-*d* (124.03) in 8:1 ratio
- From Figure S5: a mixture of **2c** (107.93) and **2c**-*d* (109.07) in 11:1 ratio
- From Figure S6: a mixture of **2e** (122.95) and **2e**-*d* (124.01) in 5:1 ratio
- From Figure S7: a mixture of **2c** (108.03) and **2c**-*d* (109.05) in 11:10 ratio
- From Figure S8: a mixture of **2e** (122.97) and **2e**-*d* (124.01) in 5:9 ratio

1.3.4 Irradiation of arylazo sulfonates in the presence of nucleophiles.

In a dried glass vessel, arylazo sulfonate **1** (0.01-0.1 mmol) was dissolved in the chosen solvent mixture (1 mL). An inert atmosphere was settled up by purging nitrogen into the solution for 3 min, then the nucleophile (10-20 equiv.) was added and the vessel capped. The solution was then irradiated with a 427 nm Kessil Lamp at room temperature in a photochemical reactor for 24 h (Figure S9b).

Consumption of **1** has been quantified by HPLC analysis comparing chromatographic areas before and after irradiation. Conversion to derivates **2**, **6** and **7** has been evaluated by GC-FID analysis and quantified via calibration curves with internal standard (dodecane 0.5 µL/mL).

In selected cases, reactions have been performed on a 0.05-0.5 mmol scale to isolate and characterize the products. After irradiation, solvent was removed and products **6**, **9** or **10** were purified by silica gel flash column chromatography (eluent mixture CyHex/EA).

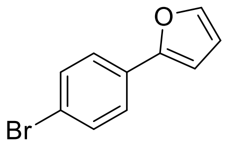

2-(4-Bromophenyl)-furan (**6b**): From 71.9 mg (0.25 mmol, 0.05 M) of **1b** and 365 µL (5.0 mmol, 20 equiv.) of furan in 5 mL mixture MeCN/H_2O 4:1. Purification by silica gel flash column chromatography (eluant mixture, CyHex/EA from 99:1 to 9:1) afforded product **6b** in 46% of yield (pale yellow viscous oil, 23.4 mg). Spectroscopic data are in accordance with literature.[S1]

6b. ^{1}H NMR (300 MHz, Chloroform-*d*) δ 7.66–7.42 (m, 5H), 6.71–6.44 (m, 2H). ^{13}C{^{1}H}NMR (75 MHz, Chloroform-*d*) δ 152.9, 142.3, 131.7, 129.7, 125.2, 120.9, 111.7, 105.4.

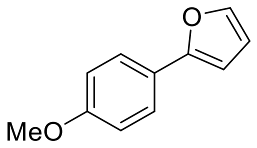

2-(4-Methoxyphenyl)-furan (**6c**): From 59.5 mg (0.25 mmol, 0.05 M) of **1c** and 365 µL (5.0 mmol, 20 equiv.) of furan in 5 mL mixture MeCN/H_2O 4:1. Purification by silica gel flash column chromatography (eluant mixture, CyHex/EA from 99:1 to 8:2) afforded product **6c** in 34% of yield (colorless viscous oil, 14.8 mg). Spectroscopic data are in accordance with literature.[S2]

6c. ^{1}H NMR (300 MHz, Chloroform-*d*) δ 7.66–7.60 (m, 2H), 7.45 (dd, *J* = 1.8, 0.8 Hz, 1H), 6.98–6.92 (m, 2H), 6.54 (dd, *J* = 3.3, 0.8 Hz, 1H), 6.47 (dd, *J* = 3.3, 1.8 Hz, 1H), 3.86 (s, 3H). ^{13}C{^{1}H}NMR (100 MHz, Chloroform-*d*) δ 159.0, 154.0, 141.4, 125.2, 124.0, 114.1, 111.5, 103.4, 55.3.

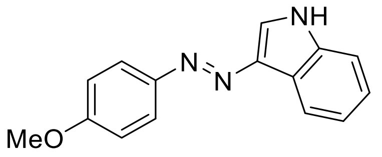

3-((4-Methoxyphenyl)diazenyl)-1H-indole (9c): From 59.5 mg (0.25 mmol, 0.05 M) of **1c** and 586 mg (5.0 mmol, 20 equiv.) of indole in 5 mL H_2O. Purification by silica gel flash column chromatography (eluant mixture, CyHex/EA from 99:1 to 80:10) afforded product **9c** in 46% of yield (29.1 mg, yellow oil).

9c. ^{1}H NMR (300 MHz, Chloroform-*d*) δ 8.66–8.51 (m, 2H), 7.97 (d, *J* = 2.9 Hz, 1H), 7.91–7.84 (m, 2H), 7.42–7.37 (m, 1H), 7.33–7.28 (m, 2H), 7.03–6.98 (m, 2H), 3.89 (s, 3H). ^{13}C{^{1}H}NMR (75 MHz, Chloroform-*d*) δ 160.8, 148.0, 136.9, 136.4, 129.4, 124.3, 123.5, 123.2, 122.9, 119.3, 114.3, 111.4, 55.7. HRMS (ESI) m/z: calcd. for $C_{15}H_{15}N_3O$ $[M+H]^+$: 252.1131, found: 252.1126.

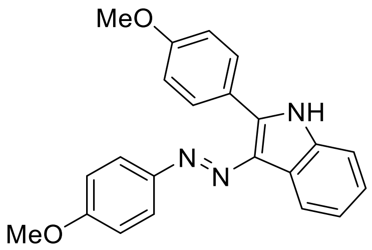

2-(4-Methoxyphenyl)-3-((4-methoxyphenyl)diazenyl)-1H-indole (10c): From 59.5 mg (0.25 mmol, 0.05 M) of **1c** and 586 mg (5.0 mmol, 20 equiv.) of indole in 5 mL H_2O. Purification by silica gel flash column chromatography (eluant mixture, CyHex/EA from 99:1 to 85:15) afforded product **10c** in 29% of yield (26 mg, yellow oil).

10c. ^{1}H NMR (300 MHz, Chloroform-*d*) δ 8.71–8.59 (m, 1H), 8.48 (s, 1H), 7.98 (d, *J* = 8.8 Hz, 2H), 7.87 (d, *J* = 8.9 Hz, 2H), 7.42–7.36 (m, 1H), 7.32–7.27 (m, 2H), 7.07 (d, *J* = 8.8 Hz, 2H), 7.01 (d, *J* = 8.9 Hz, 2H), 3.93–3.86 (m, 6H). ^{13}C{^{1}H}NMR (75 MHz, Chloroform -*d*) δ 160.6, 160.5, 148.8, 135.5, 132.0, 130.6, 124.3, 123.8, 123.7, 123.1, 120.5, 114.5, 114.3, 110.8, 55.7, 55.6. HRMS (ESI) m/z: calcd. for $C_{22}H_{20}N_3O_2$ $[M+H]^+$: 358.1550, found: 358.1543.

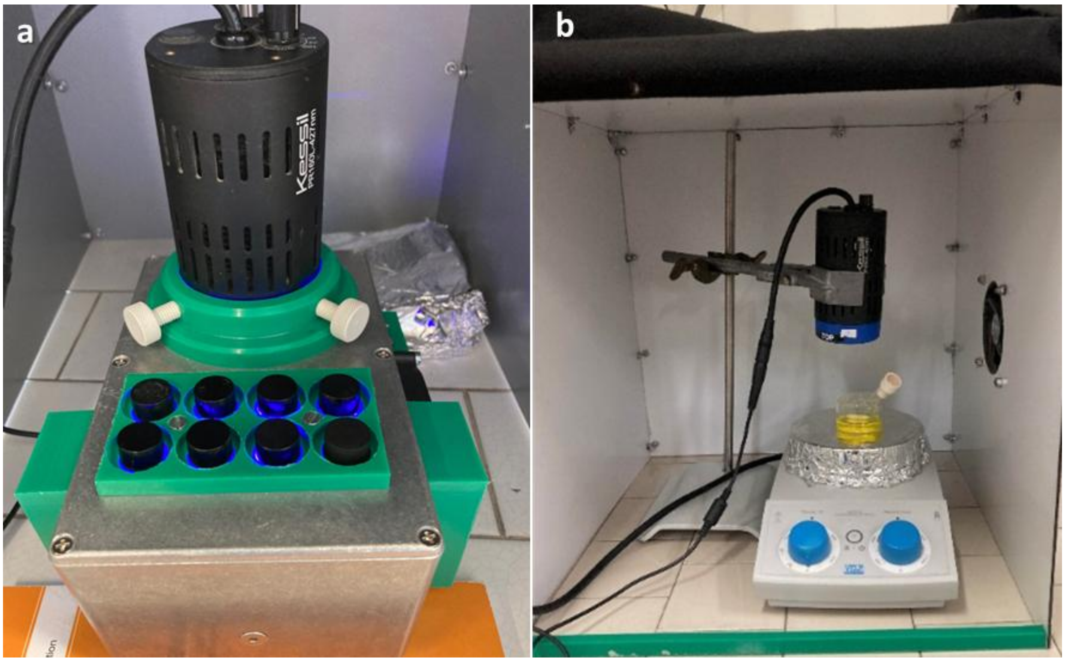

Figure S9. Photochemical set-ups. a) In vials experiments: a fan-cooled photoreactor accommodates a 427 nm Kessil Lamp and vials; the system is placed in a box equipped with a fan to keep reaction temperature equal to ambient. b) Large scale experiments: a glass vessel containing the solution to photolyze and a stirring bar is placed on a stirring plate, a 427 nm Kessil is placed 5 cm above; the irradiation system is placed in a box equipped with a fan to keep reaction temperature equal to ambient.

1.4 Mechanistic experiments

1.4.1 Kinetics experiments of photochemical consumption

Kinetics experiments were performed following the consumption of the chosen arylazo sulfonate **1**. In a volumetric flask the chosen arylazo sulfonate **1** (0.5 mmol) is dissolved in MeCN/H_2O 4:1 mixture as solvent (10 mL). The solution was distributed in 6 (or 7) vials, 1 mL each one. The vials were purged with nitrogen for 3 min to remove oxygen, corked and then irradiated for a specific period by a 427 nm Kessil lamp in a photoreactor. The consumption values at each period were obtained by related HPLC areas before and after irradiation. HPLC analyses were performed by diluting solutions to 5×10^{-3} M with MeOH.

Obtained values are reported in Table S3 and sketched in Figure S10.

Table S3. Kinetic values for the analyzed arylazo sulfonates **1a**, **1b** and **1c**

Substrate	k (s^{-1})	$t_{1/2}$ (sec)
**1a**	0.0013	533
**1b**	0.0012	577
**1g**	0.017	408

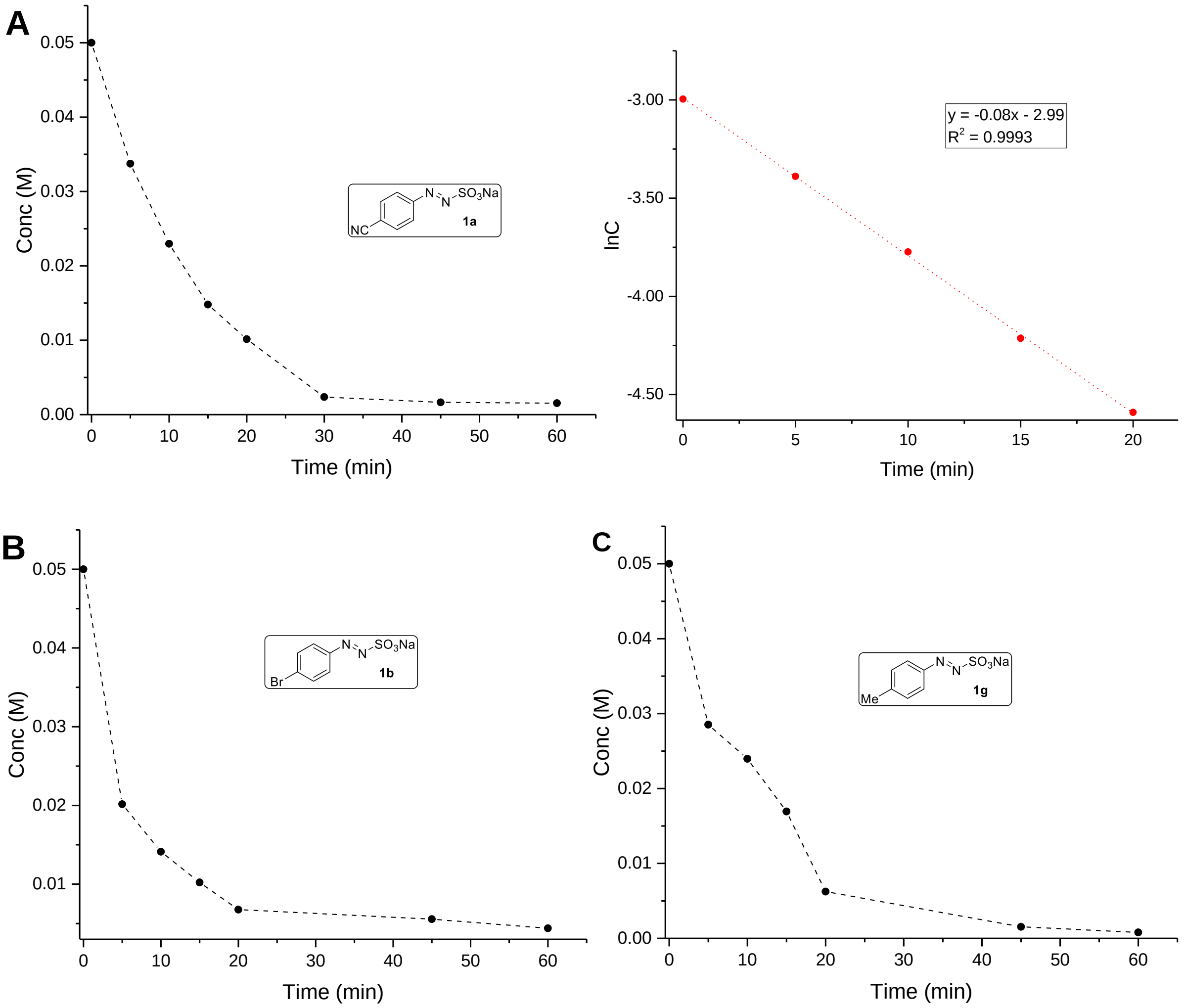

Figure S10. A) Kinetics of consumption of **1a** (left) and its linearization (right); B) Kinetics of consumption of **1b**; C) Kinetics of consumption of **1g**.

1.4.2 UV-absorption spectrum of compound 1c in the presence of increasing amount of indole.

The feasibility of arylazo sulfonates to form an EDA has been tested following variations of the UV-Visible spectrum of **1c** (in the visible region) by addition of incremental quantities of indole. A stock solution of indole (0.3 M in MeOH, 175.8 mg in 5 mL) has been prepared; after dilution to 5×10^{-3} M an UV-Vis spectrum has been registered (no absorption in the visible region has been detected, Figure S11). A 5×10^{-3} M (59.6 mg in 50 mL) solution of **1c** in MeOH has been prepared and an UV-Vis spectrum has been registered. To the same solution of **1c** incremental quantities of indole have been added (see Figure S11) and for each the absorption has been measured in the selected wavelengths region. The seven acquisitions are graphically summarized in Figure S11.

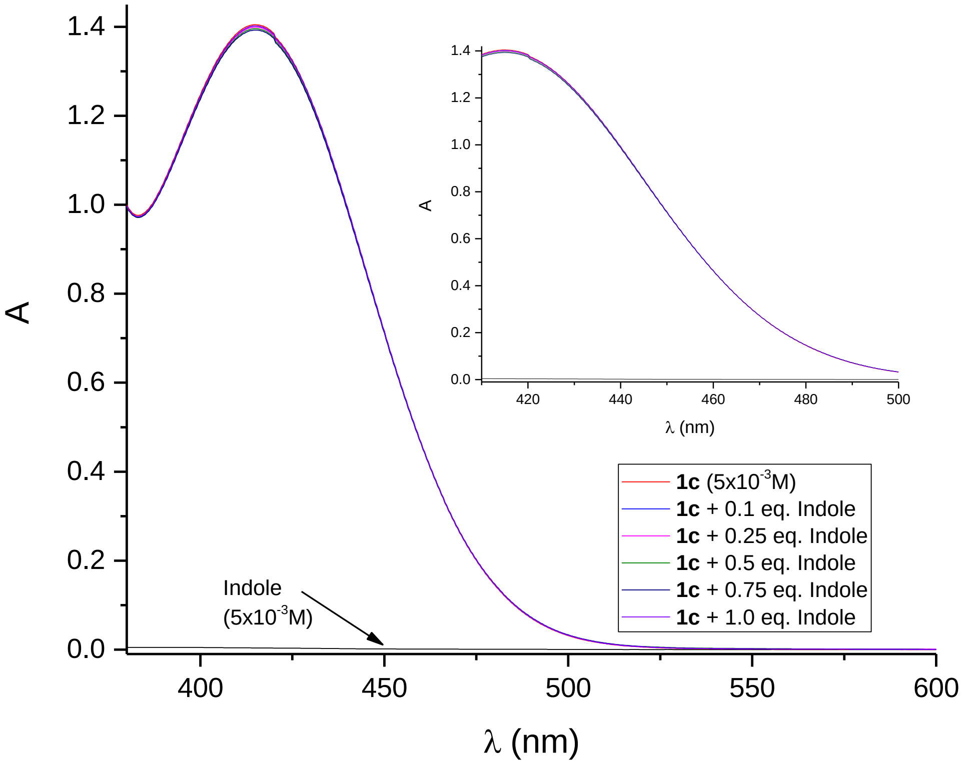

Figure S11. UV-Vis spectra of indole (5×10^{-3} M) in MeOH along with **1c** (5×10^{-3} M) in MeOH plotted together with increasing amount of indole.

Figure S11 showed no significant variations of the **1c** UV-Vis spectrum. In particular, no shifting of the absorption maximum in the visible region as well as any isosbestic points have been observed. These observations suggest that an EDA-complex between **1c** and indole can be safely excluded.

1.4.3 Trapping experiment with 2,2,6,6-Tetramethyl-1-piperidinyloxy radical (TEMPO)

In a dried glass vessel, 71.8 mg of arylazo sulfonate **1a** (0.25 mmol) and 390.6 mg of (2,2,6,6-Tetramethylpiperidin-1-yl)oxyl (TEMPO, 2.5 mmol, 1.0 equiv.) were dissolved in MeCN/H_2O 4:1 (5 mL). The solution was purged with nitrogen for 10 min, capped and irradiated with a 427 nm Kessil lamp for 24 h (see Figure S9b). After total consumption of arylazo sulfonate, the solvent was evaporated under vacuum and the resulting residue purified by silica gel column chromatography (eluent mixture CyHex/EA) affording 37.2 mg of adduct **8a** (57%, yellow solid). GC-FID analysis of the crude mixture allows to quantify the amount of **2a** (3% GC yield by means of a calibration curve) produced during the photochemical reaction.

Spectroscopic data of **8a** are in accordance with literature.[S4]

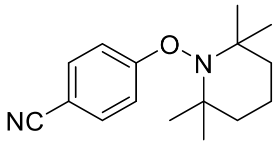

4-((2,2,6,6-Tetramethylpiperidin-1-yl)oxy)benzonitrile (8a). ^{1}H NMR (300 MHz, Chloroform-*d*) δ 7.47–7.41 (m, 2H), 7.19 (s, 2H), 1.57–1.48 (m, 5H), 1.36–1.35 (m, 1H), 1.16 (s, 7H), 0.90 (s, 6H). $^{13}C\{^{1}H\}$NMR (75 MHz, Chloroform-*d*) δ 167.1, 133.6, 119.6, 115.0, 103.5, 61.0, 39.9, 32.5, 20.6, 17.1.

1.5 Thermal behavior of 1c

In a round-bottom flask equipped with a refrigerator, 59.6 mg of arylazo sulfonate **1c** (0.25 mmol) were dissolved in H_2O (5 mL). The solution (prevented by light) was purged with nitrogen for 10 min and heated at reflux for 48 h by means of a heating mantle. After this period, HPLC analysis revealed that **1c** is in part consumed (88%) leading to phenol **3c** (89%, based on **1c** conversion). Extraction of the crude by ethyl acetate followed by GC-FID analysis evidenced the presence of trace amounts of **2c**.

1.6 Free Radical polymerization (FRP) experiment

Photoinitiated FRP tests have been performed on water soluble acrylamide by using arylazo sulfonate **1g** as a radical initiator. Thus, 5 g of acrylamide (0.07 mol) were dissolved in H_2O (5 mL) 2.5 mg of **1g** (0.02 mol%) was then added (Figure S12a). The resulting solution has been irradiated at 427 nm for 15 min at room temperature. A marked jellification of the resulting solution was apparent (Figure S12b). Additional tests carried out without light (Figure S12c) or without **1g** (Figure S12d) indicated that no polymerization took place.

Caution! Acrylamide may be a carcinogenic chemical and may constitute significant safety hazards and must be handled with extreme care.

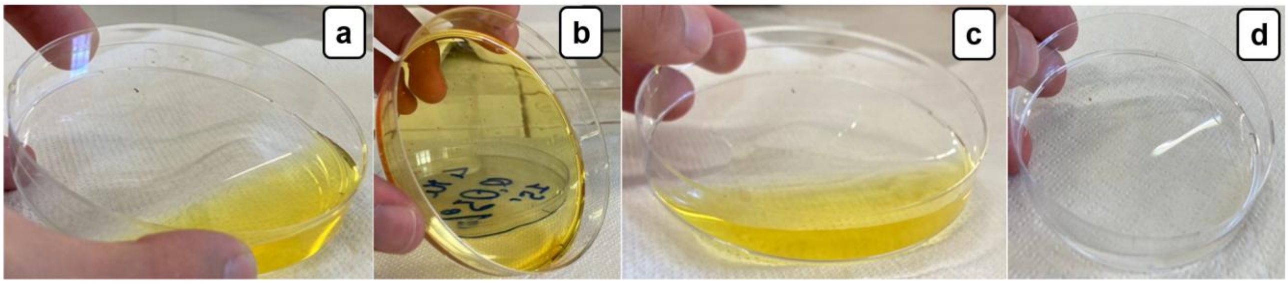

Figure S12. FRP test: a) Solution before irradiation; b) after irradiation for 15 min at room temperature; Blank experiments in the absence of (c) light; d) **1g**.

2. References

(S1) Ding, R.; Liu, Q.; Zheng, L. Piezoelectric Metal-Organic Frameworks Mediated Mechanoredox Borylation and Arylation Reactions by Ball Milling. *Chem. Eur. J.* **2023**, *29*, e202203792.

(S2) Crespi, S; Protti, S.; Fagnoni, M. Wavelength Selective Generation of Aryl Radicals and Aryl Cations for Metal-Free Photoarylations. *J. Org. Chem.* **2016**, *81*, 9612−9619.

(S3) Nicchio, L.; Di Terlizzi, L.; Fagnoni, M.; Neuville, L.; Protti, S.; Masson, M. Visible-Light Enabled Synthesis of 1-Aryl-3-Sulfonylmethyl-1,2,4-Triazoles by Arylazo Sulfones. *Adv. Synth. Catal.* **2024**, *366*, DOI:10.1002/adsc.202401251.

(S4) Mohit, Kumar, S.; Thomas, K. R. J. Hydrazone-Linked Donor-Acceptor Covalent Organic Polymer as a Heterogeneous Photocatalyst for C−S Bond Formation. *Chem. Eur. J.* **2024**, *30*, e202402196.

3. Copy of the NMR spectra

Sodium 2-(4-cyanophenyl)diazene-1-sulfonate (**1a**) ^{1}H NMR (300 MHz, DMSO-d_6).

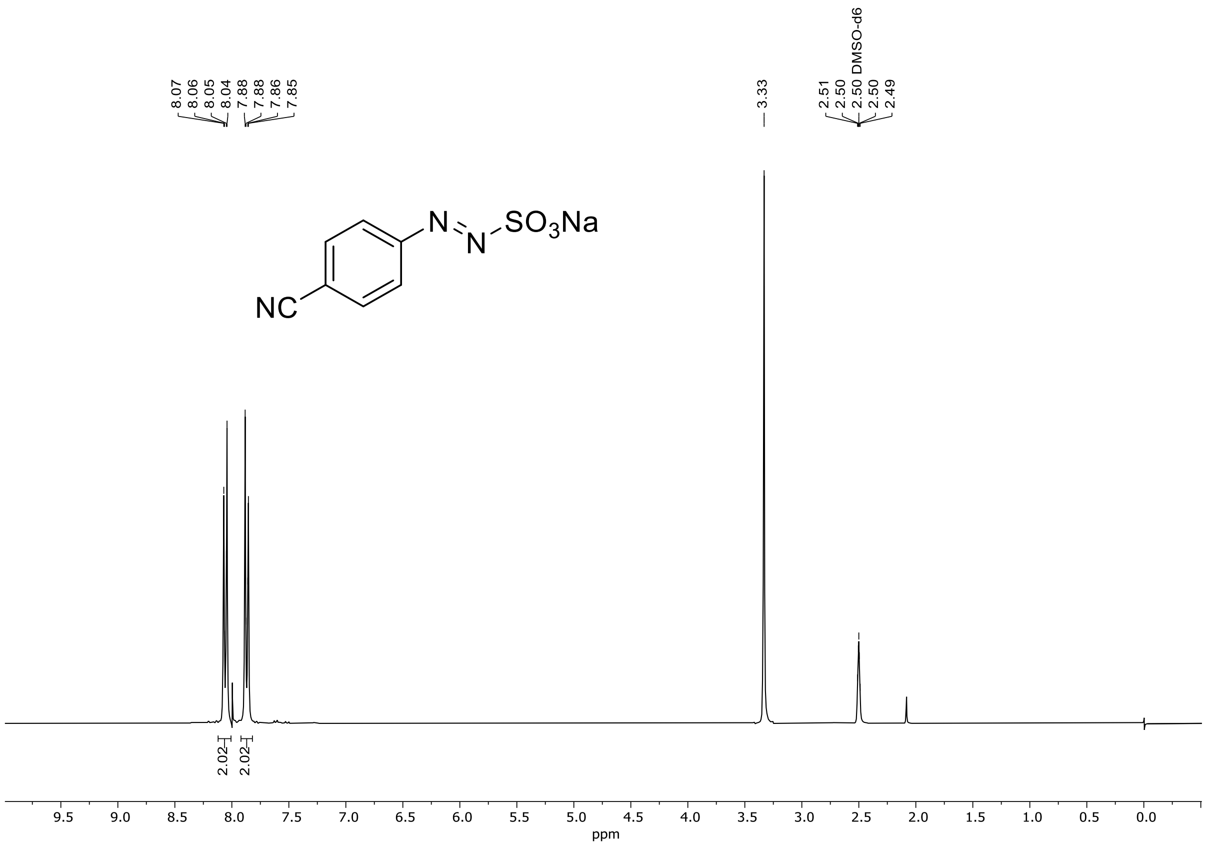

Sodium 2-(4-cyanophenyl)diazene-1-sulfonate (**1a**) $^{13}C\{^{1}H\}$ NMR (75 MHz, DMSO- d_6).

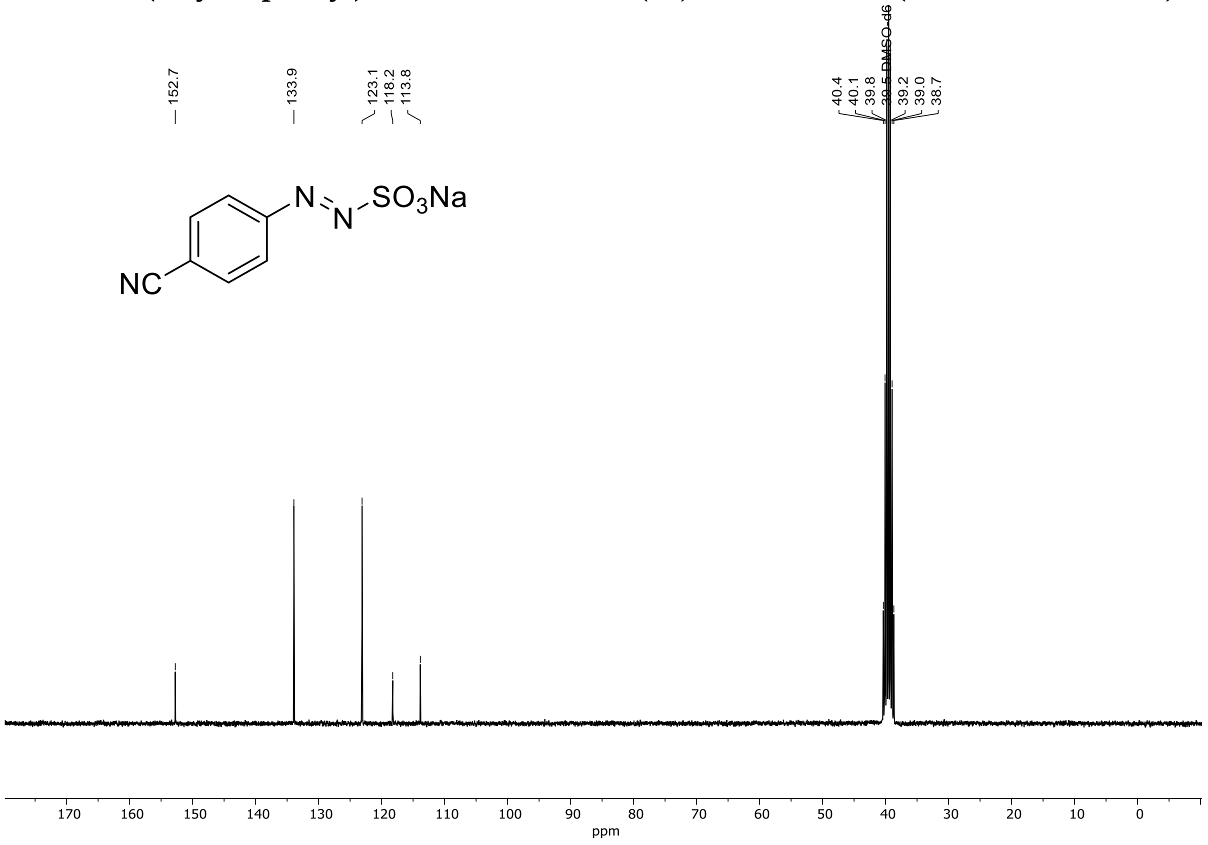

Sodium 2-(4-bromophenyl)diazene-1-sulfonate (**1b**) ^{1}H NMR (300 MHz, DMSO- *d*$_6$).

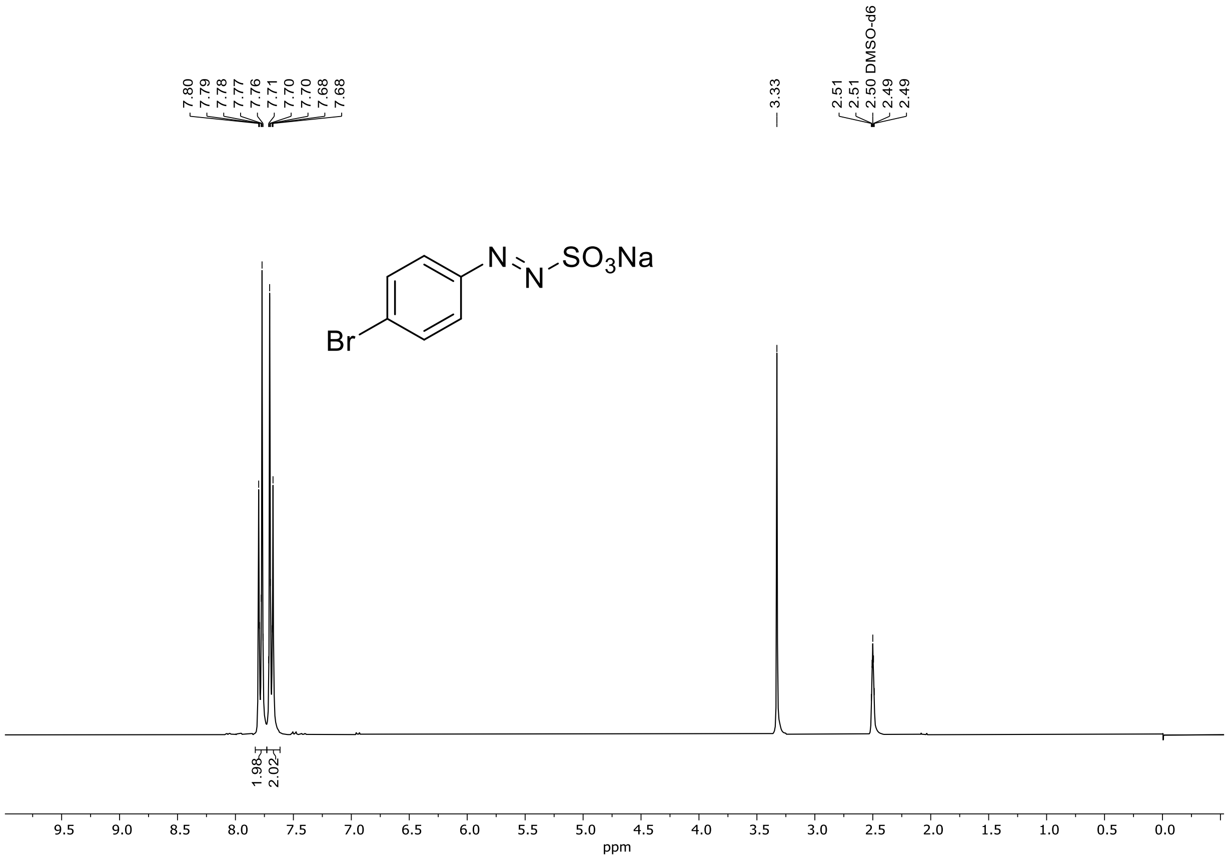

Sodium 2-(4-bromophenyl)diazene-1-sulfonate (**1b**) $^{13}C\{^{1}H\}$ NMR (75 MHz, DMSO- *d*$_6$).

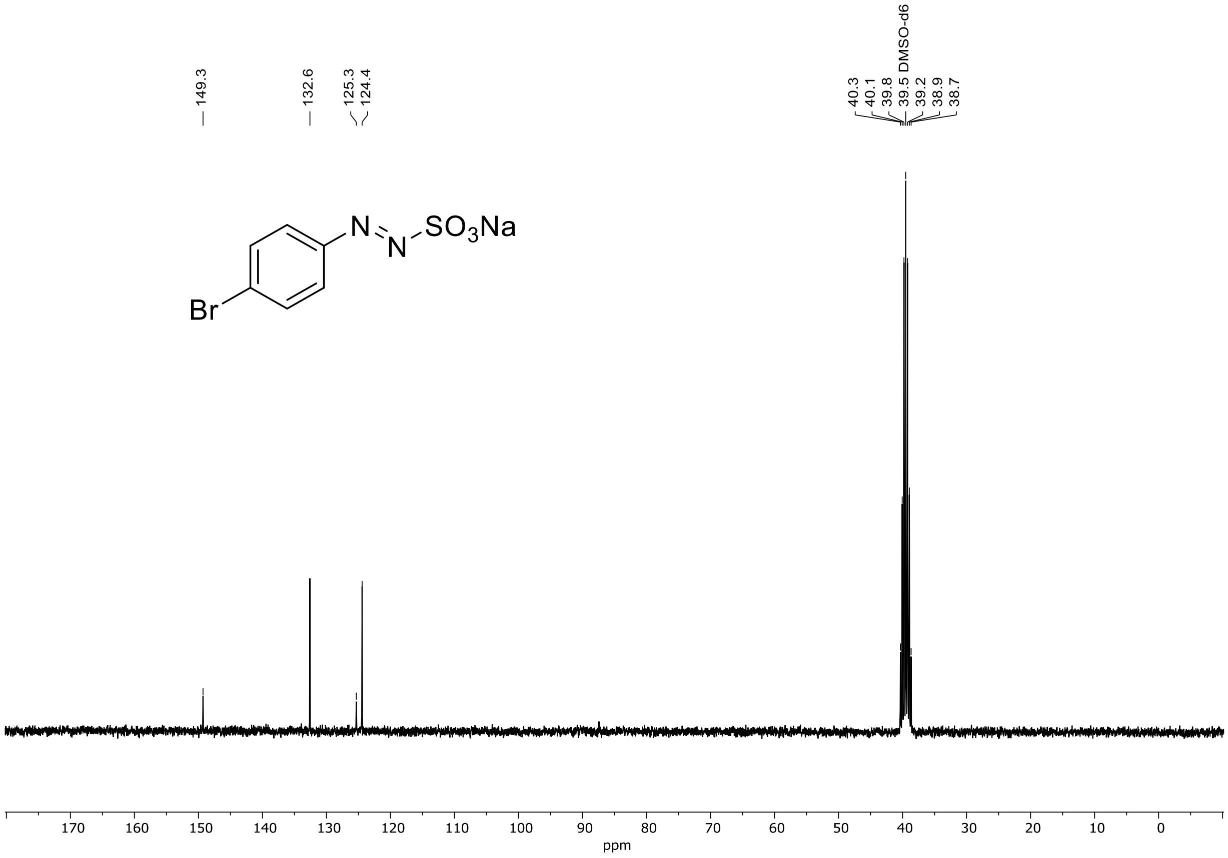

Sodium 2-(4-methoxyphenyl)diazene-1-sulfonate (**1c**) ^{1}H NMR (300 MHz, DMSO- d_6).

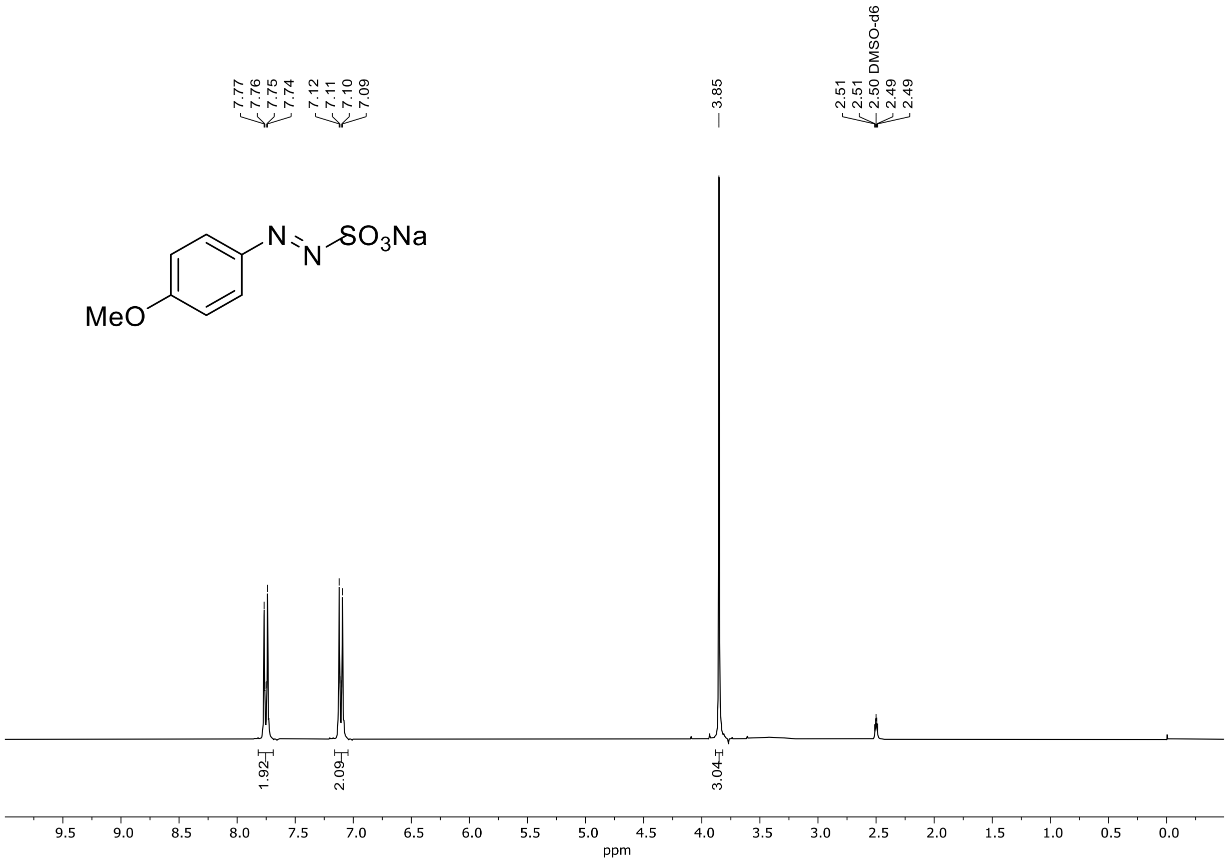

Sodium 2-(4-methoxyphenyl)diazene-1-sulfonate (**1c**) $^{13}C\{^1H\}$ NMR (75 MHz, DMSO- d_6).

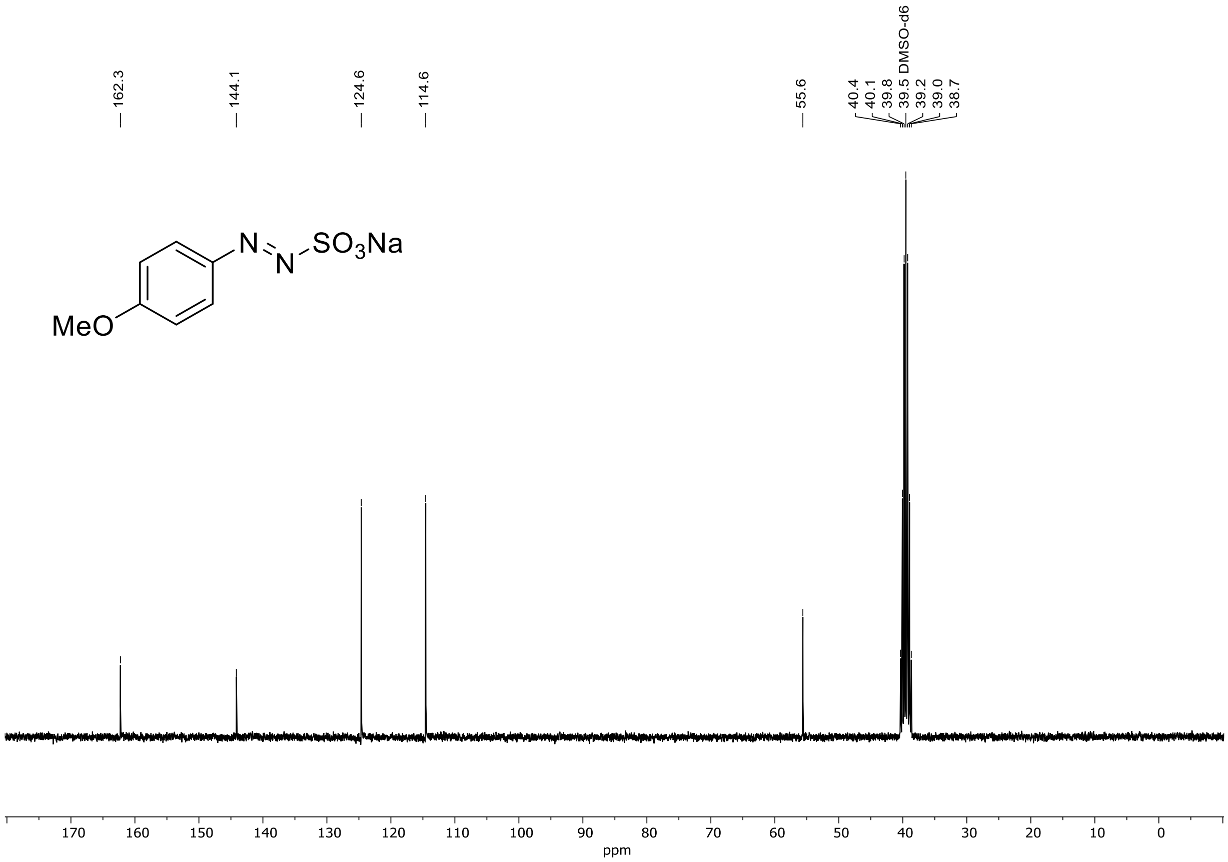

Sodium 2-(4-acetylphenyl)diazene-1-sulfonate (**1d**) ^{1}H NMR (300 MHz, DMSO- d_6).

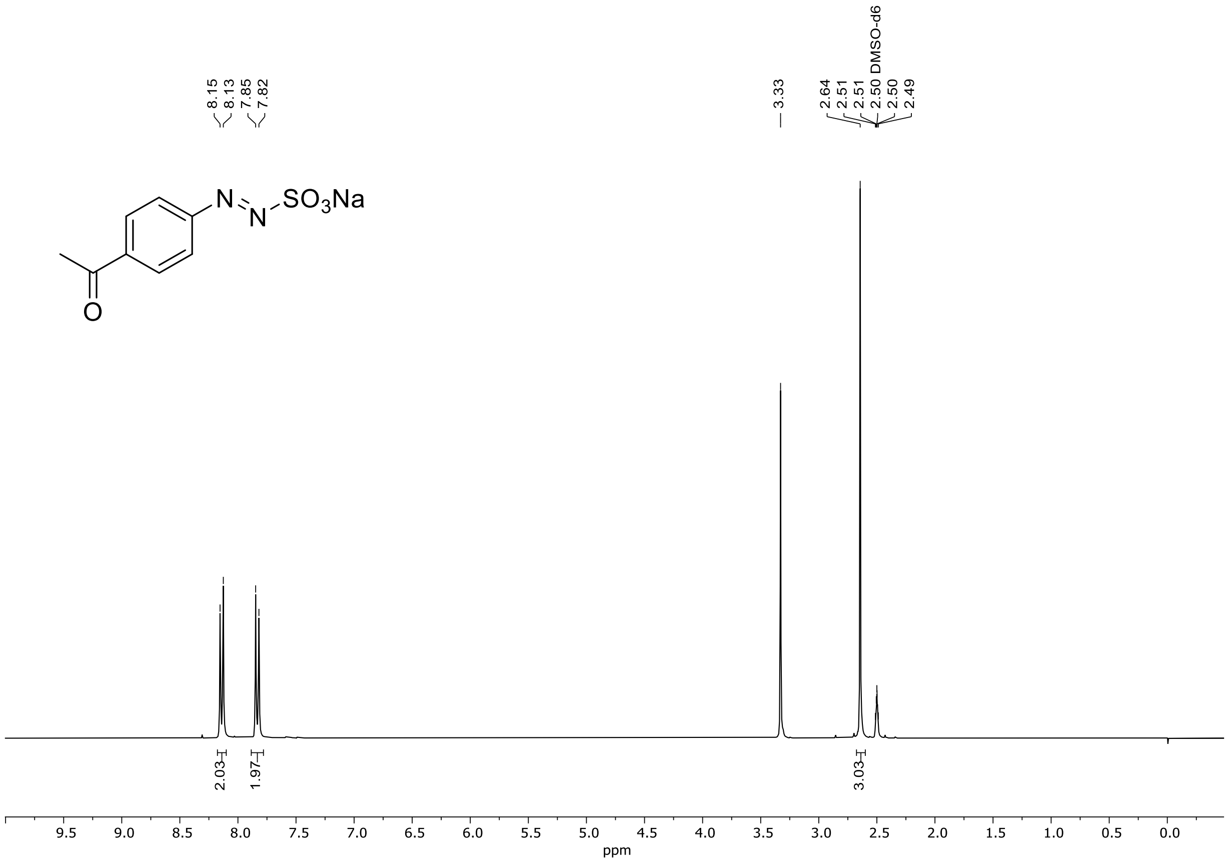

Sodium 2-(4-acetylphenyl)diazene-1-sulfonate (**1d**) $^{13}C\{^{1}H\}$ NMR (75 MHz, DMSO- d_6).

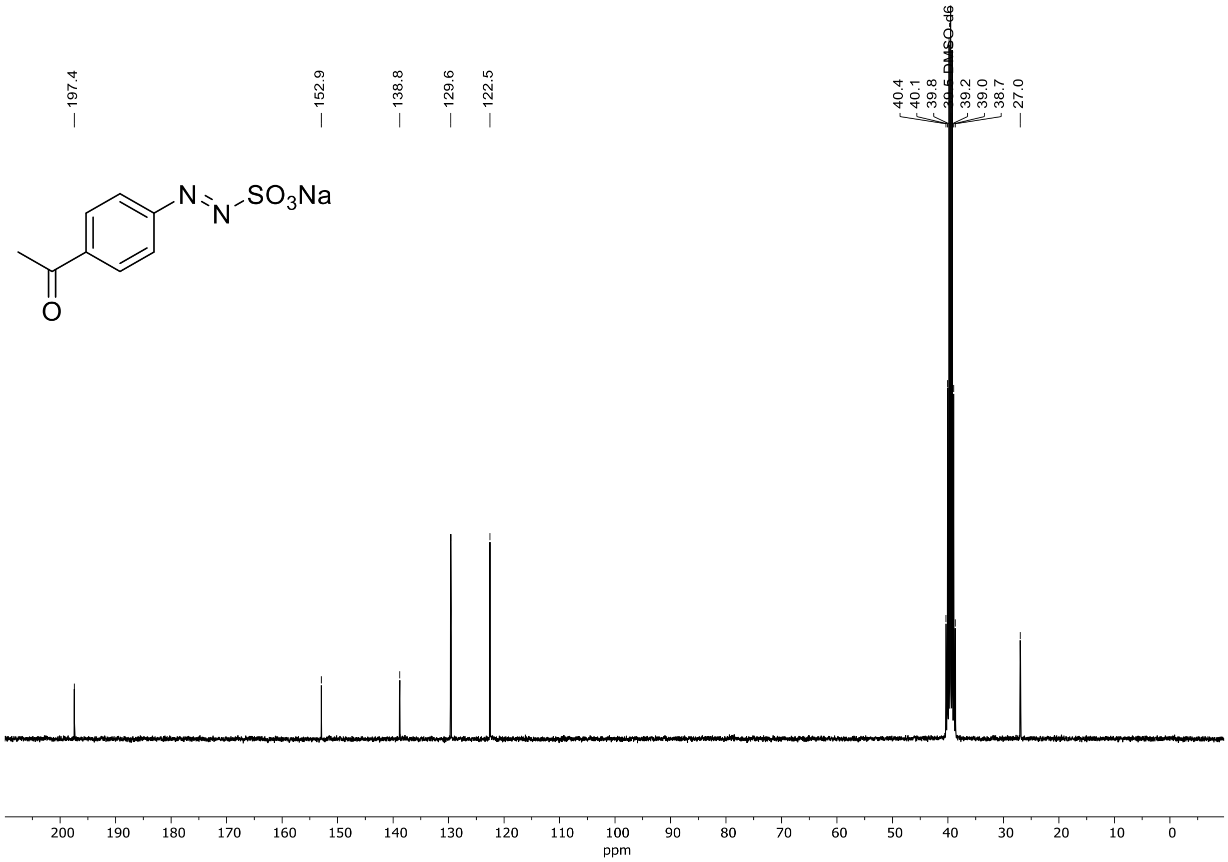

Sodium 2-(4-nitrophenyl)diazene-1-sulfonate (**1e**) ^{1}H NMR (300 MHz, DMSO- d_6).

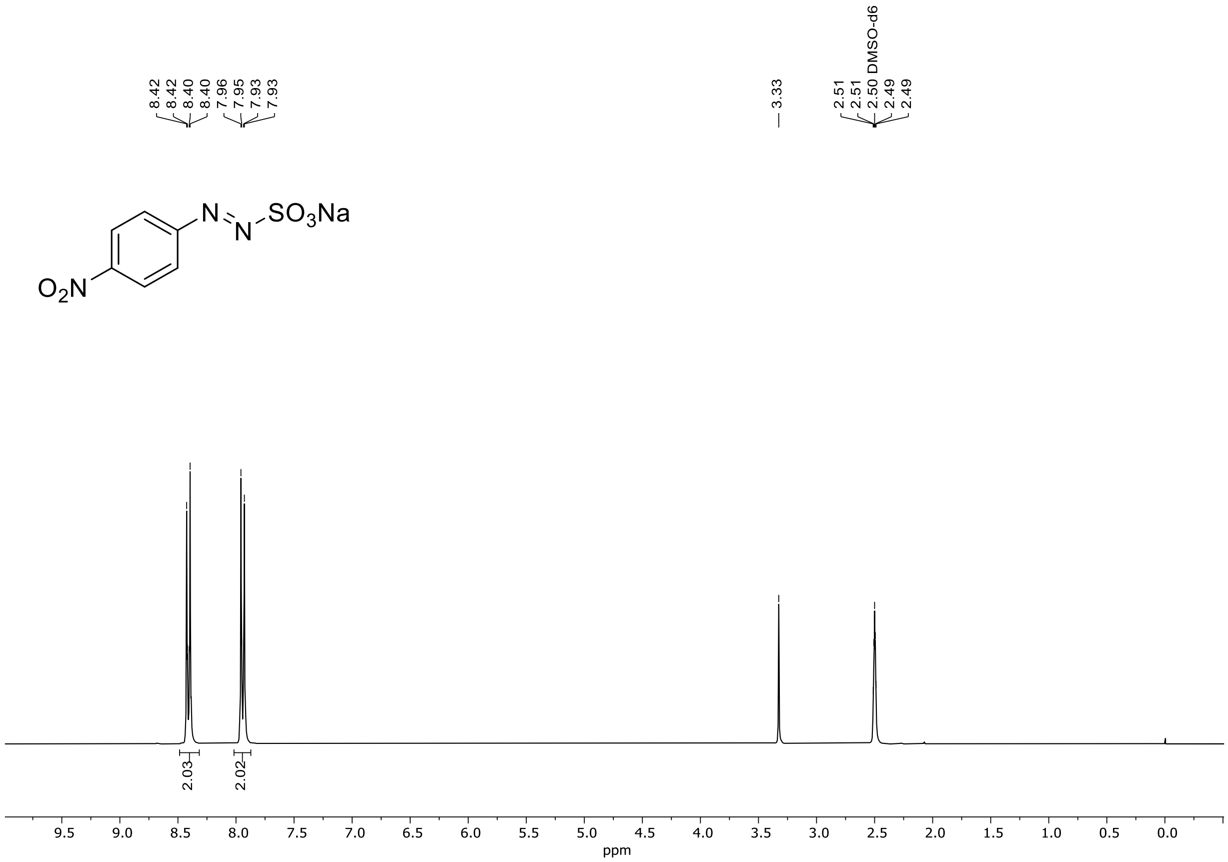

Sodium 2-(4-nitrophenyl)diazene-1-sulfonate (**1e**) $^{13}C\{^{1}H\}$ NMR (75 MHz, DMSO- d_6).

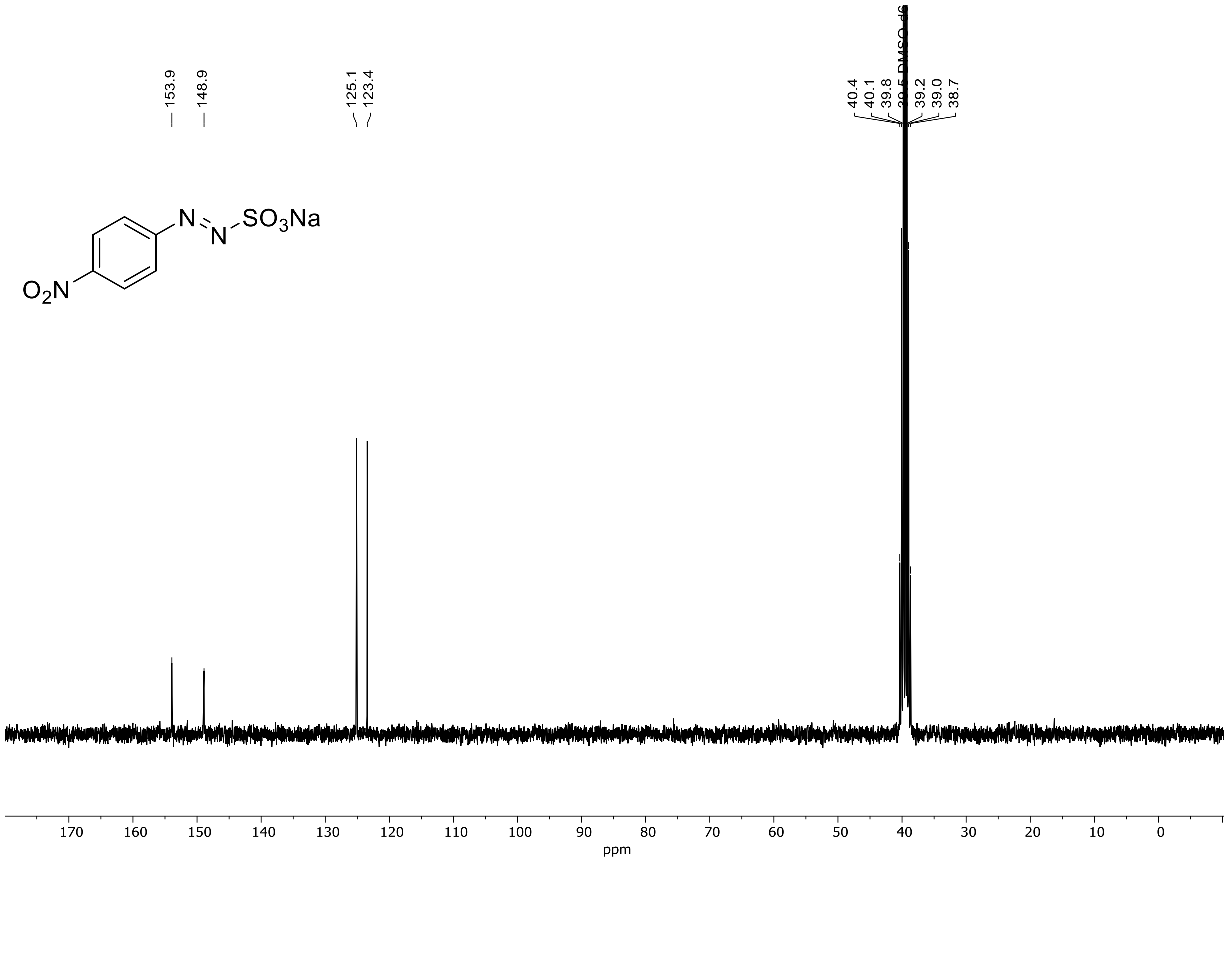

Sodium 2-(4-chlorophenyl)diazene-1-sulfonate (1f) ^{1}H NMR (300 MHz, DMSO- d_6).

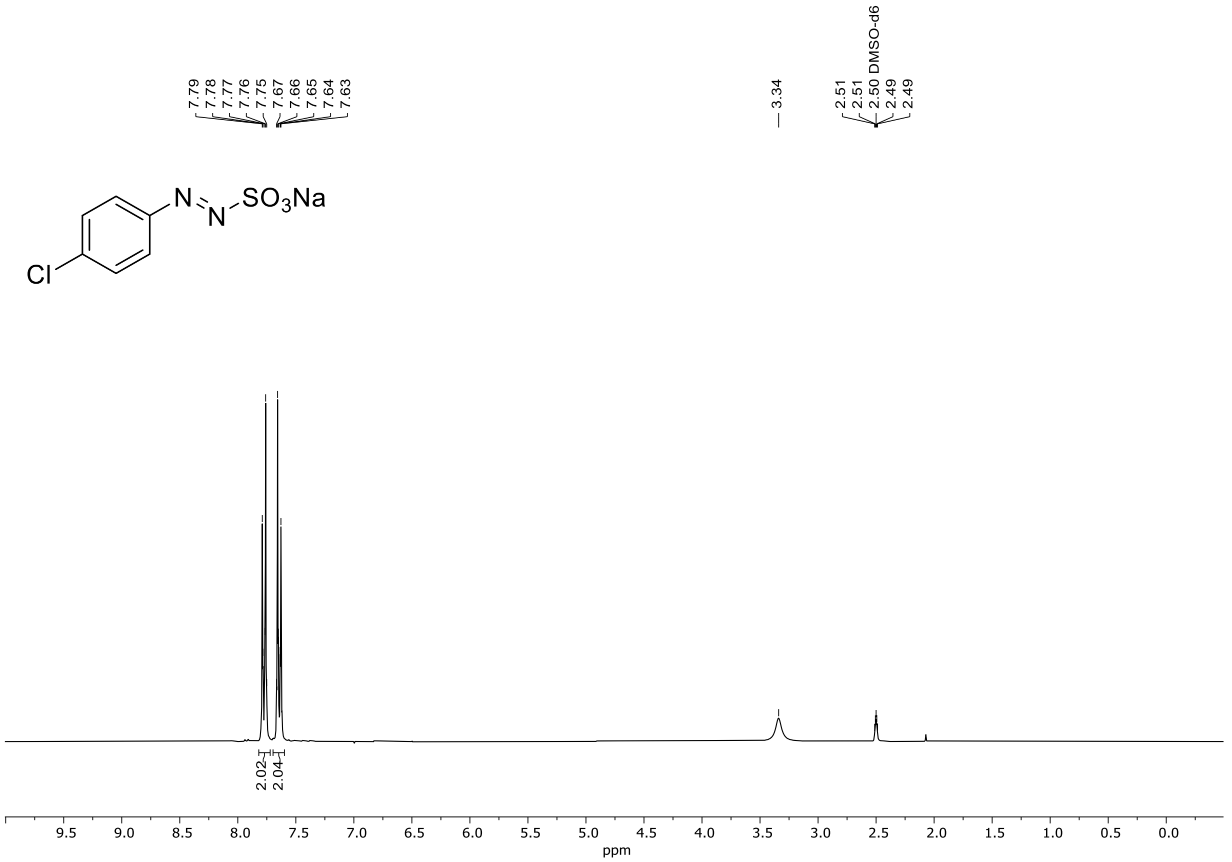

Sodium 2-(4-chlorophenyl)diazene-1-sulfonate (1f) $^{13}C\{^{1}H\}$ NMR (75 MHz, DMSO- d_6).

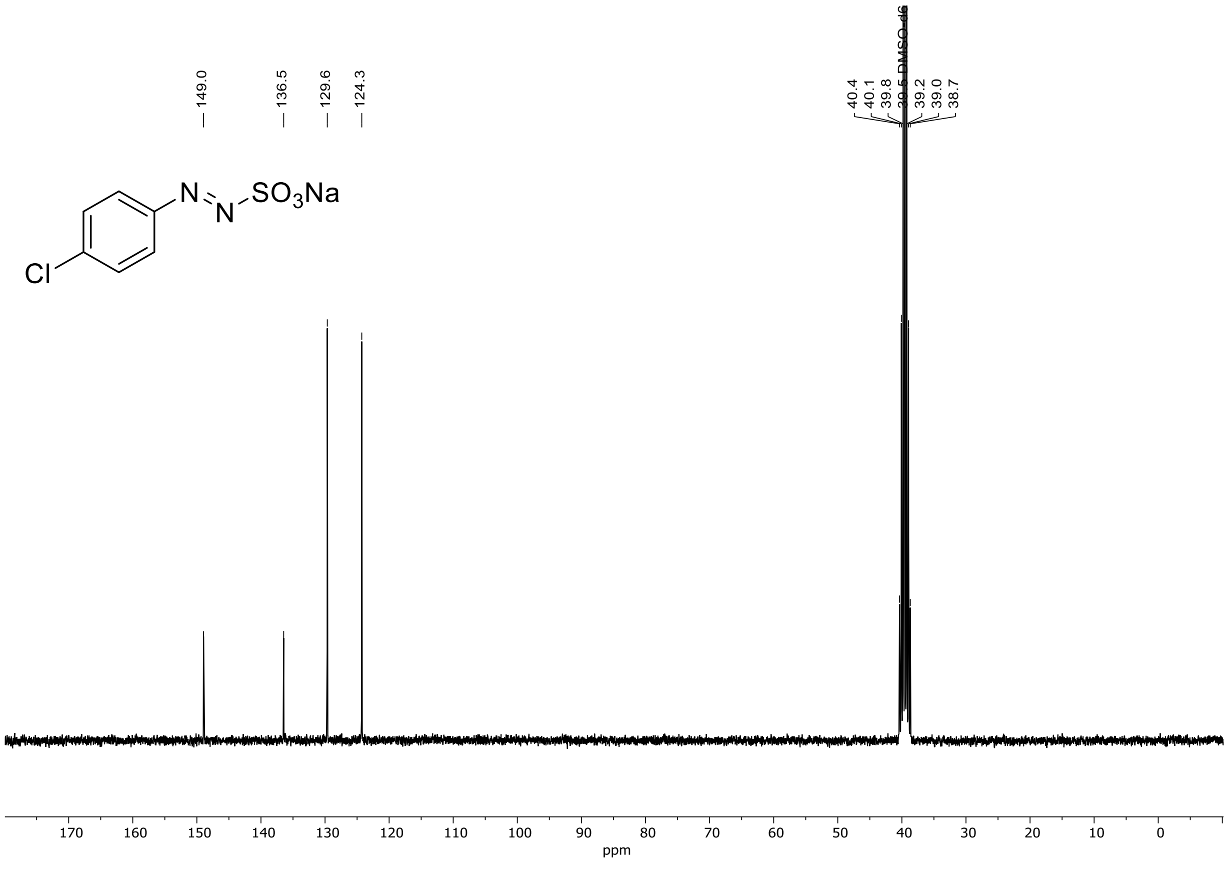

Sodium 2-(4-methylphenyl)diazene-1-sulfonate (**1g**) ^{1}H NMR (300 MHz, DMSO- d_6).

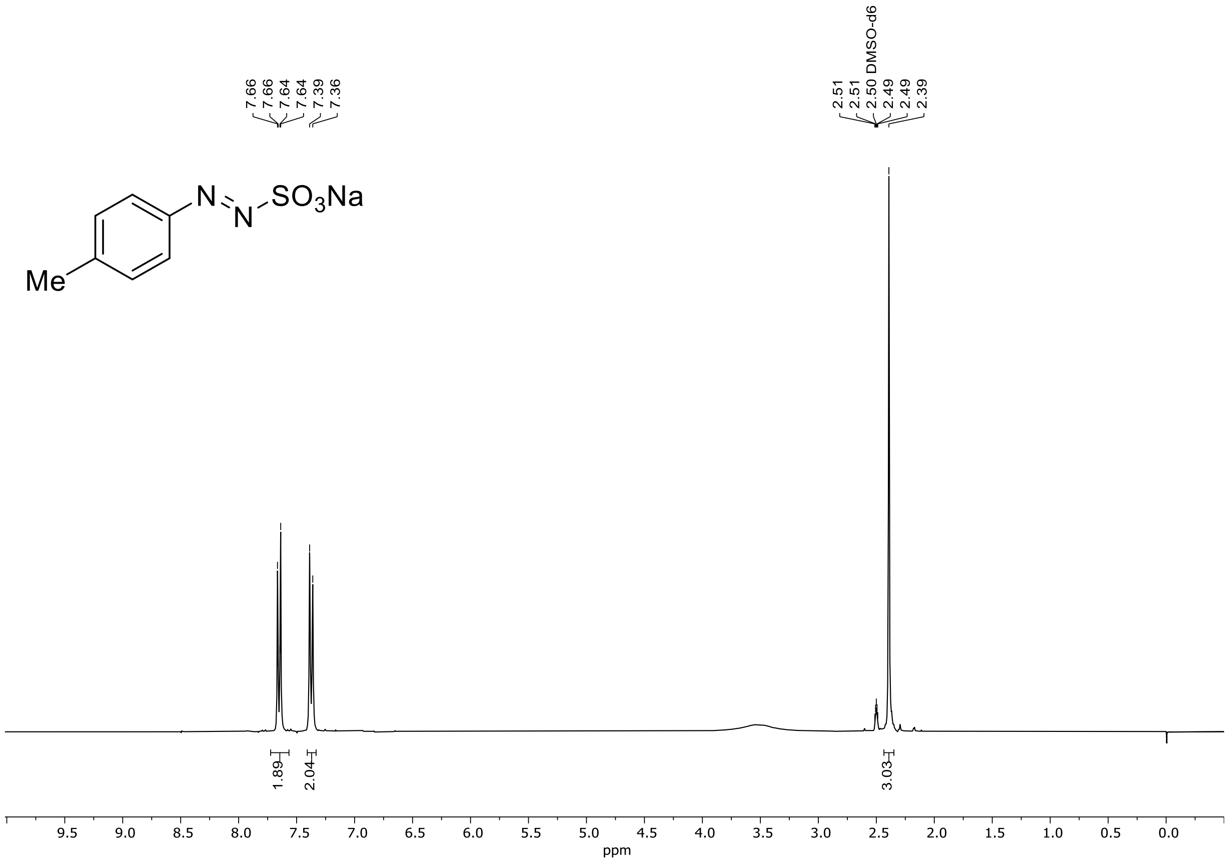

Sodium 2-(4-methylphenyl)diazene-1-sulfonate (**1g**) ^{13}C{^{1}H} NMR (75 MHz, DMSO- d_6).

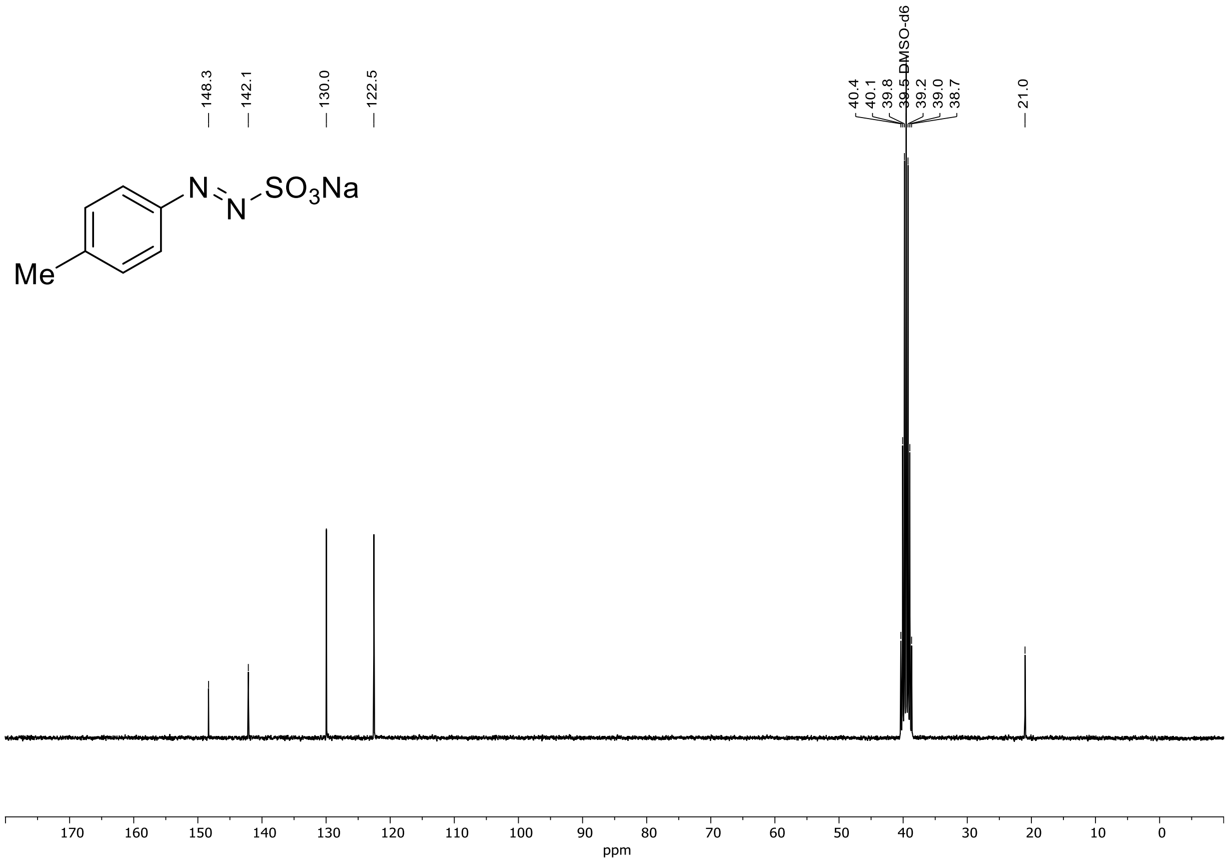

Sodium 2-(3-bromophenyl)diazene-1-sulfonate (**1h**) ^{1}H NMR (300 MHz, DMSO- d_6).

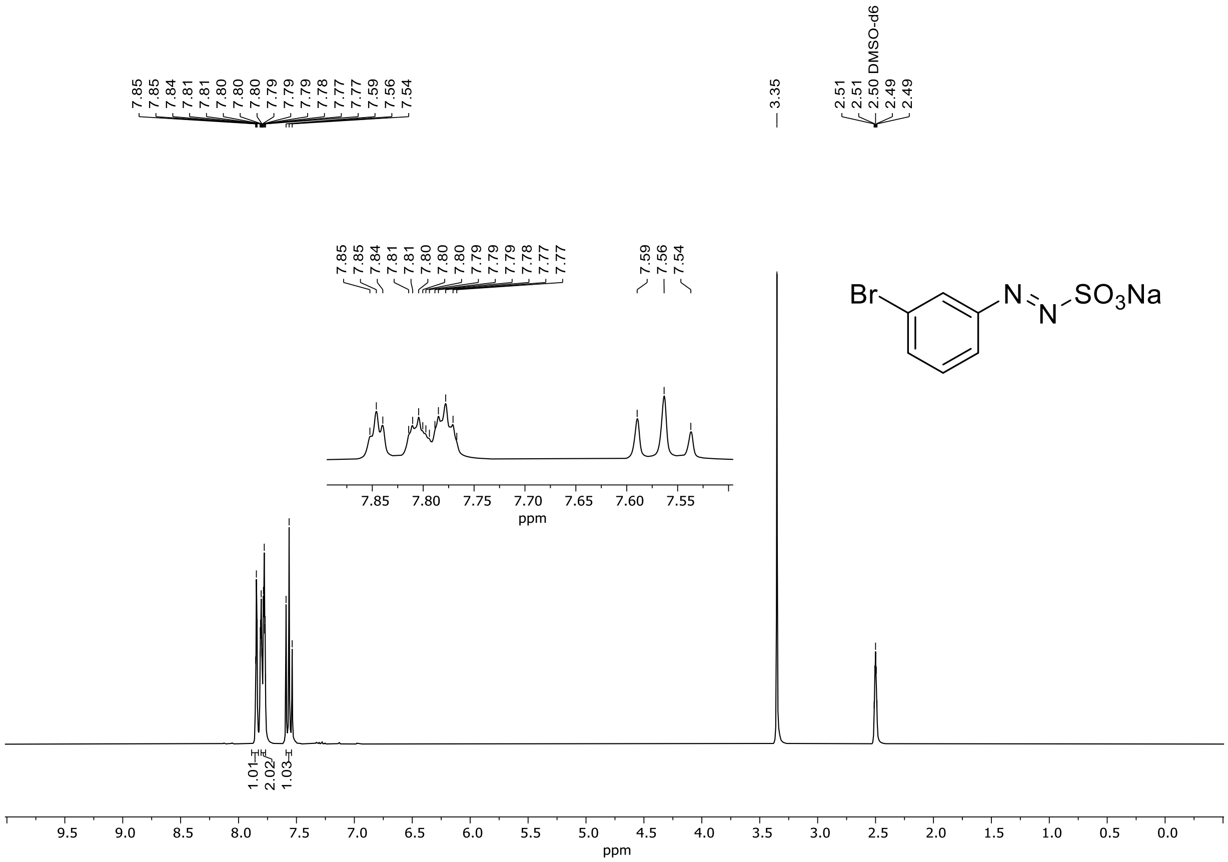

Sodium 2-(3-bromophenyl)diazene-1-sulfonate (**1h**) ^{13}C{^{1}H} NMR (75 MHz, DMSO- d_6).

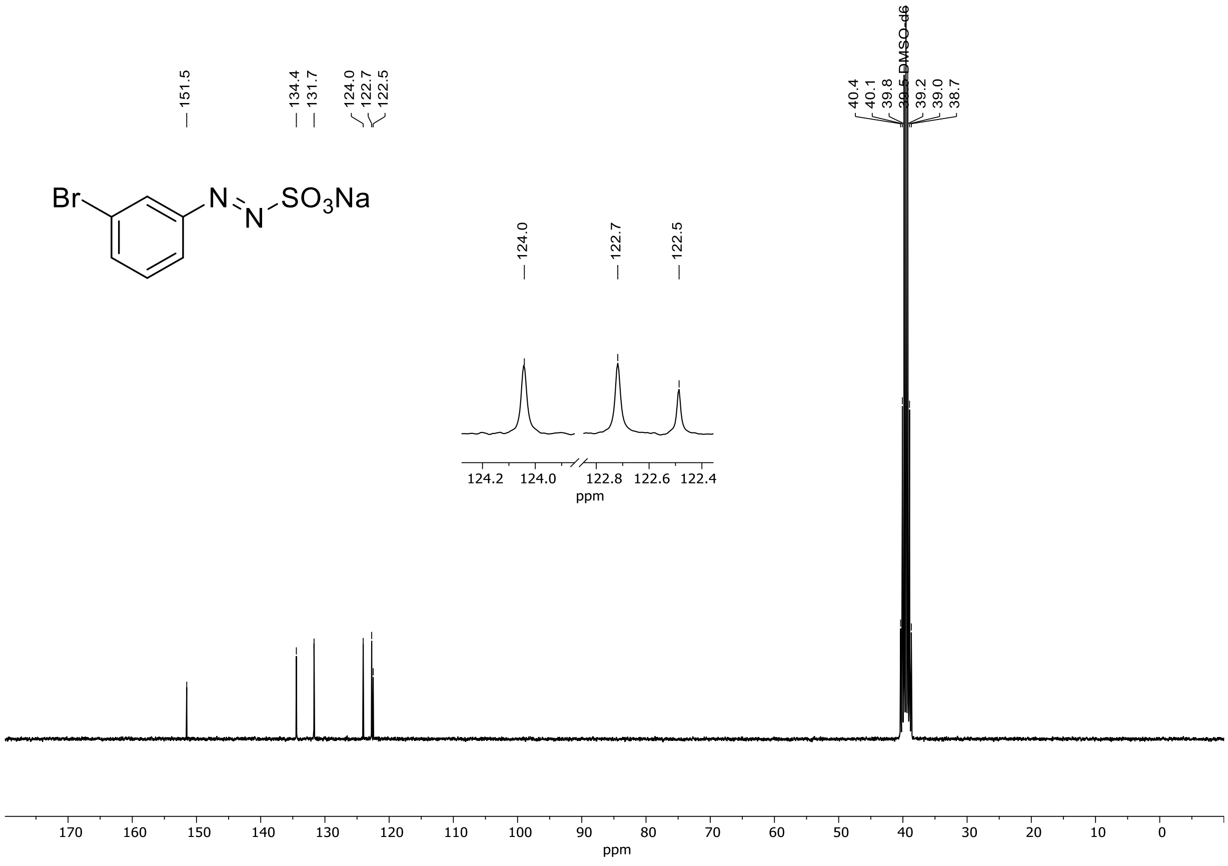

Sodium 2-(3-methoxyphenyl)diazene-1-sulfonate (**1i**) ^{1}H NMR (300 MHz, DMSO- d_6).

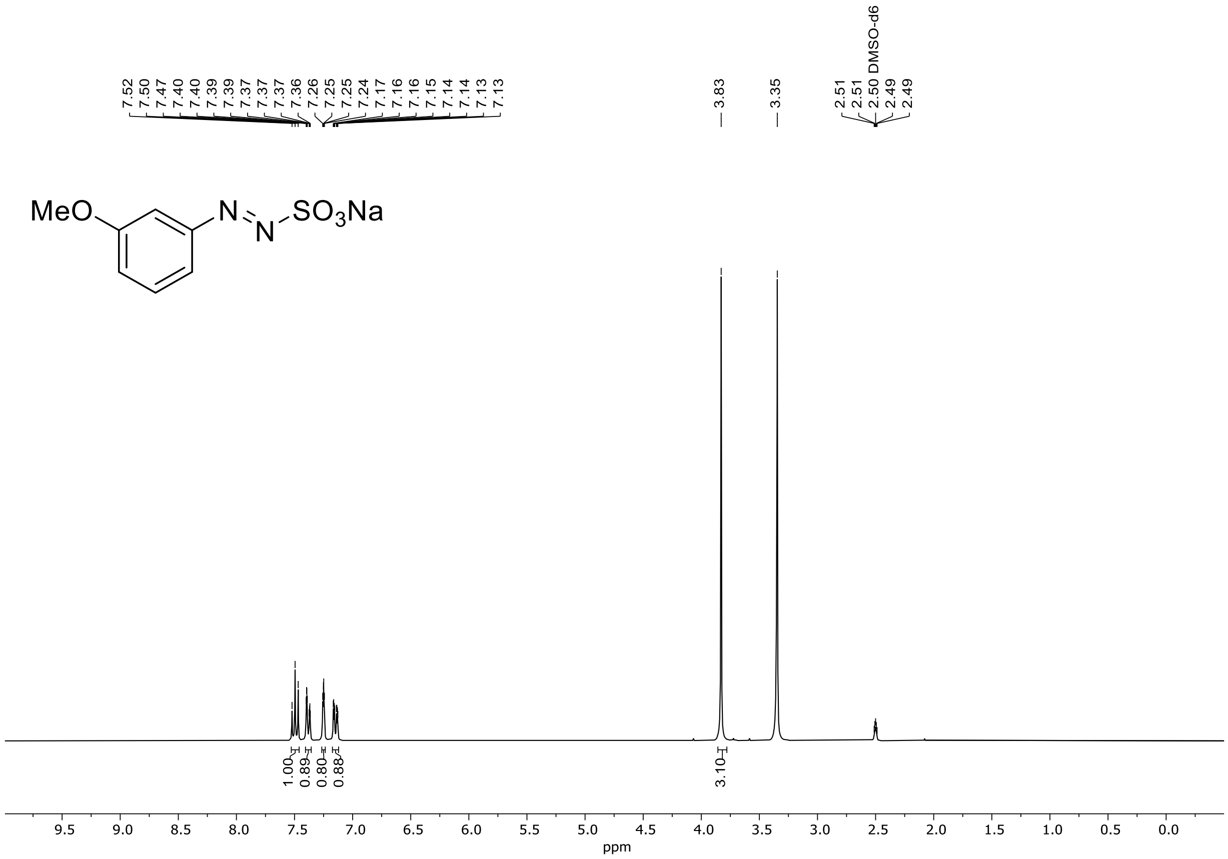

Sodium 2-(3-methoxyphenyl)diazene-1-sulfonate (**1i**) ^{13}C{^{1}H} NMR (75 MHz, DMSO- d_6).

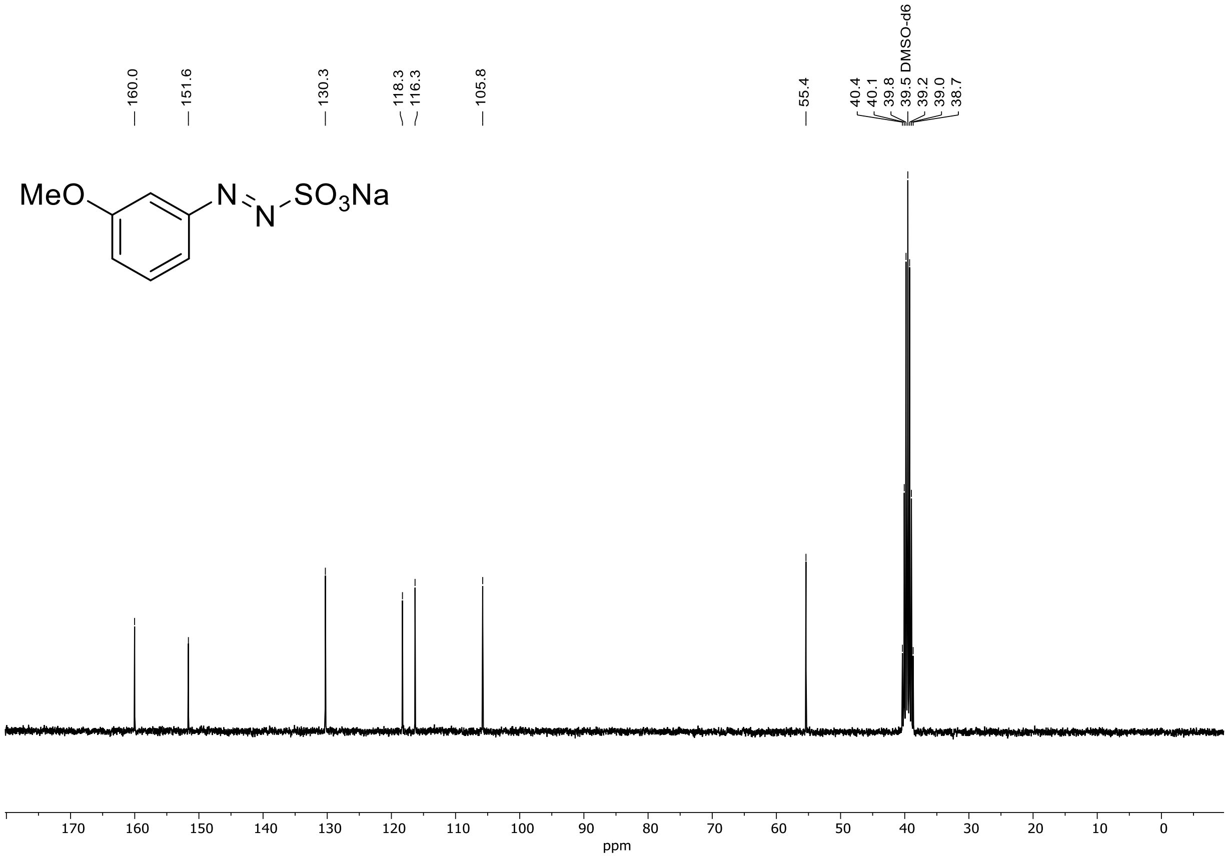

Sodium 2-(2-bromophenyl)diazene-1-sulfonate (**1j**) ^{1}H NMR (300 MHz, DMSO- d_6).

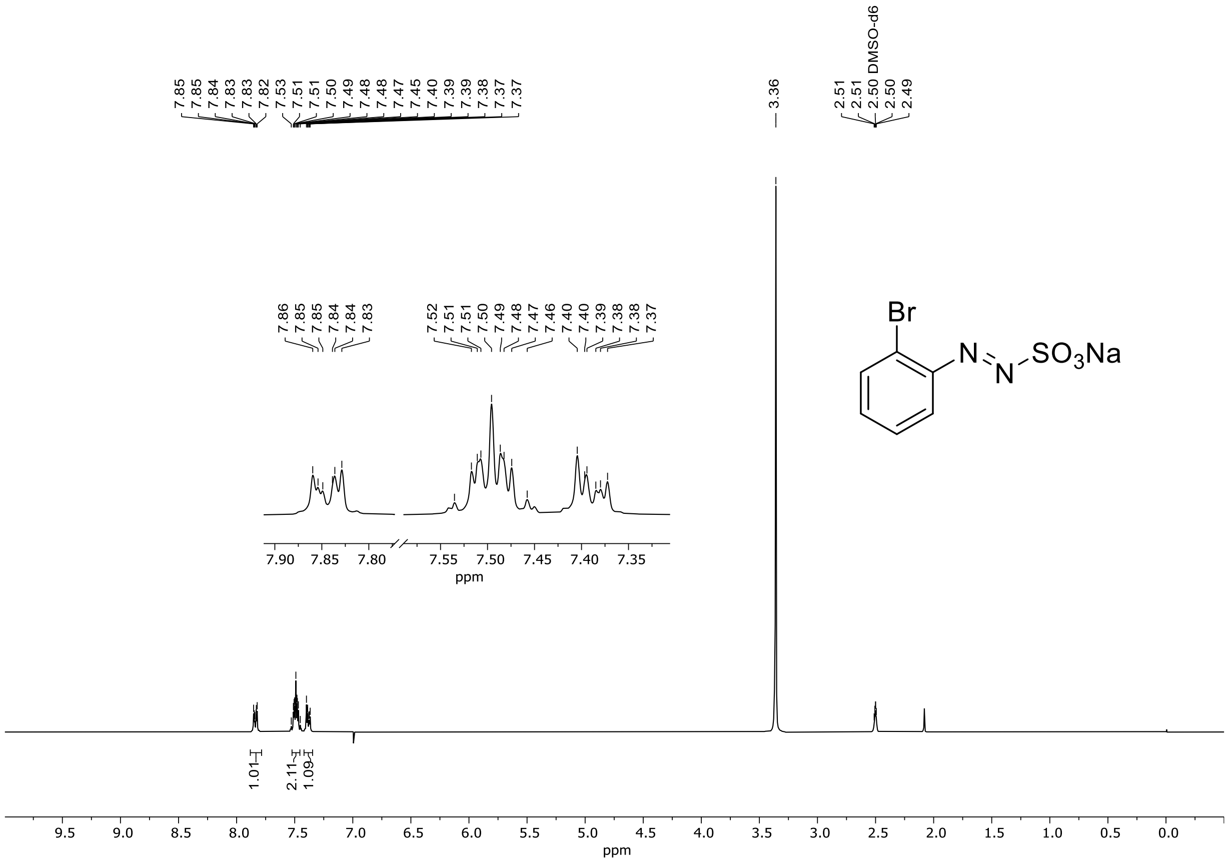

Sodium 2-(2-bromophenyl)diazene-1-sulfonate (**1j**) $^{13}C\{^{1}H\}$ NMR (75 MHz, DMSO- d_6).

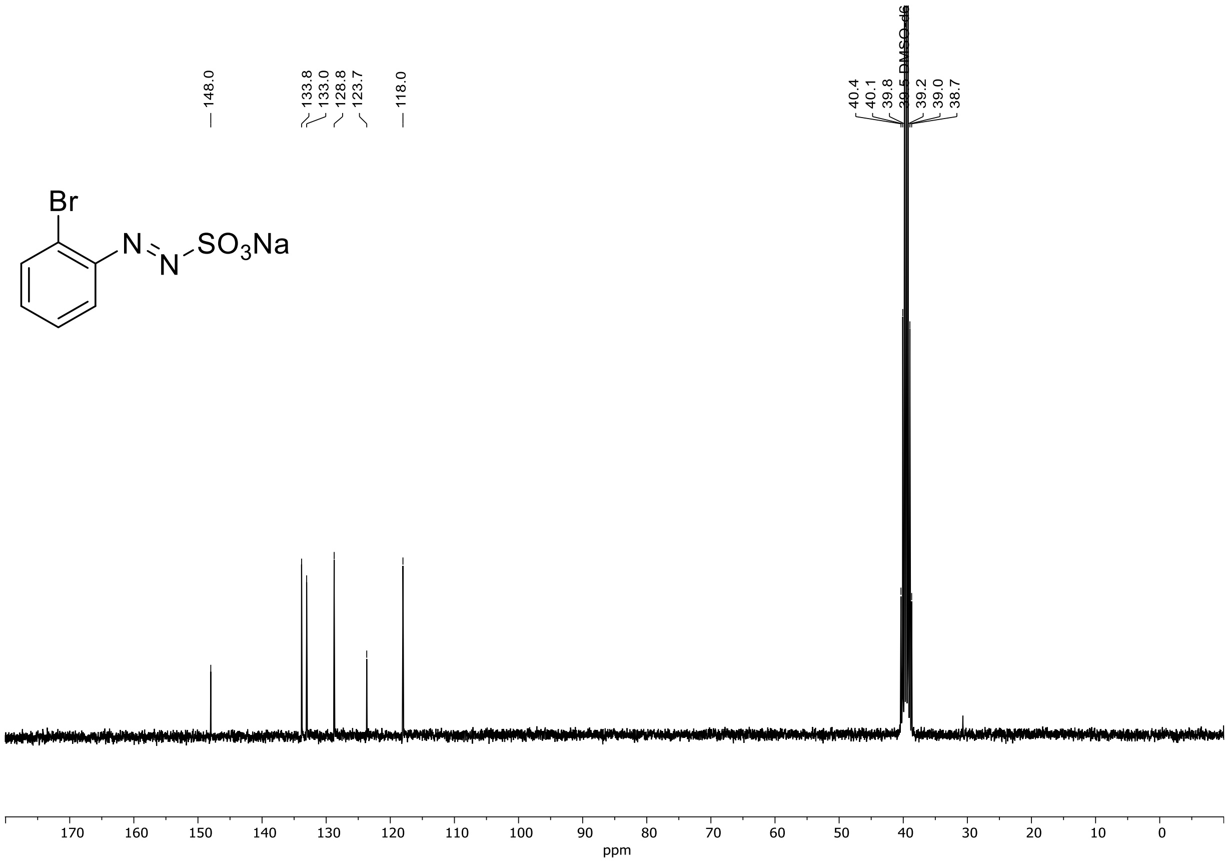

Sodium 2-(2-phenoxyphenyl)diazene-1-solfonate (**1k**) ^{1}H NMR (300 MHz, DMSO- d_6).

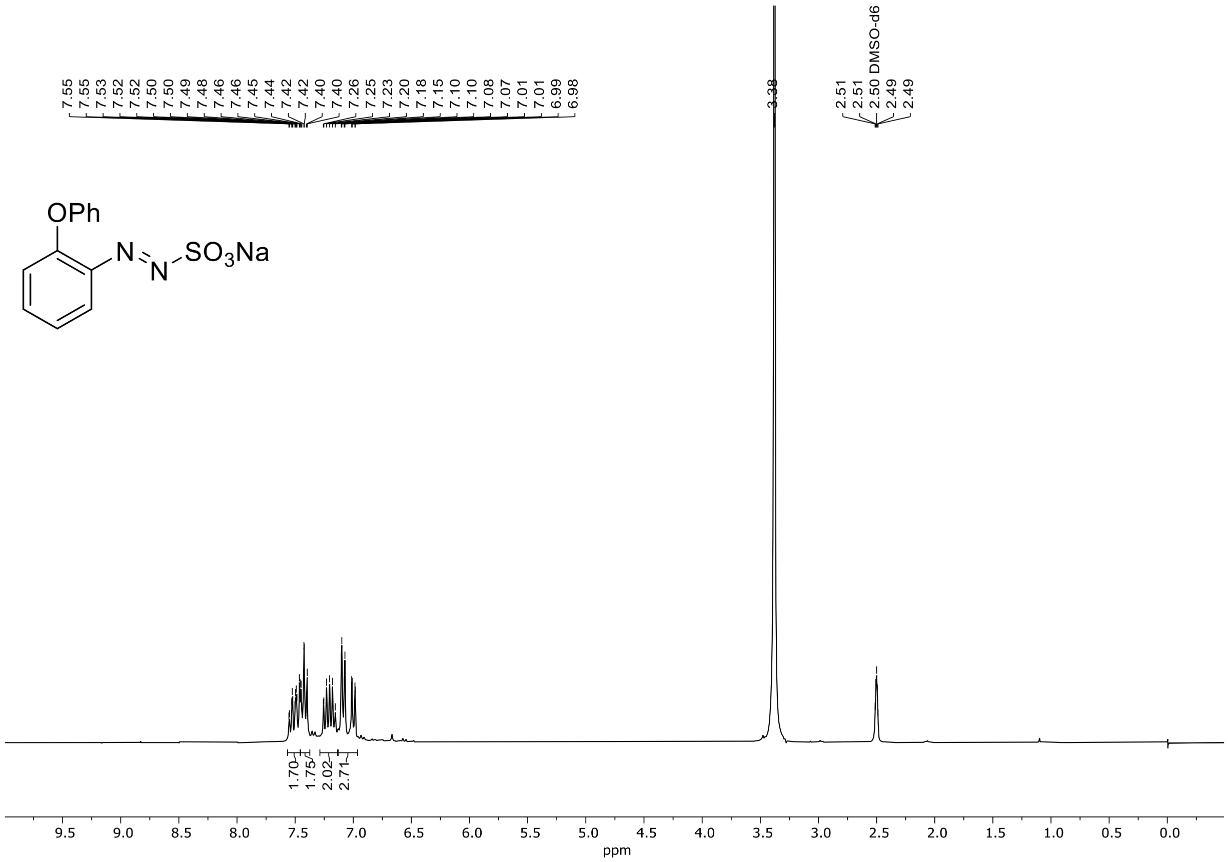

Sodium 2-(2-phenoxyphenyl)diazene-1-solfonate (1k) $^{13}C\{^{1}H\}$ NMR (75 MHz, DMSO- d_6).

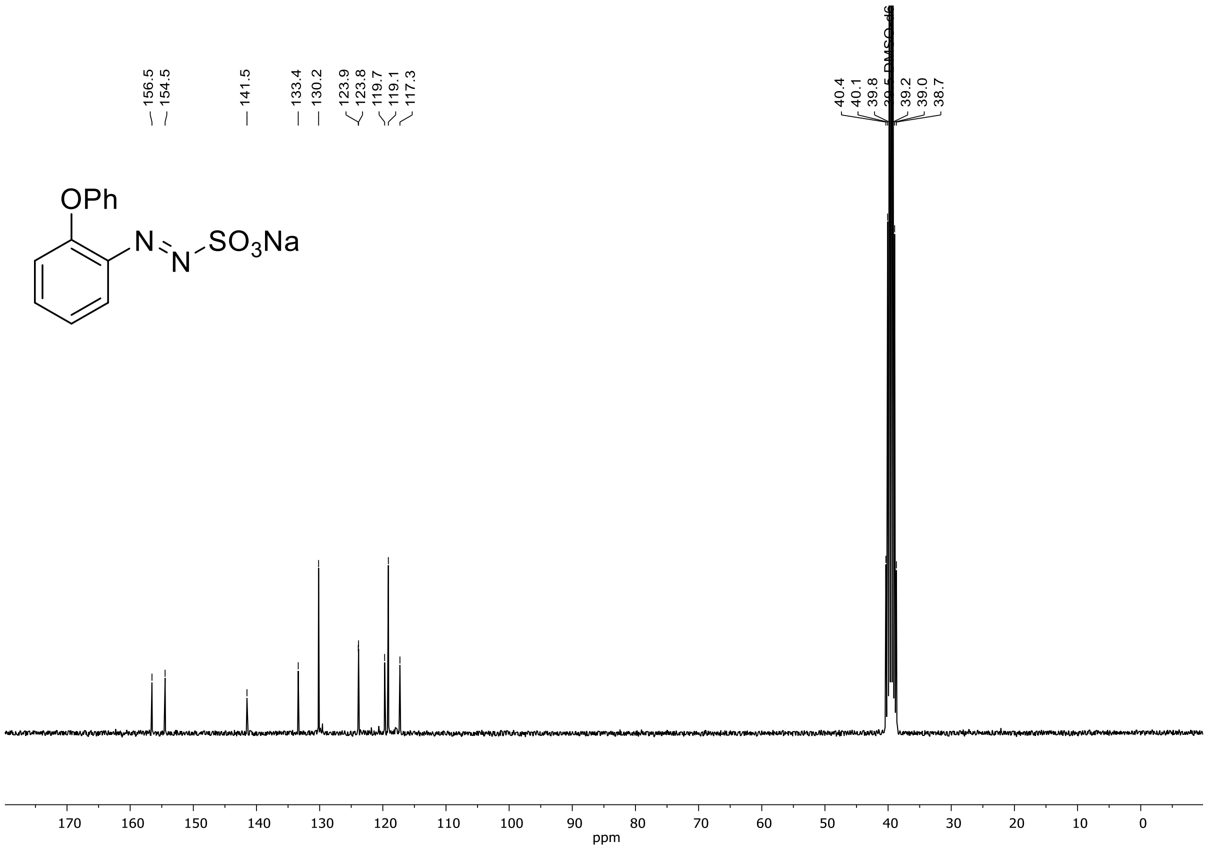

Sodium 2-(2-methylthiophenyl)diazene-1-solfonate (**1l**) ^{1}H NMR (300 MHz, DMSO- d_6).

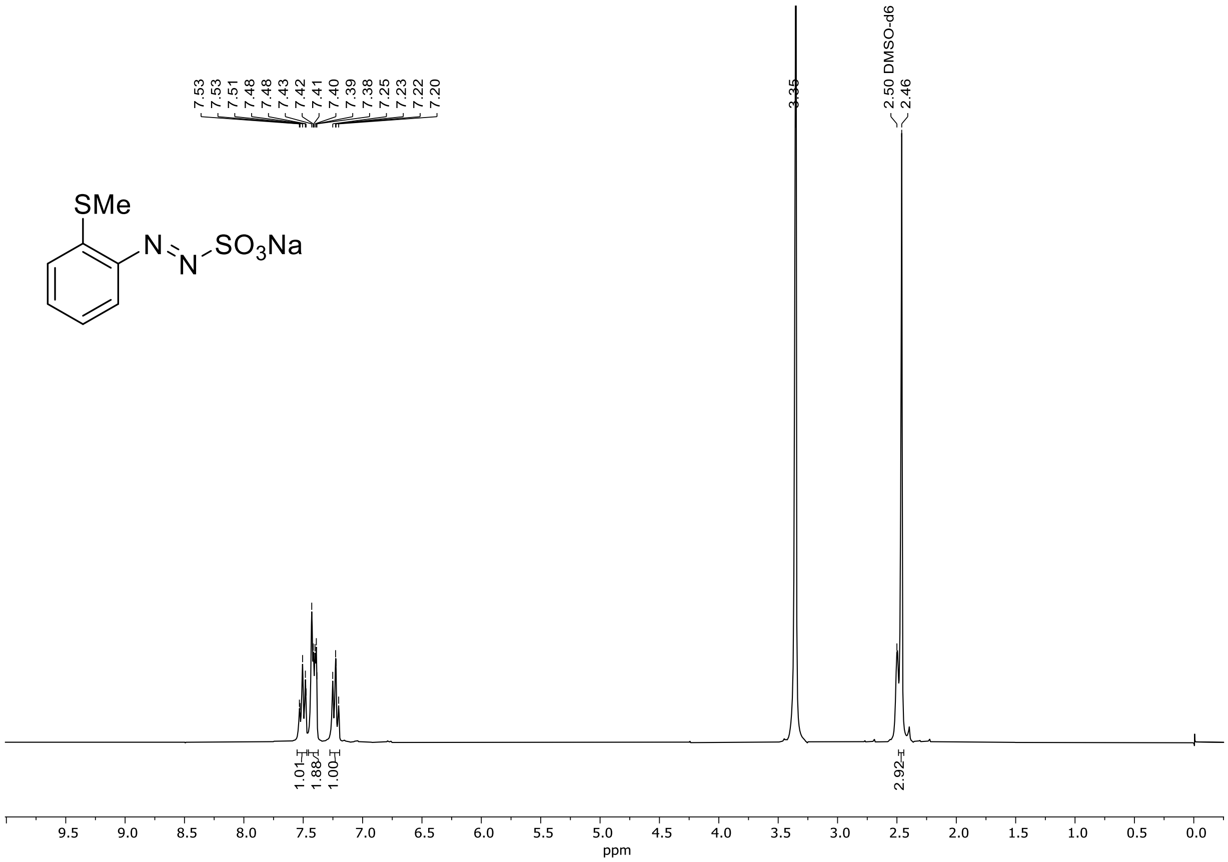

Sodium 2-(2-methylthiophenyl)diazene-1-solfonate (**1l**) ^{13}C{^{1}H} NMR (75 MHz, DMSO- d_6).

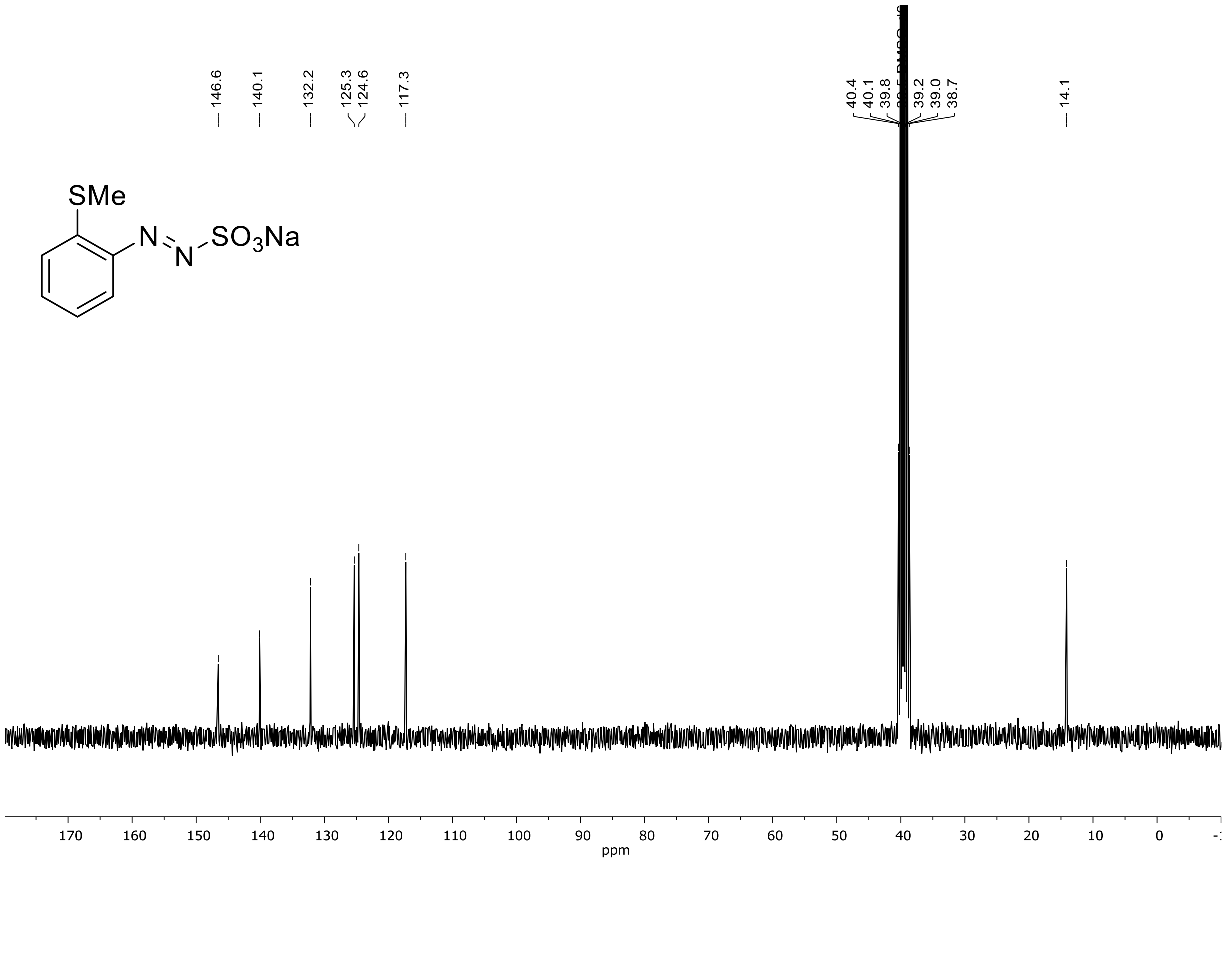

Sodium 2-(2,6-dimethylphenyl)diazene-1-solfonate (**1m**) ^{1}H NMR (300 MHz, DMSO- d_6).

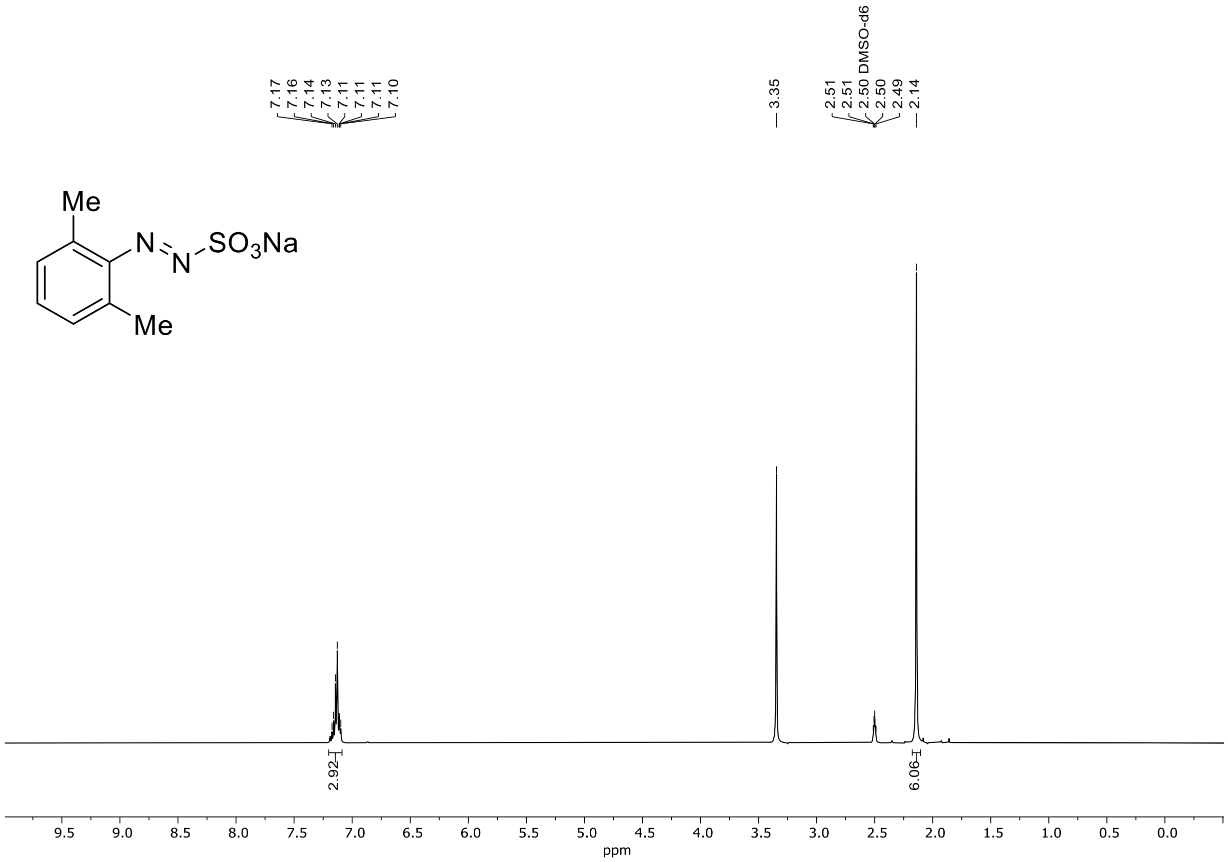

Sodium 2-(2,6-dimethylphenyl)diazene-1-solfonate (**1m**) ^{13}C{^{1}H} NMR (75 MHz, DMSO- d_6).

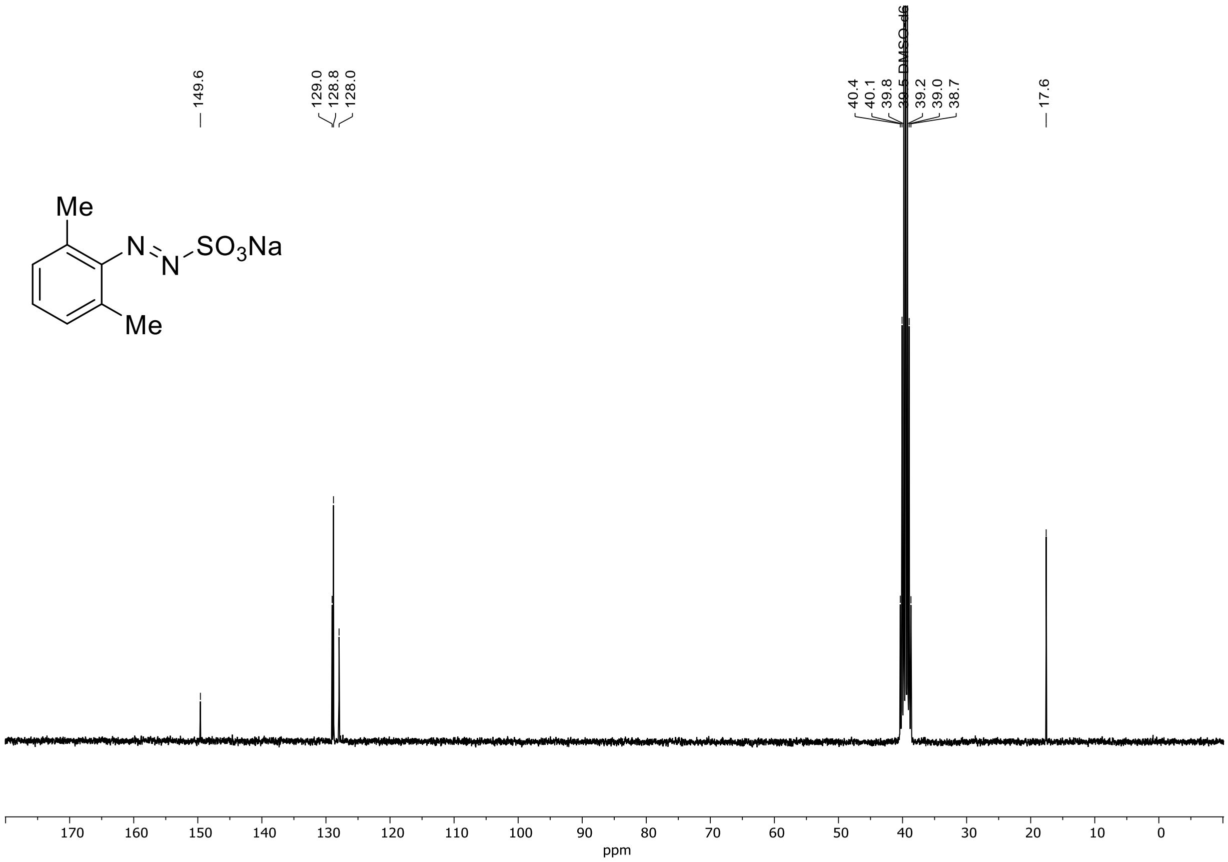

Sodium 2-(2,4,6-trimethylphenyll)diazene-1-sulfonate (1n) ^{1}H NMR (300 MHz, DMSO- d_6).

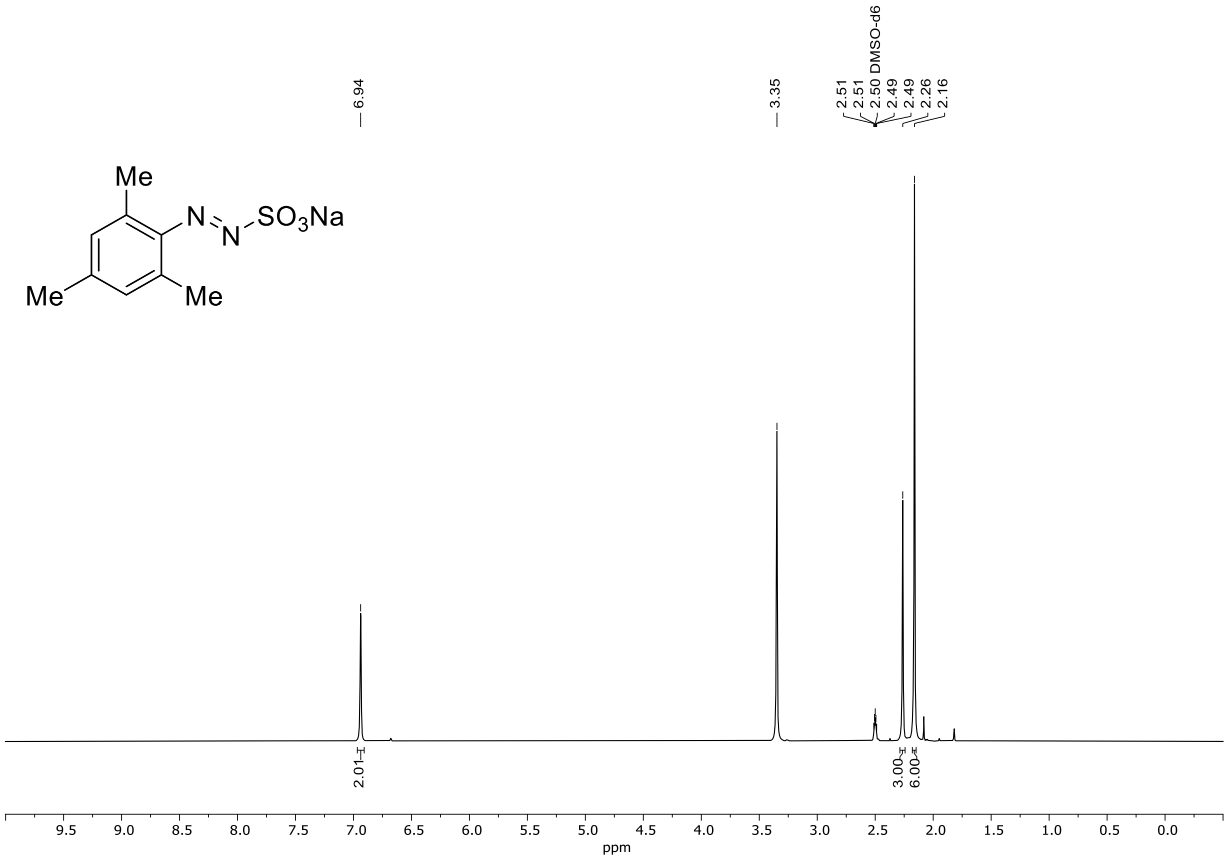

Sodium 2-(2,4,6-trimethylphenyll)diazene-1-sulfonate (1n) ^{13}C{^{1}H} NMR (75 MHz, DMSO-d_6).

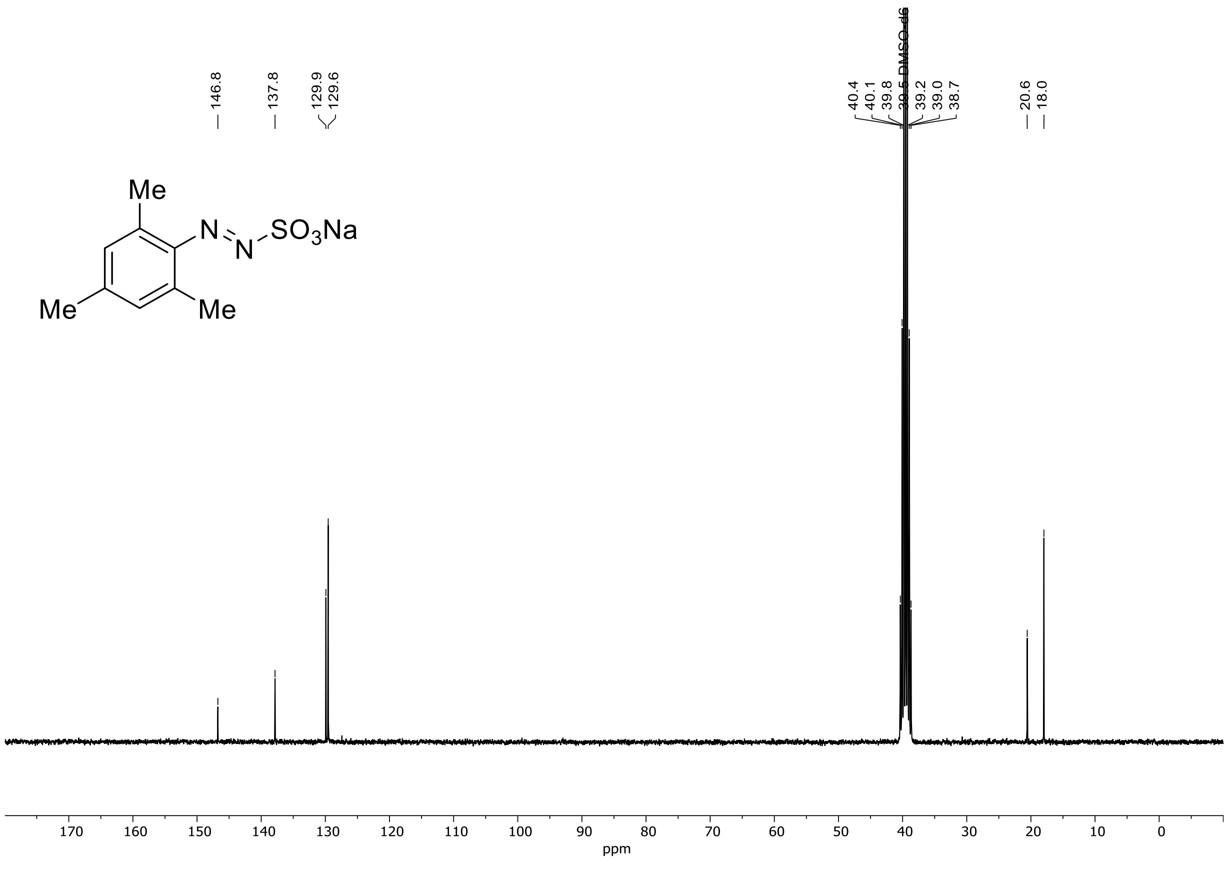

Sodium 2-(2-chloro-4-bromophenyl)diazene-1-solfonate (**1o**) ^{1}H NMR (300 MHz, DMSO- d_6).

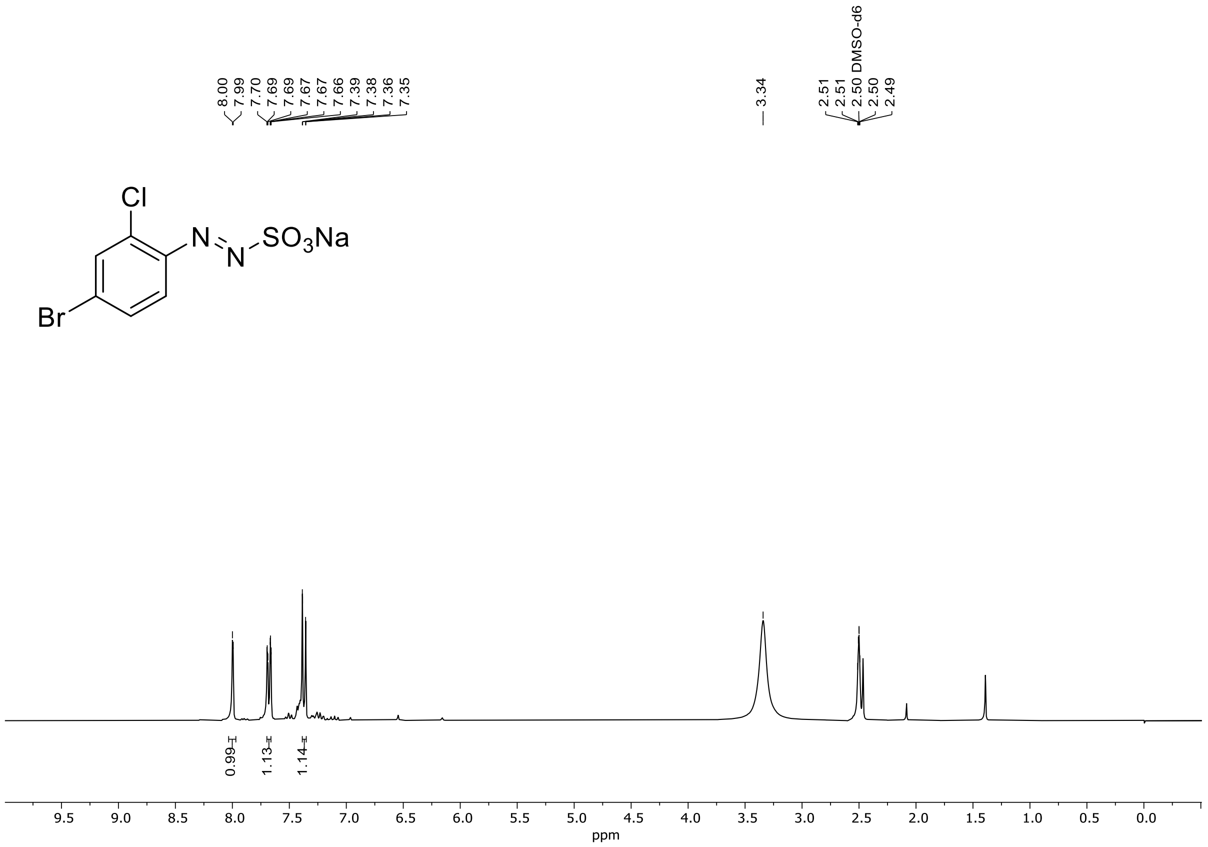

Sodium 2-(2-chloro-4-bromophenyl)diazene-1-solfonate (**1o**) ^{13}C{^{1}H} NMR (75 MHz, DMSO-d_6).

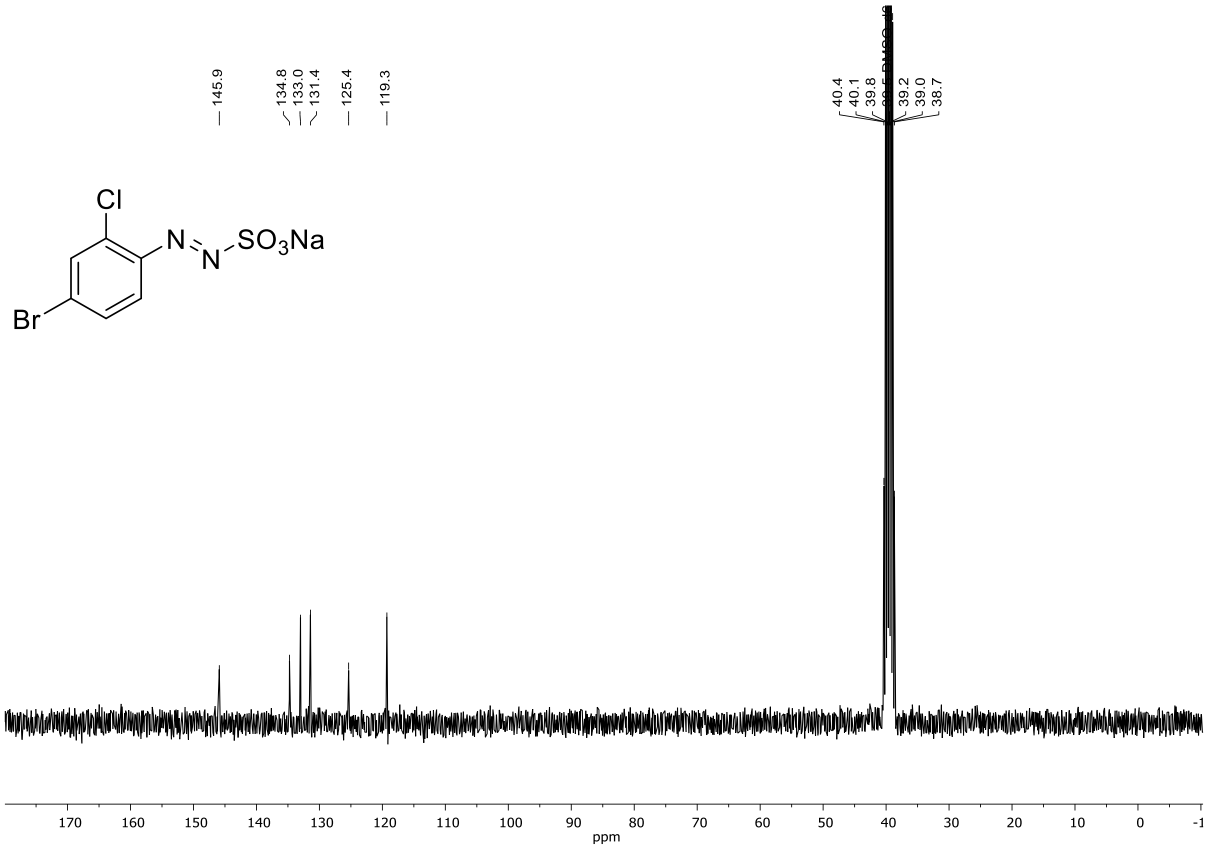

4-((2,2,6,6-Tetramethylpiperidin-1-yl)oxy)benzonitrile (8a) ^{1}H NMR (300 MHz, Chloroform-*d*)

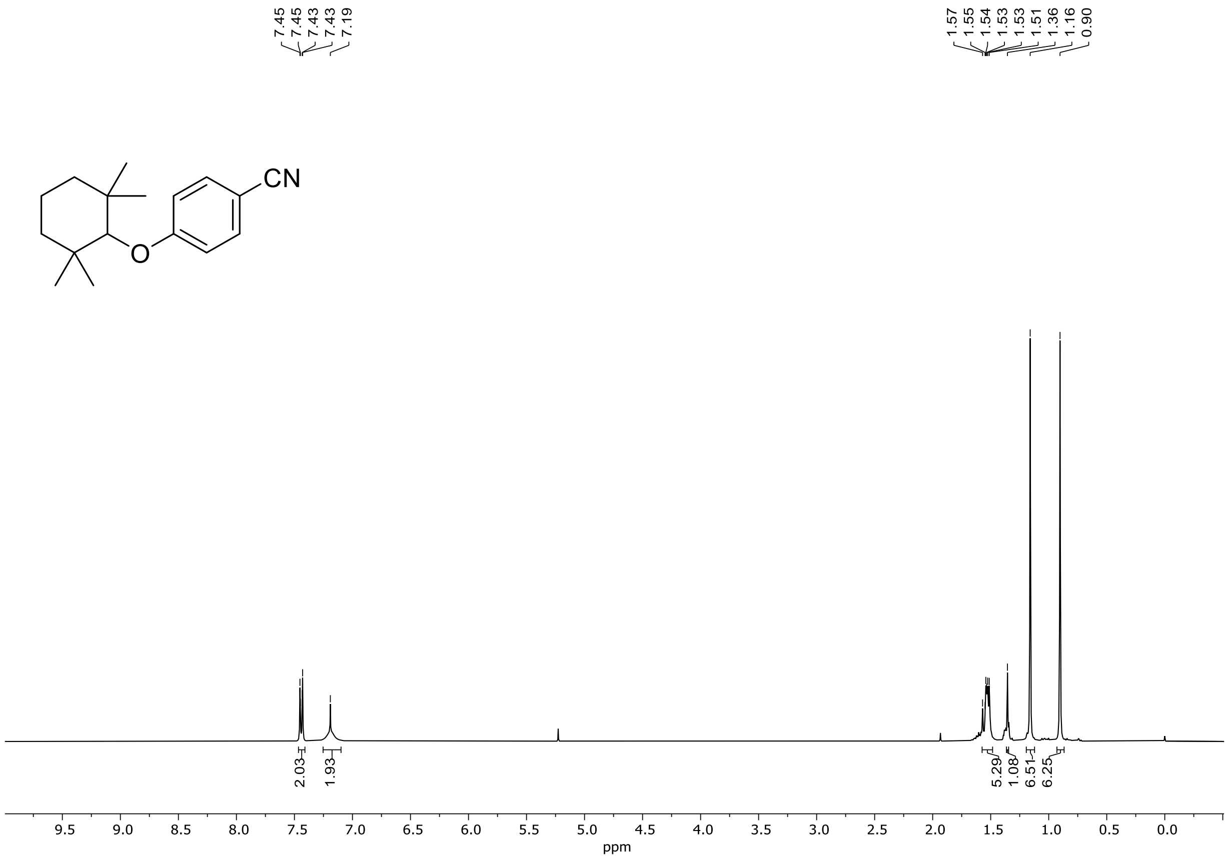

4-((2,2,6,6-Tetramethylpiperidin-1-yl)oxy)benzonitrile (8a) ^{13}C{^{1}H} NMR (75 MHz, Chloroform-*d*)

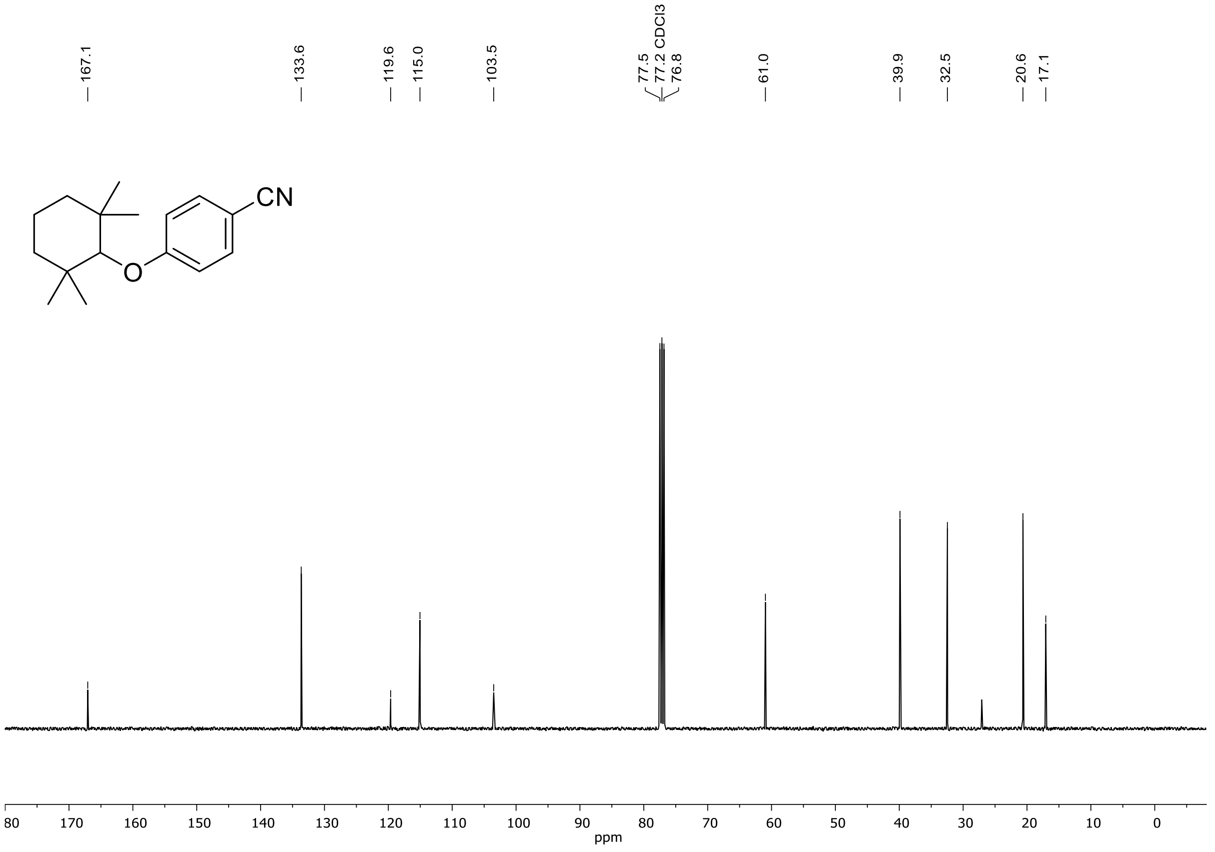

3-((4-Methoxyphenyl)diazenyl)-1H-indole (9c) ^{1}H NMR (300 MHz, Chloroform-*d*)

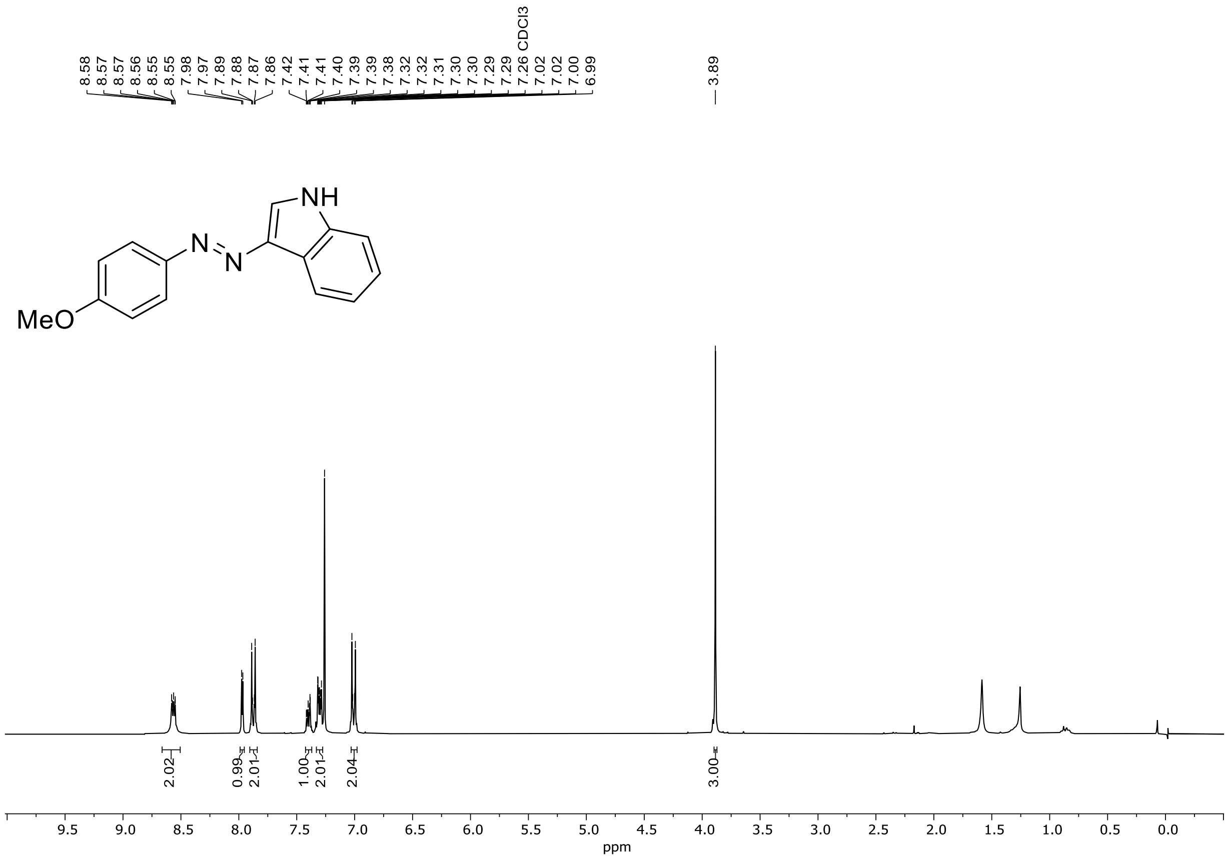

2-((4-Methoxyphenyl)diazenyl)-1H-indole (9c) ^{13}C{^{1}H} NMR (75 MHz, Chloroform-*d*)

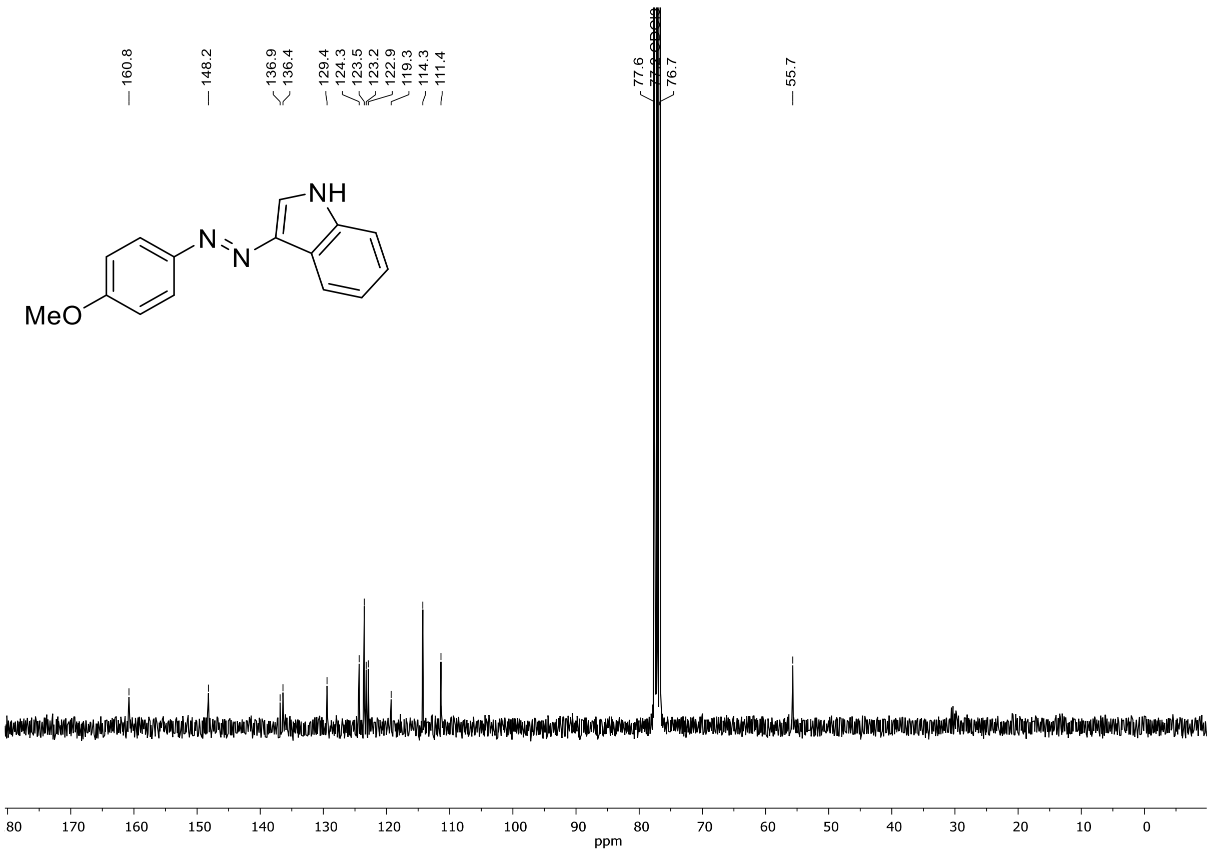

2-(4-Methoxyphenyl)-3-((4-methoxyphenyl)diazenyl)-1H-indole (10c) ^{1}H NMR (300 MHz, Chloroform-*d*)

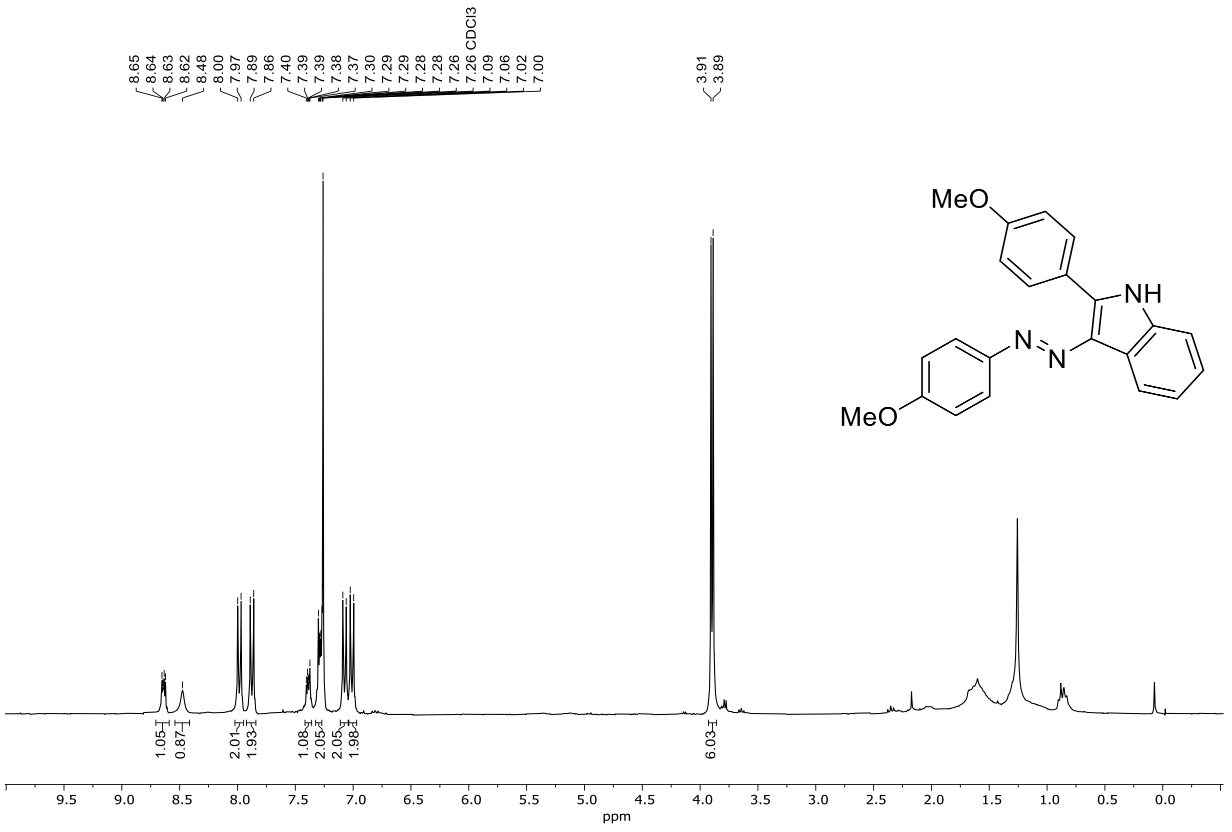

2-(4-Methoxyphenyl)-3-((4-methoxyphenyl)diazenyl)-1H-indole (10c) ^{13}C{^{1}H} NMR (75 MHz, Chloroform- *d*)

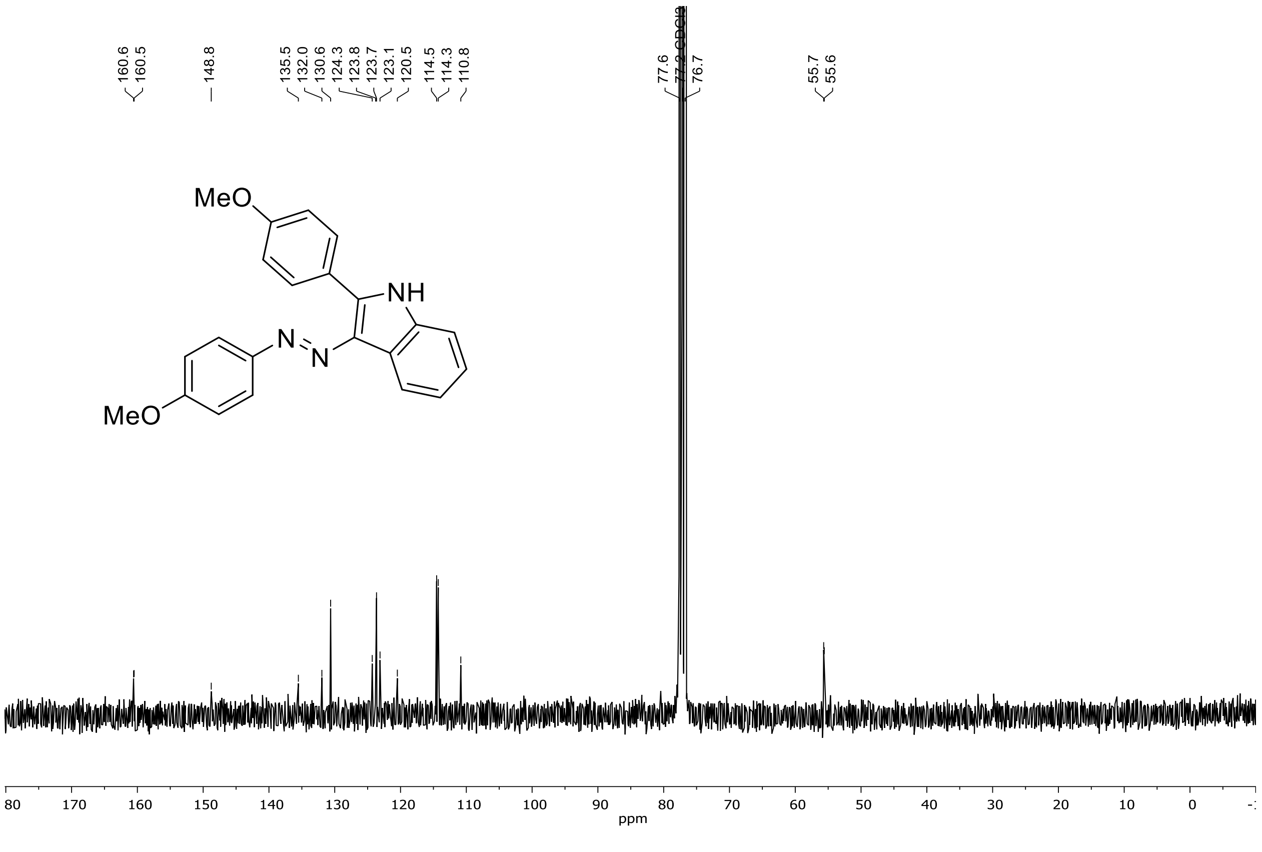

Supplement: Supplementary file 1 [file jo5c00314_si_001.pdf]
